# Phosphodiesterase in heart and vessels: from physiology to diseases

**DOI:** 10.1152/physrev.00015.2023

**Published:** 2023-11-16

**Authors:** Qin Fu, Ying Wang, Chen Yan, Yang K. Xiang

**Affiliations:** ^1^Department of Pharmacology, School of Basic Medicine, Tongji Medical College, Huazhong University of Science and Technology, Wuhan, China; ^2^The Key Laboratory for Drug Target Research and Pharmacodynamic Evaluation of Hubei Province, Wuhan, China; ^3^Department of Pharmacology, School of Medicine, Southern University of Science and Technology, Shenzhen, China; ^4^Aab Cardiovascular Research Institute, University of Rochester Medical Center, Rochester, New York, United States; ^5^Department of Pharmacology, University of California at Davis, Davis, California, United States; ^6^Department of Veterans Affairs Northern California Healthcare System, Mather, California, United States

**Keywords:** cardiovascular disease, cyclic nucleotides, heart failure, phosphodiesterase, vessels

## Abstract

Phosphodiesterases (PDEs) are a superfamily of enzymes that hydrolyze cyclic nucleotides, including cyclic adenosine monophosphate (cAMP) and cyclic guanosine monophosphate (cGMP). Both cyclic nucleotides are critical secondary messengers in the neurohormonal regulation in the cardiovascular system. PDEs precisely control spatiotemporal subcellular distribution of cyclic nucleotides in a cell- and tissue-specific manner, playing critical roles in physiological responses to hormone stimulation in the heart and vessels. Dysregulation of PDEs has been linked to the development of several cardiovascular diseases, such as hypertension, aneurysm, atherosclerosis, arrhythmia, and heart failure. Targeting these enzymes has been proven effective in treating cardiovascular diseases and is an attractive and promising strategy for the development of new drugs. In this review, we discuss the current understanding of the complex regulation of PDE isoforms in cardiovascular function, highlighting the divergent and even opposing roles of PDE isoforms in different pathogenesis.

## Graphical abstract

**Figure:**
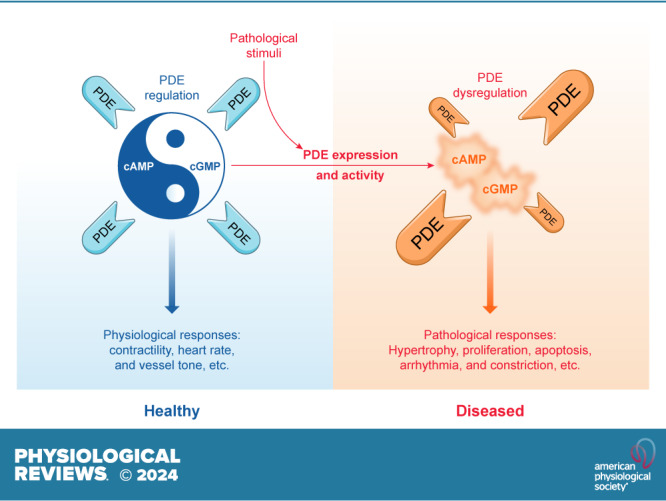

CLINICAL HIGHLIGHTS Phosphodiesterases regulate multiple physiological processes in the cardiovascular system, including cardiac and vessel contraction. Phosphodiesterases are critical components in the signalosomes that precisely control cardiac muscle, smooth muscle, and endothelial cell function in physiological conditions.

Many phosphodiesterases display increased expression and activities in a cell- and tissue-specific manner in pathogenesis. Many have sex-, etiology-, and disease stage-specific regulation, which plays distinct, even opposing, roles in the development of cardiovascular diseases.

Phosphodiesterase 5 inhibitors have been proven effective in erectile dysfunction and pulmonary hypertension. Targeting phosphodiesterase 5 remains an area of intensive interest for additional clinical benefits in diseases such as heart failure with preserved ejection fraction.

Phosphodiesterase 3 inhibitors are effective in acute heart failure and end-stage heart failure, and recent studies aim to improve their utility in managing chronic heart failure and other diseases such as stroke.

Phosphodiesterase 1 inhibitors are effective in stroke, and recent studies aim to explore their utility in managing heart failure with reduced ejection fraction.

Emerging insight into phosphodiesterases 2 and 4 in cardiovascular diseases may provide better strategies to target the enzymes in clinical settings. The development of rational strategies for targeting individual isoforms in a disease-specific manner may allow efficacious therapy with minimal side effects.

## 1. INTRODUCTION

Upon stimulation, activation of G protein-coupled receptors (GPCRs) and natriuretic peptide receptors (NPRs) increases the activity of adenylate cyclases (ACs) and guanylyl cyclases (GCs), which produce cyclic adenosine monophosphate (cAMP) and cyclic guanosine monophosphate (cGMP), respectively. cAMP and cGMP function as critical secondary messengers in neurohormonal regulation in the cardiovascular system. Phosphodiesterases (PDEs) serve as a vital mechanism to hydrolyze cyclic nucleotides and turn off neurohormonal stimulation. PDE-mediated hydrolysis contributes to the discrete cyclic nucleotide signaling known as the cAMP and cGMP nanodomains. PDEs also define the amplitude and duration of local cyclic nucleotide signaling to regulate a broad range of effectors, including protein kinases, ion channels, and exchange factors. Through local actions of downstream effectors, PDEs regulate specific physiological responses in a cell- and tissue-specific manner. The ubiquitous PDE activity in the heart and vessels has been demonstrated since the discovery of the first enzymatic deconstruction of cAMP into AMP in the 1950s (1–3). Thus, PDEs critically regulate physiological responses in the cardiovascular system, including cardiac inotropic and chronotropic responses, vessel dilation and constriction, and metabolism. Over the past 30 years, there has been significant progress in understanding this family of enzymes in physiology and cardiovascular diseases (4–10). Abnormal PDE expression and activities are involved in structural and functional remodeling in the heart and vessels. Disrupted cyclic nucleotide signals contribute to heart failure (HF), arrhythmia, hypertension, aneurysm, atherosclerosis, and other cardiovascular diseases. PDE inhibitors have been successfully used in clinical settings and present an intensive area of research. This review summarizes the advances in PDE regulation in the heart and vessels and the implications for targeting these enzymes in the therapy of cardiovascular diseases.

## 2. OVERVIEW OF THE PDE SUPERFAMILY

**FIGURE 1.**
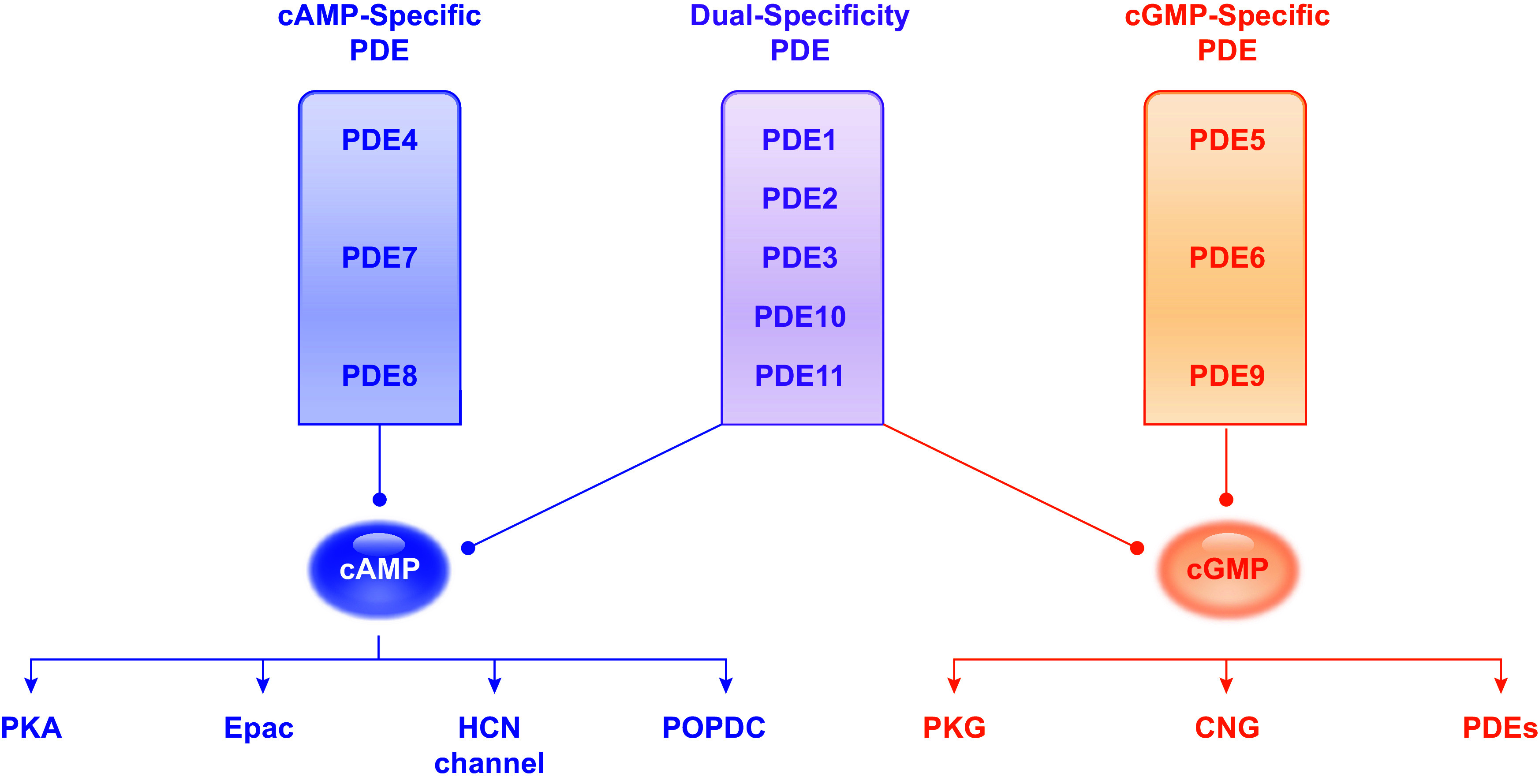
Classification and specificity of phosphodiesterases (PDEs) toward cyclic nucleotides and downstream effectors. PDE4, 7, and 8 hydrolyze cAMP, whereas PDE5, 6, and 9 hydrolyze cGMP. The other PDEs, including PDE1, 2, 3, 10, and 11, hydrolyze both cAMP and cGMP. cAMP targets protein kinase A (PKA), exchange protein directly activated by cAMP (Epac), hyperpolarization-activated cyclic nucleotide-gated channel (HCN), and popeye domain containing protein (POPDC), whereas cGMP targets protein kinase G (PKG), cyclic nucleotide-gated channel (CNG), and PDE.

The PDE superfamily has 11 subfamilies (PDE1–11) distinguished by their unique structure, enzymatic properties, regulation, and pharmacology (11). Each PDE comprises a catalytic domain, a carboxy terminus, and an amino terminus (FIGURE 1). All PDEs share a similar structure with the catalytic domains at the carboxy-terminal regions. Within the PDE isoforms encoded by the same gene, the distinct amino termini define the subcellular location of individual isoforms, while they share identical catalytic and carboxy-terminal domains (7, 12). PDE isoforms are also subjected to unique enzymatic regulation by posttranslation modification and regulatory proteins. There are 21 PDE genes expressed in mammalian cells, including single PDE2A, PDE5A, PDE9A, PDE10A, and PDE11A genes, two PDE3 (PDE3A and 3B), PDE7 (PDE7A and 7B), and PDE8 (PDE8A and 8B) genes, three PDE1 (PDE1A, 1B, and 1C) and PDE6 (PDE6A, 6B, and 6C) genes, and four PDE4 (PDE4A, 4B, 4C, and 4D) genes (4–10). Over 100 different isoforms are expressed in mammalian cells because of alternative splicing and different translation initiation sites. Based on substrate specificity, these enzymes are classified as cAMP-specific, cGMP-specific, and dual-substrate PDEs. PDE4, 7, and 8 are exclusively involved in cAMP hydrolysis, whereas PDE5, 6, and 9 have cGMP-specific enzymatic activities. The remaining PDEs, including PDE1, 2, 3, and the latter members PDE10 and 11, can hydrolyze both cAMP and cGMP. PDE2, PDE3, and, to a lesser extent, PDE1 have been shown to mediate cGMP-cAMP cross talk upon activation or inhibition of the PDE activities via cGMP binding. cGMP competitively inhibits the cAMP hydrolytic activity of PDE1 and PDE3 (7). In contrast, PDE2 is the only member that is activated upon allosteric cGMP binding, increasing its cAMP hydrolytic activity (13).

### 2.1. The Expression, Biochemical Property, and Regulation of PDEs in the Cardiovascular System

**FIGURE 2.**
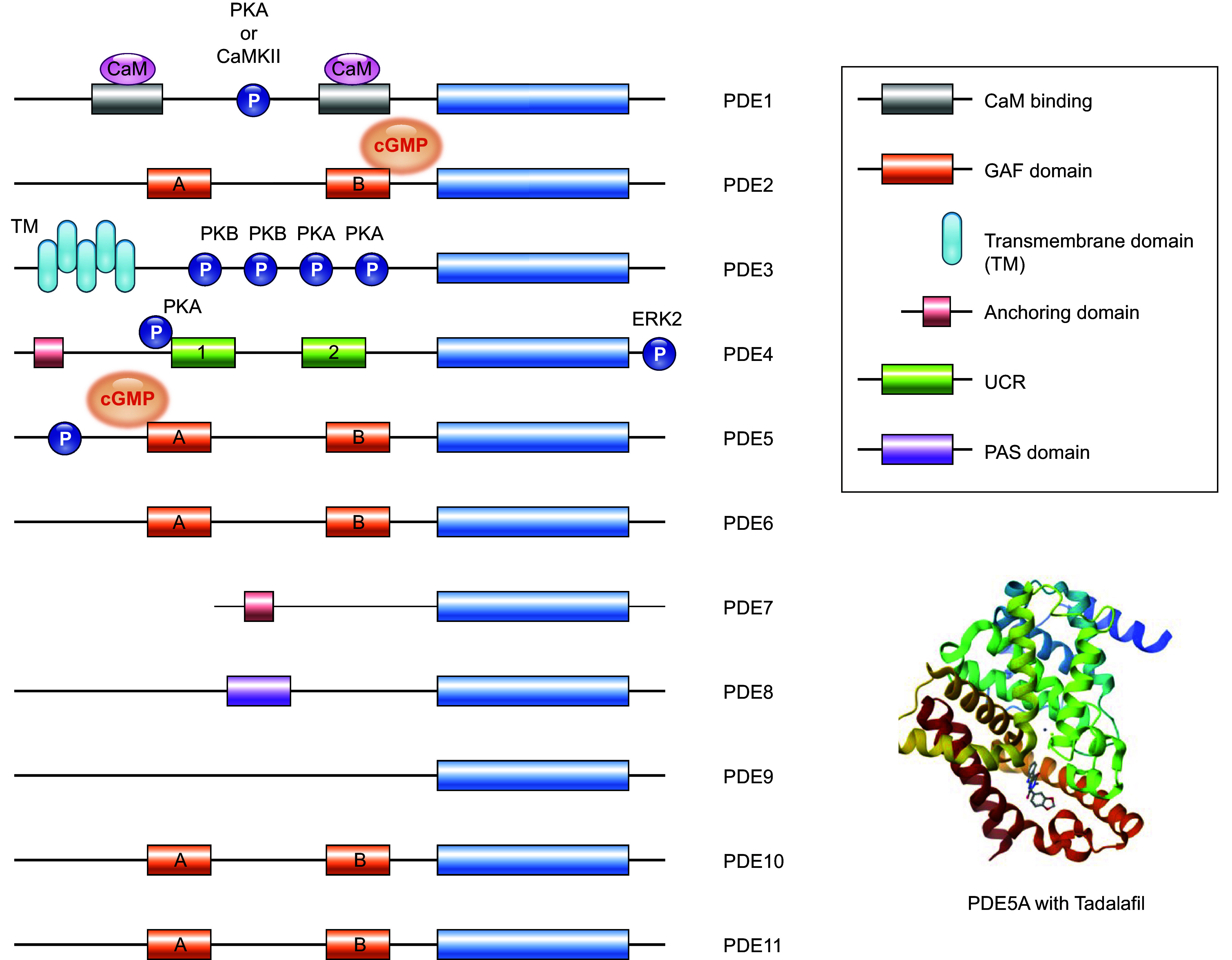
Structure and regulation of phosphodiesterases (PDEs). The catalytic domains conserved in the carboxy terminal of PDEs are depicted as blue cylinders. These enzymes can be precisely regulated by specific targeting domains and modifications, such as phosphorylation, which enables subcellular localization and activity of PDEs. Certain PDEs can dimerize; long isoforms of PDE4, for example, dimerize because of the presence of 2 upstream conserved regions (UCR1 and UCR2). Other PDEs such as PDE2 and PDE5 form a dimer by means of their GAF [cyclic guanylate monophosphate (cGMP)-dependent phosphodiesterase, *Anabaena* adenylyl cyclase, *Escherichia coli* FhlA] domains. PDEs with GAF domains, such as PDE2, PDE5, PDE10A and PDE11, are regulated by cyclic nucleotides, with cGMP activating PDE2 and cAMP inhibiting PDE10. The structure of PDE5A with tadalafil is shown at *bottom right*. CaM, calmodulin; CaMKII, Ca^2+^/CaM-dependent kinase II; ERK, extracellular signaling-related kinase; PAS, Per-Arnt-Sim; PKA, protein kinase A; PKB, protein kinase B; TM, transmembrane domain.

**FIGURE 3.**
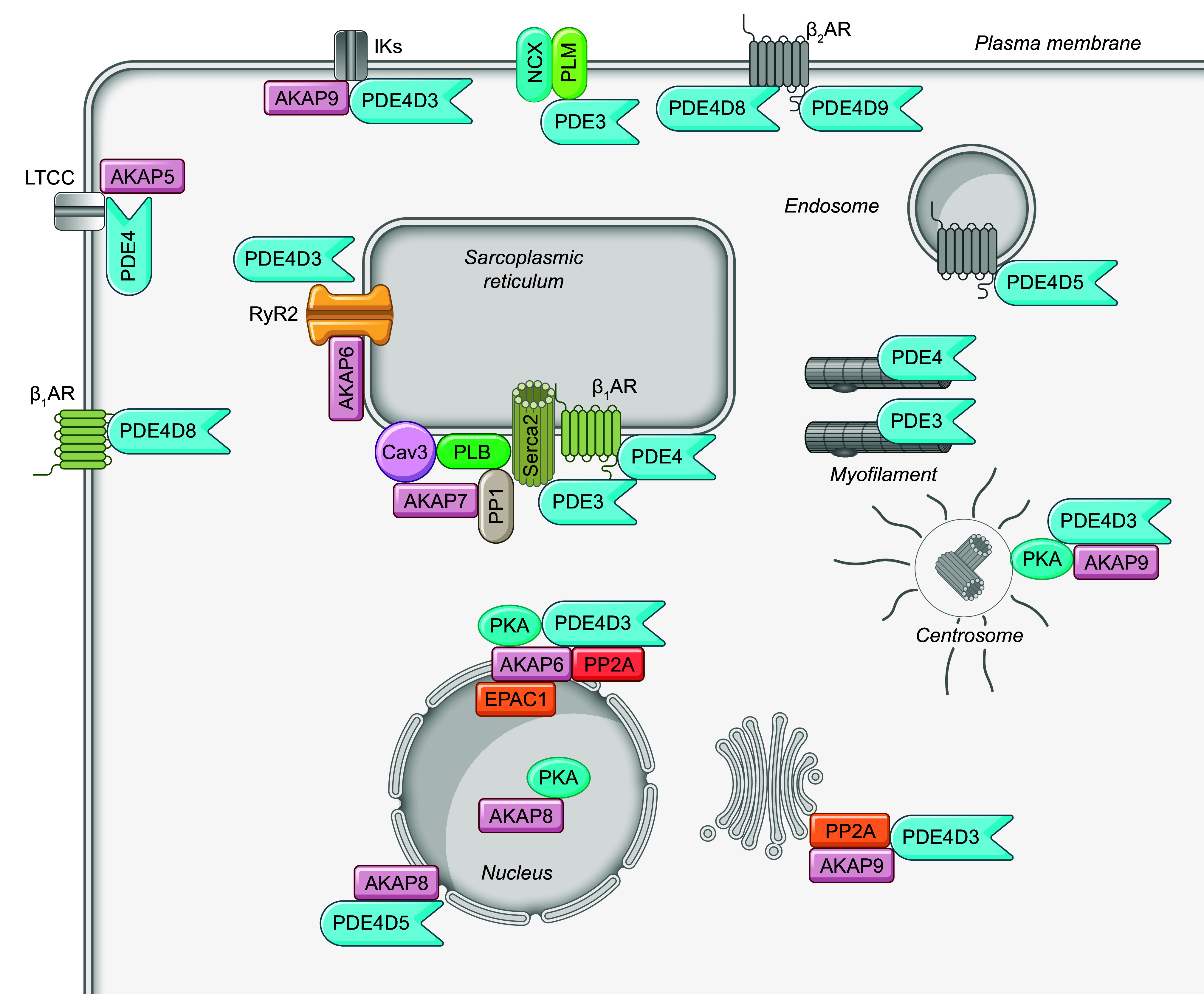
Subcellular localization of phosphodiesterase (PDE) signalosomes in cardiomyocytes. PDEs form signalosomes with various protein partners, such as protein kinase A (PKA), PKA anchoring proteins, and receptors, which are localized in distinct subcellular compartments of cardiomyocytes. AKAP, A-kinase anchoring protein; β_1_AR, β_1_ adrenergic receptor; β_2_AR, β_2_ adrenergic receptor; Cav3, caveolin 3; Epac, exchange protein activated by cAMP; ERK, extracellular signaling-related kinase; IK, calcium-activated potassium channel; LTCC, L-type calcium channel; NCX, sodium/calcium exchanger; PKG, protein kinase G; PLB, phospholamban; PP1, protein phosphatase 1; PP2A, protein phosphatase 2A; RyR2, ryanodine receptor 2; SERCA, sarco(endo)plasmic reticulum calcium ATPase.

**FIGURE 4.**
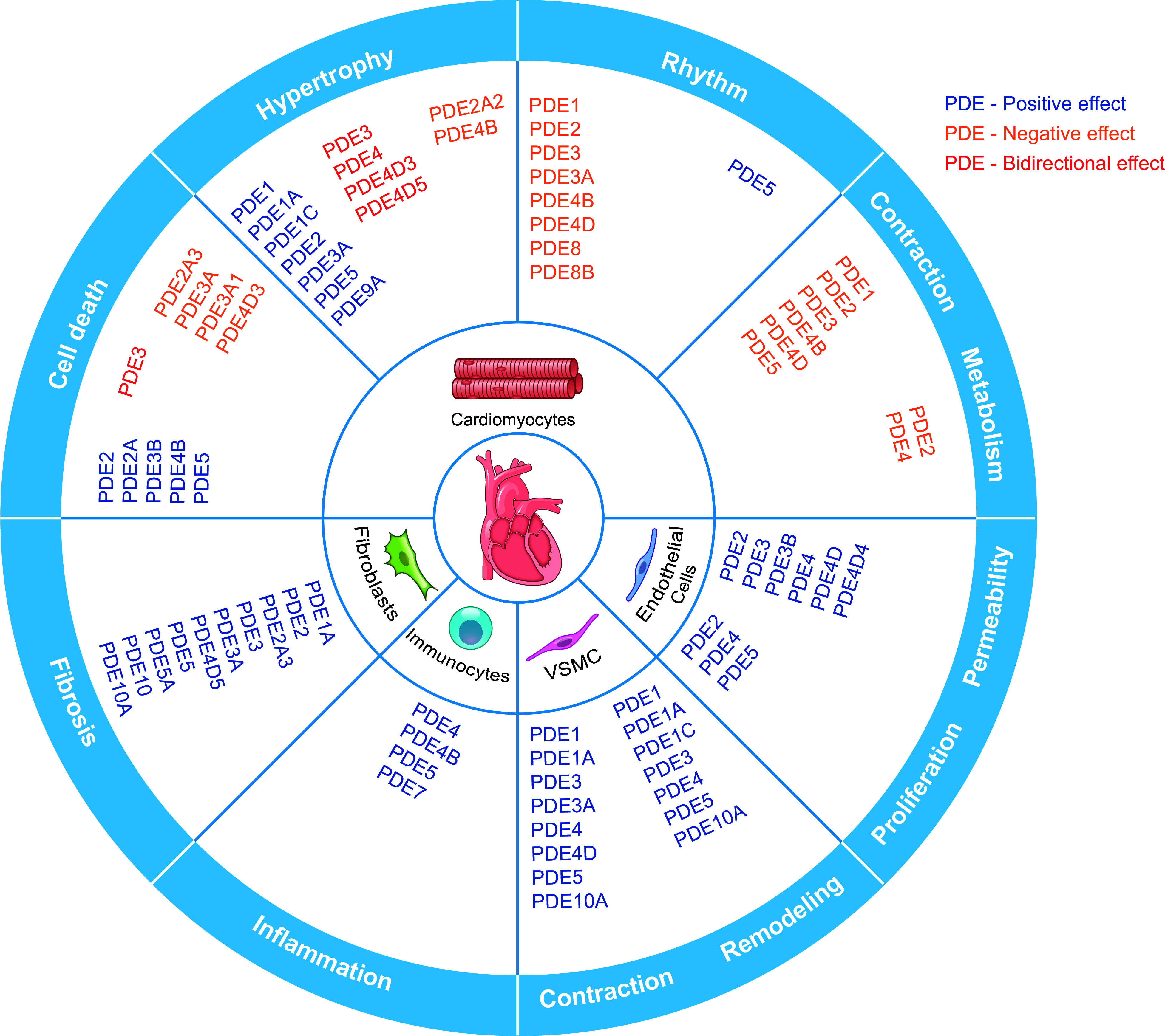
The expression and function of phosphodiesterases (PDEs) in the cardiovascular system. The cartoon highlights the expression of individual isoforms in different cardiovascular cells. The colors indicate the positive, negative, or bidirectional roles of PDE isoforms in specific function in the heart and vessels. VSMC, vascular smooth muscle cell.

PDEs are expressed in a cell- and tissue-specific manner, with only a few enzymes expressed in any single cell type. Except for PDE6, all PDE families have a broad expression in different tissues, including hearts and vessels (7, 12). Many have different expression and activity in the heart between rodents and humans, such as PDE1, 3, and 4 (14). Of the PDE superfamily, PDE1, 2, 3, 4, 5, 8, and 9 are fundamental components of the cardiac signaling and function regulation under physiological and pathophysiological conditions (8). On the other hand, PDE1, 2, 3, 4, 5, and 7 constitute significant PDE activity in the vasculature (15, 16). PDE1, 2, 3, 4, and 5 are extensively studied in the cardiovascular system (4–9, 16). (FIGURES 2–4 and TABLE 1).

**Table 1.** The substrates, biochemical properties and subcellular distribution of individual PDE families and isoforms

PDE Family	Isoform	Variant	Substrate	Regulation	Localization
PDE1				CaM-stimulated PDE	
	PDE1A		cGMP>cAMP, cGMP (in vivo)	PKA	
	PDE1B		cGMP>cAMP, cGMP (in vivo)	Ca^2+^/calmodulin	
	PDE1C		cAMP/cGMP, cAMP (in vivo)	PKA	Cytoplasm
PDE2				cGMP-stimulated PDE	
	PDE2A				
		PDE2A1	cAMP/cGMP		Cytosol, soluble in the cytoplasm
		PDE2A2	cAMP/cGMP		Mitochondria
		PDE2A3	cAMP/cGMP		Golgi body, plasma membrane
PDE3				cGMP-inhibited PDE	
	PDE3A				
		PDE3A1	cAMP>cGMP	PKA	Cytoplasm, particulate fraction microsomal and cytosol
		PDE3A2	cAMP>cGMP	PKC, PKA	Cytosolic and particulate fractions
		PDE3A3	cAMP>cGMP		Cytoplasm
	PDE3B		cAMP>cGMP	PKA	T tubules (proximity to mitochondria)
PDE4				cAMP-specific PDE	
	PDE4A		cAMP	PKA	Membranes
	PDE4B		cAMP	PKA, ERK	Membranes
	PDE4C				
	PDE4D		cAMP	PKA, CaMKII	Lipid rafts and caveolar membrane, myofilaments, nuclear envelope
		PDE4D3			RYR2, nuclear envelope
		PDE4D5			Nuclear envelope
		PDE4D8			
		PDE4D9			
PDE5				cGMP-specific PDE	
	PDE5A1–3		cGMP		Cytosol, Z lines, caveolae (endothelial cell)
PDE6				cGMP-specific PDE	
	PDE6D/H				
	PDE6γ				
PDE7				cAMP-specific PDE	
	PDE7A		cAMP	PKA	Cytoplasm
		PDE7A2			
	PDE7B				
PDE8					
	PDE8A		cAMP	PKA	
	PDE8B				
PDE9	PDE9A		cGMP		
PDE10	PDE10A2		cAMP, cGMP		
PDE11	PDE11A		cAMP, cGMP		

CaM, calmodulin; CaMKII, Ca^2+^/CaM-dependent kinase II; cAMP, cyclic adenosine monophosphate, cGMP, cyclic guanosine monophosphate; ERK, extracellular signal-regulated kinase; PDE, phosphodiesterase; PKA, protein kinase A, PKC, protein kinase C.

#### 2.1.1. PDE1.

PDE1 constitutes a family of Ca^2+^/calmodulin (CaM)-dependent enzymes, with all three PDE1 isozymes (PDE1A, PDE1B, and PDE1C) expressed in the heart and vessels (17). PDE1A is expressed in humans (18), bovines (19), canines (20), and rats (21), including both cardiomyocytes (22, 23) and cardiac fibroblasts (24, 25). PDE1C is primarily restricted to cardiomyocytes (19) and is the predominant PDE1 isoform in rabbit, dog, and human hearts (26). The messenger RNA (mRNA) expression levels of PDE1A are sex dependent, with higher levels in males compared with females (27). PDE1 functions as a dual esterase that hydrolyzes cAMP and cGMP, with PDE1A and PDE1B displaying a lower affinity for cAMP, hence favoring cGMP hydrolysis (18, 28). Several studies have demonstrated that PDE1A and PDE1B primarily regulate cGMP in vivo (12, 23, 29). On the other hand, PDE1C hydrolyzes cAMP and cGMP with comparably high affinity and low Michaelis constant (*K*_m_) values. Whereas PDE1C has been shown to regulate intracellular cAMP levels in various cell types, a role in cGMP regulation has not been described in vivo (30–32). PDE1 isozymes contain two CaM-binding domains at the amino termini, two phosphorylation sites, and an inhibitory region that keeps the enzymes in an inactive configuration when intracellular Ca^2+^ is low (33). The binding of CaM to PDE1 increases the activity up to 10-fold in vitro (33). Thus, PDE1 isozymes are important in the cross talk between second messenger Ca^2+^ and cyclic nucleotide signaling (34). Phosphorylation of PDE1 by either protein kinase A (PKA for PDE1A and PDE1C) (35, 36) or Ca^2+^/CaM-dependent protein kinase II (CaMKII for PDE1B) (37) reduces the affinity for Ca^2+^ and CaM, thereby limiting enzymatic activity. Conversely, the binding of CaM to PDE1 elevates hydrolytic activity by preventing PKA- and CaMKII-mediated phosphorylation and promoting a conformational change that raises the maximal catalytic activity (38, 39).

#### 2.1.2. PDE2.

A single PDE2 gene, PDE2A, is expressed with three alternative splicing isoforms (40). PDE2 has been found in human, rat, and mouse hearts (41–43). PDE2A has been detected in both cardiomyocytes and cardiac fibroblasts, with higher expression in the latter (41, 44). The PDE2A isoforms display different subcellular locations, with PDE2A1 being soluble in the cytoplasm and PDE2A2 and PDE2A3 predominantly in a particulate fraction (40). Notably, PDE2A3 is localized in the Golgi (45, 46), whereas PDE2A2 is detected in the mitochondria and regulates local mitochondria-related cAMP pools (47, 48). In addition to cardiomyocytes, PDE2A has been found in neonatal cardiac fibroblasts (44) and endothelial cells (ECs) (49–51). PDE2 shares similarities with PDE1, capable of hydrolyzing both cAMP and cGMP, displaying comparable maximal rates and low *K*_m_ values for each cyclic nucleotide but with a slight preference for cGMP (52, 53). The amino terminus contains two cGMP-stimulated PDE, *Anabaena* AC, and Fhla transcription factor (GAF) domains, designated as GAF-A and GAF-B (54). The GAF-B domain selectively binds to cGMP, altering the conformation of PDE2 and raising its esterase activity by a factor of 30 (55, 56). PDE2 is thus uniquely designated as the cGMP-stimulated PDE. By contrast, it is unlikely that cAMP regulates PDE2 activity in vivo in the same manner, given that the affinity of cAMP for the GAF-B site is ∼100-fold lower than that of cGMP (57).

#### 2.1.3. PDE3.

PDE3, the third dual esterase, is a key player in hydrolyzing cAMP and cGMP with high affinities. PDE3 is highly expressed in hearts from small rodents to large mammals, including humans, and constitutes the major cAMP-hydrolyzing activity in the latter (58, 59). PDE3 activity is present in cytosolic and particulate fractions (60). PDE3 enzymes are encoded by two genes, PDE3A and PDE3B. PDE3A is the predominant gene expressed in the heart (61, 62) and is responsible for the tonic effects in the myocardium (63, 64). Three splicing isoforms expressed by PDE3A possess different amino-terminal domains that dictate their subcellular locations. The longest isoform, PDE3A1, is concentrated in particulate fraction and contains two amino-terminal hydrophobic domains (NHRs), NHR1 and NHR2. The short isoform, PDE3A3, lacks both domains and resides in the cytoplasm (62). In comparison, the mRNA expression levels of PDE3B vary depending on sex, with lower levels in males than in females (27). Only one PDE3B isoform is identified in the T tubules near the mitochondria (59, 65). PDE3 enzymes hydrolyze cAMP and cGMP with high affinity, but the *V*_max_ for cGMP is 10 times lower than that for cAMP (53). When cGMP binds to the catalytic site of PDE3, it is hydrolyzed very slowly, inhibiting its enzymatic activity toward cAMP metabolism. Hence, PDE3 is often referred to as a cGMP-inhibited PDE. As a result, PDE3 regulates the intricate cGMP-cAMP cross talk, with the binding of cGMP inhibiting PDE3 activity and acting as a positive regulator of cAMP signaling (66, 67). Biochemically, two isoforms of PDE3A, PDE3A1 and PDE3A2, can be phosphorylated at the common amino-terminal sequence. PDE3A1 is subjected to PKA-dependent phosphorylation of Ser 312, and PKC preferentially phosphorylates PDE3A2 at Ser 428. Both phosphorylation events promote the association of PDE3A isoforms with cellular signaling proteins, such as 14-3-3 (22, 60). PKA also phosphorylates PDE3A1 at Ser 292 of the amino terminus, enhancing its hydrolytic activity and association with multiple proteins, like A-kinase anchoring protein 18 (AKAP18), sarco(endo)plasmic reticulum calcium ATPase 2a (SERCA2a), protein phosphatase 2A (PP2A), PP1, and caveolin 3 (60). Mutation of PDE3A1 at Ser 292 remarkably abolishes the PKA-dependent interaction with SERCA2a, whereas Ser 312 and Ser 428 mutation slightly reduced that association (60). Similarly, a PKA-phosphorylated PDE3B at Ser 318 increases its catalytic activity and interaction with 14-3-3 (68).

#### 2.1.4. PDE4.

PDE4 enzymes are highly selective for cAMP, evidenced by their very low *K*_m_ values for this cyclic nucleotide (69, 70). In rodent hearts, PDE4 is the dominant family for cAMP-hydrolyzing activities, together with PDE3, accounting for ∼90% of overall cAMP-hydrolyzing activities (14). However, in human hearts, PDE3- and PDE4-mediated cAMP-hydrolyzing activity accounts for ∼40% of total cAMP-hydrolyzing activities, with other non-PDE4 such as PDE1 being much more active (14). Three PDE4 genes are reported in human and rodent hearts, namely PDE4A, PDE4B, and PDE4D (14, 71, 72). PDE4D has higher expression in female than male mouse hearts (73), whereas ovariectomy (OVX) promotes the expression of PDE4B mRNA in rat hearts (74). Both may contribute to the sex-dependent difference in excitation-contraction coupling (ECC) (75). PDE4 provides an important feedback mechanism to control cAMP levels in various cell types, including cardiomyocytes (76–78), smooth muscle cells (SMCs) (79, 80), ECs (27, 81), and fibroblasts (82, 83). Alternative gene splicing produces >20 PDE4 isoforms (PDE4A1–PDE4A8, PDE4B1–PDE4B6, and PDE4D1–PDE4D11), which can exist in short and long forms depending on the presence of upstream conserved regions (UCRs) within the amino terminus (84). Of the nine PDE4D isoforms, at least four PDE4D variants have been detected in human and rodent hearts, including long isoforms PDE4D3, 4D5, 4D8, and 4D9 (14). Whereas the long versions contain UCR1 and UCR2, short PDE4 isoforms possess a single UCR2 domain, and supershort isoforms have none. UCR1 and UCR2 are required for dimerization (85, 86), whereas short and supershort forms without UCR1 remain monomers. PDE4 possesses a PKA phosphorylation site within UCR1, which elevates esterase activity by precluding UCR1-UCR2 interactions (87). CaMKII phosphorylation has been shown to increase the catalytic activity of PDE4D and control cAMP levels in cardiomyocytes, indicating that PDE4D underlies cross talk between Ca^2+^ and cAMP signaling (88). Additionally, an extracellular signal-regulated kinase (ERK) phosphorylation site at the carboxy terminus serves as a negative regulation of all isoforms (89).

#### 2.1.5. PDE5.

PDE5 is selective for cGMP hydrolysis. In humans, three isoforms of PDE5A, PDE5A1, A2, and A3, are expressed but with no difference in reported signaling and function (22, 90–92). PDE5 is detected in cardiomyocytes (93–95) despite early negative reports on the PDE5 expression in the heart (96–98). PDE5 localizes to the cytoplasm and the Z lines of cardiomyocytes and controls a pool of cGMP produced by soluble GC (sGC), whereas PDE2 and PDE9 regulate cGMP produced by the particulate GC activity of NPRs (91, 99). PDE5 is also present in cardiac fibroblasts and has been shown to participate in fibroblast transformation and proliferation (97, 100). In vascular ECs, PDE5 is in caveolae and negatively modulates nitric oxide synthase 3 (NOS3) signaling (101). PDE5 is also abundantly expressed in vascular SMCs, where it plays a central role in regulating vascular tone (96, 102). PDE5 contains GAF-A and GAF-B domains within its amino terminus. The binding of cGMP to the GAF-A domain enhances the allosteric activation of PDE5 (103, 104), whereas GAF-B contributes to the dimerization of the enzyme (105). Phosphorylation of PDE5 by PKA and protein kinase G (PKG) amplifies the cGMP affinity of the GAF-A domain, thereby stabilizing the active enzyme conformation and maintaining hydrolytic activity (106–108). Thus, cGMP fosters its own degradation through negative feedback.

#### 2.1.6. PDE6–11.

PDE6 is a cGMP-specific enzyme primarily expressed in retinal rod and cone cells and is not present in the heart (109). Recently, PDE6 isoforms have been identified outside the eye, especially PDE6γ in mouse lungs (110), which plays a critical role in regulating p42/p44 mitogen-activated protein kinase (MAPK) signaling. PDE6γ has been observed in rat pulmonary vessels and in human pulmonary SMCs (111). These findings suggest that certain PDE6 subtypes could be therapeutic targets in pulmonary hypertension.

PDE7 comprises two genes, PDE7A and PDE7B, both detectable within the heart (112–114). PDE7 contains an amino-terminal sequence with a PKA phosphorylation site, a catalytic domain with two ion (Zn^2+^ and Mg^2+^)-binding sites, and a carboxy-terminal sequence (115). PDE7A2 is present in skeletal and cardiac muscles; however, its cardiac function has not been well defined.

PDE8 comprises two genes, PDE8A and 8B, which are present in human and mouse hearts as well as mouse cardiomyocytes (116–118). PDE8 has at least nine splice variants insensitive to the nonselective PDE inhibitor 3-isobutyl-1-methylxanthine (IBMX) (119). PDE8 contains a receiver (REC) and a Per, Arnt, and Sim (PAS) domain at the amino terminus involved in protein-protein interactions. PDE8A is activated by PKA phosphorylation (120).

PDE9 is encoded by a single gene, PDE9A, which has >20 variants (121, 122). PDE9 has a remarkably high affinity for cGMP, with a *K*_m_ of 170 nM, much lower than the *K*_m_ of 230 µM for cAMP. PDE9 thus is emerging as an essential regulator of cGMP signaling (121). The enzyme is about twice as active in the presence of 1–10 mM Mn^2+^ than in the presence of the same concentration of Mg^2+^ or Ca^2+^. PDE9A is insensitive (up to 100 µM) to various PDE inhibitors, including rolipram, vinpocetine, SKF-94120, dipyridamole, and IBMX, but is inhibited by zaprinast (IC_50_ = 35 µM), a PDE5 inhibitor. PDE9A lacks a region homologous to the allosteric cGMP-binding domains found in other cGMP-binding PDEs. Myocardial PDE9 preferentially hydrolyzes natriuretic peptide (NP)-mediated increases in cGMP but minimally affects the nitric oxide (NO)-soluble GC (sGC)-generated cGMP (123). PDE9 is also expressed in SMCs, where it metabolizes NO-sGC-generated cGMP, in contrast to its role in the myocardium (124).

PDE10, like PDE1, 2, and 3, hydrolyzes both cAMP and cGMP. PDE10 contains two amino-terminal GAF domains and a PKA phosphorylation site (125). cAMP binding to the catalytic domain of PDE10 inhibits its cGMP hydrolysis (126, 127); however, cAMP binding to the GAF domain activates PDE10. Thus, cAMP can have biphasic effects on cGMP hydrolysis depending on its concentration, in which low levels of cAMP binding to the GAF domain increase PDE10 activity for cGMP hydrolysis and high levels of cAMP decrease PDE10 activity for cGMP hydrolysis (128). PDE10A2 is the major PDE10A isoform expressed in the heart (129). Whereas PDE10 expression is low in healthy hearts, it is upregulated in mouse and human failing hearts (129).

PDE 11A is a dual esterase hydrolyzing both cAMP and cGMP and has four splice variants (130, 131). PDE11A mRNA expression has been detected in the heart (130, 132). Whereas the PDE11A protein is not detected in blood vessels or cardiomyocytes, weak expression has been found in neuronal cells within parasympathetic ganglia in the heart (132). At the amino terminus, PDE11A1 contains a single GAF domain, which is similar to those found in other PDEs. This domain constitutes a potential allosteric binding site for cGMP or another small ligand (130). PDE11A1 hydrolyzes both cGMP and cAMP with similar *V*_max_ values but with *K*_m_ values of 0.52 μM and 1.04 μM, respectively (130). Although PDE11A can be potently inhibited by the PDE5 inhibitor tadalafil, its function in the cardiovascular system remains unknown (133).

### 2.2. Subcellular-Localized Specific PDE-Containing Signalosomes and Cyclic Nucleotide Nanodomains

Localizing PDE enzymes within signalosomes and nanodomains is a fundamental mechanism for regulating cAMP and cGMP signaling in cells. Hormones and neurotransmitters initiate intracellular signaling by activating membrane receptors and using second messengers such as cAMP and cGMP. cAMP targets two primary downstream effectors, PKA, including type 1 and 2 PKA (134), and exchange protein directly activated by cAMP (Epac) (135), to modulate cellular responses. cGMP primarily activates PKG1 and PKG2 to enhance downstream substrate phosphorylation (136). Additionally, cAMP and cGMP can directly bind to and activate ion channels at the plasma membrane (PM) (137, 138). Sutherland raised a model that synthesizes cAMP from the PM and diffuses it throughout the cell (139). However, such a model fails to explain the hormone-specific cellular effects in response to cyclic nucleotides. For instance, stimulation of β-adrenoceptor (βAR) but not prostaglandin EP4 receptor triggers inotropic effects in cardiomyocytes even though both GPCRs induce similar levels of cAMP signals and PKA activities (140, 141). Despite being ubiquitous messengers diffusible inside cells, experimental (6–8) and computational studies (142–146) support that PDEs compartmentalize the cyclic nucleotide signals into nanodomains in a cell to achieve specific cellular responses. The precise mechanisms by which PDEs accomplish this remain an active area of investigation (143, 146). In this paradigm, the ubiquitous second messengers are responsible for diverse downstream effects and cellular responses at distinct subcellular compartments, which results in precise physiological stimulation in a cell- and tissue-specific manner. Hence, subcellular-localized PDEs control the amplitude, duration, and localization of cAMP and cGMP, including spatiotemporally hydrolyzing these cyclic nucleotides, preventing the diffusion of the cyclic nucleotide, and creating the discrete cyclic nucleotide nanodomains in a cell (10). Disruption of PDE subcellular localization contributes to pathophysiological processes, including hypertension and HF (4–10). Here, we summarize cyclic nucleotide nanodomains in subcellular compartments.

PDEs are distributed within distinct subcellular compartments, granting precise control of cyclic nucleotide and subsequent protein kinase signaling. For example, PDE4 isoforms are enriched in membranes, facilitating receptor signaling at the PM (147–149). Similarly, PDE4D isoforms are localized within lipid rafts and caveolar membranes and control local signaling at the dyad region in the transverse (T)-tubular membrane and the sarcoplasmic reticulum (SR) (150–153). PDE4D is also localized to the myofilaments (152) and the nuclear envelope (46, 154–156). PDE3A1 contains a unique amino-terminal extension of hydrophobic loops embedded in intracellular membranes and associated with SERCA2a and AKAP18. PDE3A2 presents only in the cytoplasm, whereas PDE3A3 exists at both cytoplasm and intracellular membrane locations. PDE2 isoforms are distributed in the cytoplasm (PDE2A1), at the PM (PDE2A3), and in the mitochondria (PDE2A2) (40, 157). PDE5 is localized at the Z band (158). In isolated rat cardiomyocytes, NO promotes the synthesis of a cytoplasmic pool of cGMP that is hydrolyzed specifically by PDE5, whereas NPs generate a separate juxtamembrane cGMP pool that is regulated by PDE2 (99). Functional β_3_ARs are localized exclusively within the T-tubular membrane and stimulate a cGMP pool that is predominantly regulated by PDE2 and PDE5 (159). Additionally, PDE2 confines the membrane-associated pool of cGMP generated via NP-GC-A signaling within the region of the T tubules in isolated cardiomyocytes (160). Furthermore, PDE1A is expressed in the cytoplasm in contractile SMCs but shifted to the nucleus in pathological proliferative SMCs (29). The general idea is that strategically located PDEs in subcellular compartments dictate the spatiotemporal pattern of cAMP and cGMP in a cell (7), which forms distinct nanodomains by specific intracellular location and proximity with a limited subset of effectors in the local vicinity.

Moreover, PDEs are assembled into isoform-specific macromolecular signalosomes within distinct subcellular nanodomains, granting a precise control of cyclic nucleotide and subsequent protein kinase signaling (FIGURE 3). The PDE supramolecular signalosomes are organized by anchoring proteins, including AKAPs and membrane-associated guanylate kinases (MAGUKs), which can be remodeled in pathogenesis. The AKAP family comprises >50 proteins, known for their ability to bring together PKA and the cAMP-termination PDEs into distinct subcellular locations. Although PKA is a ubiquitous enzyme that phosphorylates a broad range of substrates, the association of PKA with AKAP facilitates PKA phosphorylation of local substrates in the vicinity. AKAPs interact with many other signaling molecules, including receptors, PDEs, kinases, and PPs, and downstream targets such as ion channels and myofilaments. These complexes position all the signaling molecules at the regions of cAMP production. The PDEs in the complexes physically limit cAMP access and restrict phosphorylation of a subset of substrates at specific subcellular locations, such as PM, junctional SR, and myofilament, etc. At the PM, various PDE isoforms have been shown to couple with hormone receptors, for example, βARs (148, 161–166), prostaglandin EP4 receptors (141, 148), adenosine A2R (167), and NPRs (99, 123, 160, 168). Without affecting the global cAMP level, PDE4D deletion increases the localized cAMP response to β-stimulation by more than twofold (169). PDEs also associate with ion channels (170, 171) and the Na^+^-K^+^-transporting ATPase (172). PDE3 and PDE4 control Ca^2+^ release and reuptake in the SR by ryanodine receptor 2 (RyR2) and SERCA2a, respectively (46, 63, 169, 173). Conversely, AKAP79 (147), AKAP150 (174), AKAP5 (175), and AKAP9 (171) are distributed at the PM and proximate with various membrane proteins, including βARs, L-type calcium channel (LTCC), and potassium channel (KCNQ2). AKAP250 (AKAP12) associates with β_2_AR and PDE4 at the peripheral actin cytoskeleton near the PM (149). AKAP6, also known as mAKAP, has been shown to colocalize with PDE4D3, PKA, PP2A, exchange protein directly activated by cAMP 1 (EPAC1), and ERK at the cardiomyocyte nuclear envelope (155, 156). On the other hand, AKAP6 and AKAP7 have been identified to interact with either RyR2 or SERCA2a at junctional or nonjunctional SR, respectively (176, 177). AKAP350 (also named Yatiao, AKAP450, AKAP9) tethers PDE4D3 and PP2A with signaling molecules, including protein kinase C (PKC), protein kinase N (PKN), and casein kinase-1, at the junctional region, centrosomes, or Golgi (175). AKAP95 (AKAP8) targets PDE4D and PKA in the nuclear matrix (178). In comparison, although PKG is also believed to be anchored at the subcellular complexes via its amino-terminal leucine zipper domain in a cell, only a few anchoring proteins have been identified for PKG (179). These signalosomes affirm the significance of PDE-mediated hydrolysis in the regulation of discrete cyclic nucleotide signaling, known as cAMP and cGMP nanodomains.

### 2.3. Detection of Subcellular-Localized Specific PDE-Controlled Cyclic Nucleotide Nanodomains

Our comprehension of compartmentalized cAMP distribution within a cell has been validated with visualization techniques such as Förster resonance energy transfer (FRET)-based biosensors (146). The nanometer-scale resolution of FRET imaging demonstrates the heterogeneous distribution of cAMP within subcellular compartments (180–185). The distinctive membrane architecture and geometry of cardiomyocytes provide a framework for specific cAMP signaling domains. A typical human cardiomyocyte is 100 µm long and 10–25 µm in diameter, with abundant lipid rafts and caveolae constituting various local vicinities for signaling domains. Furthermore, cardiomyocytes present extensive T tubules resulting from deep invagination and extension of the PM. T tubules are at the proximity to the SR networks, the calcium storage, and the myofilament. The maximum distance between the PM, the SR, and the myofilament is ∼300 nM (184). Notably, most FRET-based studies have been performed in cardiomyocytes, and information regarding cyclic nucleotide signaling nanodomains in other cell types, such as SMCs, remains scarce.

FRET-based biosensors have significantly enhanced our understanding of cyclic nucleotide signaling regulation and cAMP-dependent protein kinase activity at the nanoscale (180–183). The spatial confinement of cAMP nanodomains is disrupted by the inhibition of PDEs, indicating the key role of these enzymes in cAMP compartmentalization (10). Recently, the cAMP sensors have been genetically modified to target them to different subcellular compartments and monitor cAMP signaling events at specific sites (141, 152, 184, 186–192). These targeted tools have uncovered novel complexity and interconnectivity that regulate cAMP subcellular domains and reveal the functional relevance of individual cAMP pools. One emerging essential point is that the radius of individual cAMP domains can be as small as a few tens of nanometers (185). Various cAMP nanodomains controlled by highly localized signalosomes have been uncovered in cardiomyocytes. For example, these sensors reveal that the PM, SR, and myofilaments experience distinct amplitudes and kinetics of cAMP signals in response to catecholamine stimulation, even if they are <300 nm apart (184). Similarly, the β-agonist isoproterenol (ISO) triggers a higher and more sensitive PKA response at the PM than at SR and myofilaments, measured by subcellular anchored PKA biosensors (152). These discrete cAMP pools rely on PDE-mediated cAMP degradation. Inhibition of PDE by IBMX normalizes all the cAMP responses at different subcellular sites (184). Intriguingly, the homogeneous cAMP signals induced by the AC agonist forskolin in the presence of IBMX generate a higher troponin I phosphorylation but compromise contractile response relative to β-adrenergic stimulation, suggesting that the compartmentalization of cAMP is essential for the optimal inotropic response in cardiomyocytes (152).

In comparison, the local distribution of cGMP remains less investigated. One of the possible reasons is that cGMP content is 10-fold lower relative to cAMP, which poses a significant challenge for detecting subtle cGMP signals with traditional experimental approaches such as enzymatic quantification. Although the FRET-based biosensors have been developed to detect cGMP, these sensors either are not specific or have lower affinities, making them ineffective for detecting cGMP in cardiomyocytes (193). Thus, the next generation of sensors is needed to detect dynamic cGMP nanodomains in cardiovascular cells. Even with the successful application of cAMP biosensors, there are drawbacks associated with delivering biosensors to targeted cells in the cardiovascular system. The application relies on either transgenic expression of the sensors in vivo or viral infection of isolated primary cells or tissues. The transgenic approach is time-consuming, and the overexpressed biosensors may cause a baseline phenotype by altering cardiovascular function in the transgenic mice. The viral infection approach is limited to in vitro primary culture, which may encounter primary cell dedifferentiation during extended culture periods. Additionally, detection of the cyclic nucleotides in local nanodomains is still beyond the optical resolution of the fluorescence microscope and relies on targeting the sensors with anchor proteins to subcellular domains. Such a strategy could interfere with the local balance of cAMP signaling. Furthermore, during neurohormonal stimulation and under pathological conditions, the anchoring proteins may be relocated and lose fidelity in nanodomain targeting. Therefore, the signals detected with biosensors need to be corroborated with other independent evidence. Novel tools and techniques are needed to detect subcellular local cyclic nucleotide signals with high sensitivity and precision (185). As of today, most studies are based on isolated cells in vitro, and only one recent study shows the cAMP signals detected with a FRET-based sensor in the whole heart with optical mapping (73). Additionally, a recently reported single fluorophore-based cAMP biosensor has been successfully applied to detect cAMP in brain tissues in vivo (194). Technically, it is much simpler to perform single-fluorophore detection compared with the detection of FRET-based biosensors. This sensor could present a better tool for visualizing cAMP signals in the cardiovascular system in vivo. Overall, many technical challenges remain in visualizing cyclic nucleotide signaling in the cardiovascular system in vivo.

In summary, with their distinct localization, dynamics, and functions, compartmentalized cAMP and cGMP pools work in concert to regulate cardiovascular functions, from myocardial ECC to gene expression and pathological remodeling. Because of the pervasive functional role of cAMP and cGMP in the cardiovascular system, small molecules and proteins that modulate cyclic nucleotide signaling have attracted attention as the center of cardiovascular drug development programs. Hence, a detailed understanding of the PDEs in organizing and regulating the function of cAMP and cGMP nanodomains in the heart and vessels becomes increasingly essential (FIGURE 4).

## 3. PDEs IN THE HEART

The heart functions as a pump, continuously circulating blood through each cardiac contraction and relaxation. The contraction and relaxation cycle is achieved through cardiac ECC, a process predominantly controlled by intracellular Ca^2+^ (*I*_Ca_) (195). The depolarized plasma membrane activates voltage-gated LTCCs upon electrical excitation from pacemaker cells, causing Ca^2+^ influx (*I*_Ca,L_) in myocytes (195). This Ca^2+^ entry triggers more Ca^2+^ release from the SR through RyR2 (196). The Ca^2+^ influx and release raise *I*_Ca_, which binds to myofilament proteins such as troponin C to activate the myocyte contractile cycle (195). After the peaking of *I*_Ca_ concentration, *I*_Ca_ declines through SERCA2a- and sodium/calcium exchanger-mediated calcium uptake and efflux, respectively, which leads to Ca^2+^ myocyte relaxation (197).

**FIGURE 5.**
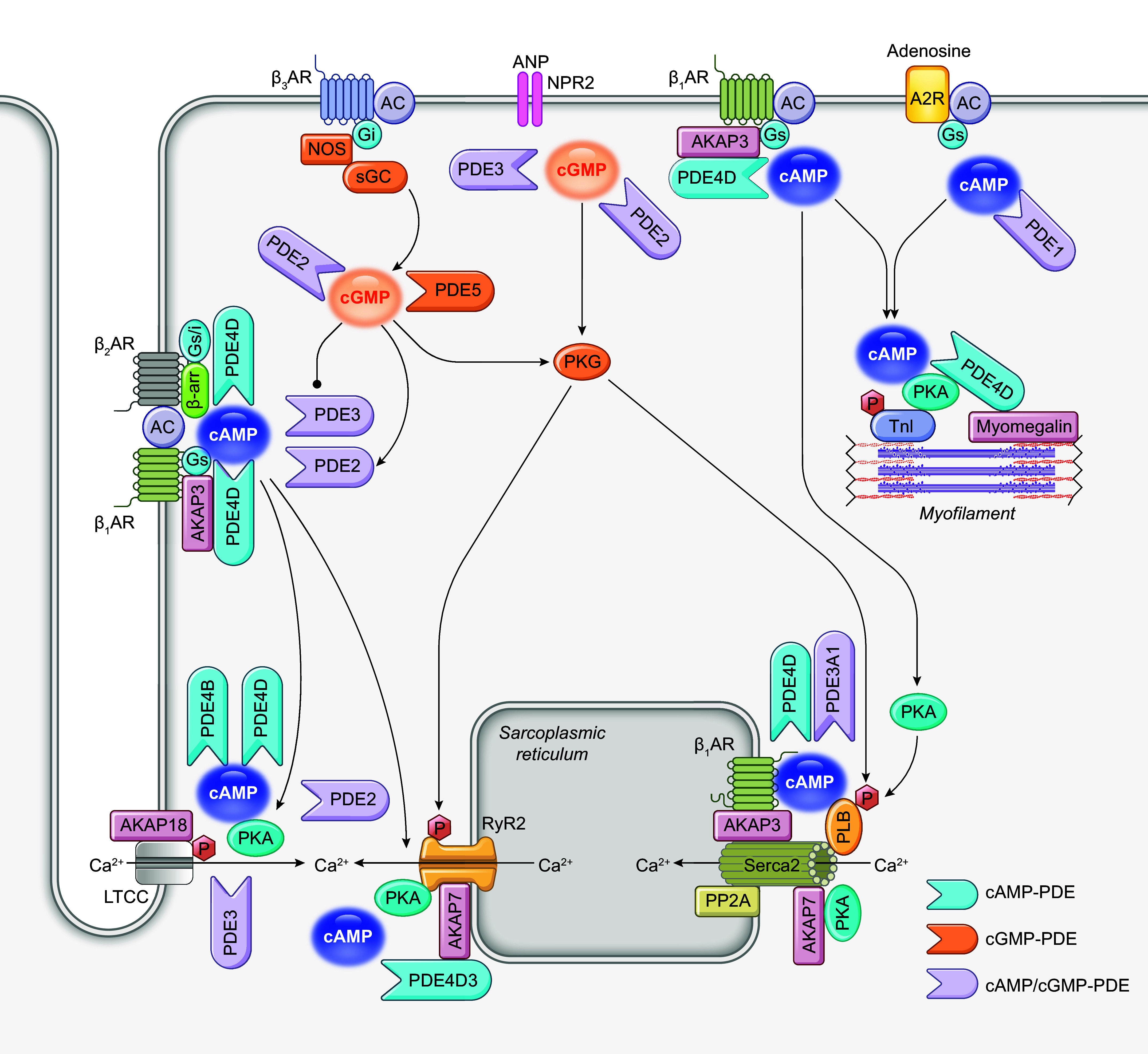
Schematic illustration of phosphodiesterases (PDEs) in cardiac excitation-contraction coupling. A2R, adenosine receptor 2, AC, adenylyl cyclase; ANP, atrial natriuretic peptide; AKAP, A-kinase anchoring protein; β-AR, β adrenergic receptor; cAMP, cyclic adenosine monophosphate, cGMP, cyclic guanosine monophosphate; Gi, inhibitor G protein; Gs, stimulatory G protein; LTCC, L-type calcium channel; NPR2, natriuretic peptide receptor 2; PKA, protein kinase A; PKG, protein kinase G; PLB, phospholamban; PP2A, protein phosphatase 2A; RyR2, ryanodine receptor 2; SERCA, sarco(endo)plasmic reticulum calcium ATPase; sGC, soluble guanylyl cyclase; TnI, troponin I.

β-Adrenergic stimulation plays a critical role in the sympathetic regulation of cardiac output as part of the fight-or-flight response (FIGURE 5) (198). Sympathetic stress releases norepinephrine and epinephrine from the sympathetic nerve and adrenal gland to activate the βARs in cardiomyocytes (199). Activation of βARs increases cAMP and cGMP signals, which play a critical role in regulating cardiac ECC (200). For example, cAMP-activated PKA phosphorylates various downstream targets involved in calcium cycling or myofilament bridge formation (195). PKA can phosphorylate LTCC, RyR2, and phospholamban (PLB) to enhance *I*_Ca,L_, Ca^2+^ release, and Ca^2+^ uptake, yielding increases in *I*_Ca_ (199). The PKA-mediated phosphorylation of myofilament proteins, including troponin I (TnI) and myofilament binding protein C (MyBPC), modulates myocyte contractility without affecting Ca^2+^ cycling (199). Although the elevation of cAMP is prominent in enhancing ECC, the role of cGMP signal in ECC is less convincing. The literature suggests that cGMP negatively regulates contractility by suppressing the activity of Ca^2+^-handling proteins, including LTCC, RyR2, and SERCA2a, and myofilament Ca^2+^ sensitivity (201).

Reduced global cAMP is a hallmark of various cardiac diseases, including hypertrophy, myocardial infarction (MI), and HF (134). Local cAMP and cGMP undergo distinct alterations at subcellular compartments in different etiologies (152, 190, 202–204). In this section, we discuss the roles of PDEs in regulating cardiac contractile function and electrical rhythm, as well as in the pathogenesis of cardiac diseases.

### 3.1. PDE in the Regulation of Cardiac Contractility

#### 3.1.1. PDE in the regulation of Ca^2+^ influx by LTCC.

Among PDEs expressed in cardiomyocytes, PDE3 and PDE4 are pivotal regulators of myocyte *I*_Ca,L_ by controlling cAMP nanodomains at the sarcolemma because of their association or proximity to βAR or LTCC (76, 164, 165, 170, 205, 206). Whereas PDE3 and PDE4 are the dominant PDE subtypes involved in the regulation of basal *I*_Ca_, all four PDEs (PDE1–4) determine the response of *I*_Ca,L_ to a stimulus activating cAMP production, with the rank order of potency PDE4 > PDE3 > PDE2 > PDE1 (207). In addition, the deficiency of PDE8A potentiates an adrenergic-induced pool of cAMP to increase *I*_Ca,L_ and *I*_Ca_ (116).

PDE4 is the major PDE expressed in rodent hearts. PDE4A, PDE4B, and PDE4D have been identified in hearts. Specifically, PDE4D3, PDE4D5, PDE4D8, and PDE4D9 have been demonstrated to regulate β-adrenergic signaling and ECC (14, 208). At the sarcolemma membrane, PDE4D isoforms are physically associated with cardiac βARs, either through direct binding or with the assistance of arresting scaffolding (148, 161–166). The association modulates homologous and heterologous receptor desensitization. The interaction with PDE4D5 also prevents the Epac-dependent stimulation of CaMKII. Nonselective inhibition of PDE4 with rolipram potentiates the amplitude and duration of cAMP in cardiomyocytes in response to βAR stimulation (205). Rolipram also induces a drastic increase in PKA activity at the PM and a lesser significant increase at the myofilament and SR and affects myocardial cAMP-PKA-dependent phosphorylation of Ca^2+^-handling proteins and contractility (152). Consequently, βAR stimulation of *I*_Ca,L_, Ca^2+^ transient, and contractility is enhanced by PDE4 inhibition, indicating that PDE4 is a potent negative regulator of LTCC in hearts (76, 209). Meanwhile, PDE4B is also functionally distributed at the sarcolemma membrane (210) and couples with LTCC in myocytes (170). At the baseline, deleting PDE4B but not PDE4D increases *I*_Ca,L_, Ca^2+^ transient, and contraction (170).

PDE3 blockade alone has a minor effect on subsarcolemmal cAMP and LTCC, which is also unrelated to inotropy (211). However, when both PDE3 and PDE4 are inhibited, or when all PDEs are blocked with 3-isobutyl-1-methylxanthine, cAMP signal and *I*_Ca,L_ are sustained for an extended period (76). Phosphoinositide 3-kinase γ (PI3Kγ) is found to participate in multiprotein complexes linking PKA to the activation of PDE3A, PDE4A, and PDE4B but not of PDE4D. These PI3Kγ-regulated PDEs lower cAMP and limit PKA-mediated phosphorylation of LTCC and PLB, leading to increased Ca^2+^ spark occurrence and amplitude on adrenergic stimulation (77). In cardiac hypertrophy, PDE3 inhibition has an enhanced role in promoting *I*_Ca,L_ induced by βARs (209).

Despite its minimal expression in cardiomyocytes and modest contribution to the total cAMP hydrolytic activity (∼3%), PDE2 also modulates cardiac contractility (9, 212). The regulatory function of PDE2 in shaping βAR responses is closely linked to the refined phosphorylation of downstream targets, such as LTCC (213, 214). PDE2 attenuates the cAMP signal, reducing LTCC phosphorylation at PKA-specific sites and *I*_Ca,L_ in ventricular myocytes (214–216). PDE2 inhibition increases the inotropic effects of β_2_AR stimulation in rat left ventricular myocardium ex vivo, likely via potentiating the β_2_AR-cAMP signaling (217). Transgenic mice with cardiac-specific overexpression of PDE2 exhibit lower heart rates and display attenuated isoprenaline-induced increases in cAMP signal, *I*_Ca,L_, Ca^2+^ transient, sarcomere shortening, and arrhythmia (218). Moreover, PDE2 and PDE3 within βAR nanodomains undergo redistribution in HF animal models, influencing cardiac responses and contractility (172, 219).

Meanwhile, PDE2 can be activated by cGMP binding to the GAF domain. Multiple groups have reported that a cGMP-induced activation of PDE2 reduces *I*_Ca,L_ (220). For example, activation of β_3_AR transduces the NOS3-NO-cGMP signaling cascade and activates PDE2 (45). In this case, PDE2 inhibition markedly increases norepinephrine-induced cAMP responses, even though PDE2 inhibition alone leads to a minor increase in intracellular cAMP levels (45). Although the expression of β_3_AR in the myocardium remains contentious (221), the observation suggests that a β_3_AR-NO-cGMP-PDE2 axis confines the distribution of βAR-generated cAMP (45), consistent with a negative inotrope of NO (43). However, the PDE2-mediated cGMP-cAMP cross talk downstream of β_3_AR signaling is impaired in an MI-induced failing HF, because of the altered distribution of β_3_AR and sGC, leading to a decrease in cGMP level and impaired cGMP-cAMP cross talk (159). NPR1 is found in T-tubular membranes, and atrial natriuretic peptide (ANP)-stimulated cGMP is usually constrained at the membrane and does not affect cardiac inotropy and contractility (222). However, PDE2 inhibition augments the NPR-induced cGMP at the PM in adult cardiomyocytes (99). In this scenario, the ANP-stimulated cGMP also diffuses into the cytoplasm to promote the phosphorylation of PLB and regulate Ca^2+^ cycling (160). PDE2 expression is increased in human and pig models of dilated cardiomyopathy (42) and in a chronic ISO-induced rat HF model (42). The upregulation of PDE2A desensitizes acute βAR responsiveness in failing hearts (42). Additionally, PDE2 is relocalized from β_1_AR-associated noncaveolar into β_2_AR-containing caveolar fractions in cardiac hypertrophy after transaortic constriction (TAC) (219).

Thus, PDE4 appears to be the most crucial regulator in enhancing LTCC activity in physiological response, whereas PDE2 and PDE3 play additional roles in coordinating the regulation. Meanwhile, PDE2 and PDE3 may have enhanced roles in regulating cAMP, cGMP, and LTCC activity in cardiac diseases.

#### 3.1.2. PDE in the regulation of SR Ca^2+^ release and uptake.

PDE4, PDE3, and PDE2 are implicated in regulating SERCA2a and RyR2 activities and SR Ca^2+^ release and uptake (214, 223, 224). With FRET biosensors, PDE4 and PDE3 are shown to control the baseline PKA activity at the RyR2 and SERCA2a nanodomains and prevent β_2_AR signaling from reaching these nanodomains in mice, rat, and rabbit myocytes (152). PDE4 is the most determinant when cAMP levels are elevated upon βAR stimulation (214). However, PDE2 and PDE3 also play a prominent role in regulating cardiac contraction and Ca^2+^ transients at the baseline and after βAR stimulation (214, 223, 224).

PDE4 isoforms associated with SERCA2a affect the Ca^2+^ pump activity and SR Ca^2+^ uptake. PDE4D coimmunoprecipitates with SERCA2a in murine and failing human hearts (173) and negatively regulates βAR stimulation of PLB phosphorylation and SR Ca^2+^ load (206). PI3Kγ is required for PDE4, not PDE3, activity in the subcellular SERCA2a nanodomains in cardiomyocytes, and loss of PI3Kγ selectively abolishes PDE4 activity in the SERCA2a nanodomains (206). Recently, a pool of intracellular β_1_AR has been identified with SERCA2a (199, 203, 225), suggesting that a local β_1_AR signaling machinery at the SR regulates cAMP levels at the SERCA2a nanodomains. Mice lacking PDE4D have an enhanced baseline cardiac contractility associated with increased PLB phosphorylation, SR Ca^2+^ content, and Ca^2+^ transients (173). The phosphorylation of PLB and SERCA2a function is depressed in HF (203, 226–228). PDE4 is reduced in hypertrophic cardiomyocytes (190), which may be a compensatory adaptation and may help to increase the phosphorylation of PLB and SR Ca^2+^ uptake. In comparison, the contribution of PDE2 to cAMP hydrolysis is increased in the vicinity of SERCA2a in hypertrophied myocytes, which may affect Ca^2+^ cycling and contractility (190).

PDE4D3 forms a complex with RyR2 at the SR in human and mouse hearts and regulates the PKA-dependent phosphorylation of RyR2 (169). Selective inhibition of PDE4 enhances the adrenergic stimulation of local cAMP signals at the RyR2 (202). Deficiency of PDE4D leads to PKA hyperphosphorylation of RyR2, promoting RyR2 dissociation from the stabilizing protein calstabin2 (FKBP1.2), aberrant calcium release, arrhythmias, and the development of dilated cardiomyopathy (169). In hypertrophic hearts, the RyR2-associated PDE4 is reduced, leading to an increased RyR2 phosphorylation to β_2_AR stimulation (169).

Deleting PDE3A increases PLB phosphorylation, SERCA2a activity, SR Ca^2+^ uptake, and Ca^2+^ contents and promotes cardiac contractility and relaxation through cAMP-dependent elevations of Ca^2+^ transients (63). Consequently, PDE3 inhibition enhances contractility in vitro and in vivo (63). Although PDE3A and PDE3B are expressed in the heart (61, 62), knockout experiments suggest that PDE3A but not PDE3B regulates cardiac contractility (63, 64). PDE3A1 associates with SERCA2a in human hearts, forming a signalosome consisting of multiple proteins, including AKAP18, PP2A, PP1, PLB, and caveolin 3 (60, 63). Disrupting the association between PDE3A and SERCA2a with a disruptor peptide increases the Ca^2+^ pump activity in normal and failing cardiomyocytes (229). After aortic banding, mice injected with recombinant adeno-associated virus 9 (rAAV9) expressing the disruptor peptide have improved contractility but no difference in cardiac remodeling compared to mice with control rAAV9 (229). A recent study shows that the hypertension-related gain-of-function mutations of PDE3A do not affect overall adrenergic stimulation and cAMP levels but reduce PLB phosphorylation, which leads to adaptive change in Ca^2+^ cycling and protects against hypertension-induced cardiac damage in the hearts (230).

#### 3.1.3. PDE in the regulation of Na^+^-K^+^-ATPase activity.

PDEs also affect cardiac contractility by modulating phospholemman-mediated Na^+^-K^+^-ATPase activity. Na^+^-K^+^-ATPase indirectly affects myocyte *I*_Ca_ through the sodium/calcium exchanger. Phosphorylation of phospholemman, the Na^+^-K^+^-ATPase regulator, enhances the pump activity and results in a decrease of intracellular Na^+^. The decrease of intracellular Na^+^ reduces *I*_Ca_ and myocyte contractility. PDEs fine-tune the PKA phosphorylation of phospholemman and, therefore, the changes in contractility. PDE3 has been shown to control cAMP in a nanodomain around the phospholemman-Na^+^-K^+^-ATPase complex and preferentially regulates this cAMP pool induced by β_2_AR (172). Moreover, NPR2 stimulation by C-type natriuretic peptide (CNP) enhances cAMP signaling through the cGMP-mediated inhibition of PDE3 and improves contractility in normal and failing hearts (231). In comparison, NPR1 stimulation by ANP or B-type natriuretic peptide (BNP) does not affect βAR signaling (232). In rats after MI-induced chronic HF, a significant increase in PDE2-mediated hydrolysis of cAMP downstream of β_2_AR is observed within the phospholemman Na^+^-K^+^-ATPase nanodomains, whereas the PDE3-induced hydrolysis of cAMP is diminished (172). These data demonstrate that PDEs act together to fine-tune cAMP at the Na^+^-K^+^-ATPase nanodomains in the heart.

#### 3.1.4. PDE in the regulation of myofilament cGMP signaling.

The literature points out the prominent roles of PDE1 in regulating cGMP and PKG activity and myofilament relaxation. PDE1C expression is conserved in rabbits, dogs, and humans, which opposes the PDE1A expression in mice and rats (233). A selective PDE1 inhibitor, ITI-214, evokes positive inotropic, lusitropic, chronotropic, and vasodilator effects in normal-conscious dogs (233). These responses are preserved except for heart rate in the same animals when their hearts are induced into failure after 3–4 wk of tachycardia pacing. These cardiac and systemic arterial effects of ITI-214 have been linked to adenosine A2B receptor-induced cAMP signaling (233). A2B receptor stimulation is well known to protect against ischemic injury (234); the protective effects of the A2B receptor may be leveraged by PDE1 inhibition. In cardiomyocytes, PDE1 inhibition augments the resting cell contraction without increasing Ca^2+^ (235). By contrast, β_1_AR stimulation or PDE3 inhibition increases cAMP, Ca^2+^ transients, and cell contraction. The PDE1 inhibitor ITI-214 thus may selectively target myofilaments to induce positive inotropy and lusitropy, differing from that coupled to β_1_AR-cAMP signaling. Similarly, biased β_1_AR-G_i_ signaling induced by carvedilol selectively promotes cGMP-PKG-mediated phosphorylation of myofilament proteins and cardiac contractility without increasing Ca^2+^ cycling (200). The mechanism underlying PDE1-induced contraction remains to be explored. PDE1 inhibition may have a safety profile different from those observed with PDE3 inhibitors that induce cardiac arrhythmia.

PDE3 regulates global cellular cGMP levels in normal and failing hearts (236). PDE3 has been shown to constrain CNP-stimulated cGMP at the PM, PLB, and myofilament compartments (237). CNP also promotes cGMP-dependent inhibition of PDE3, which increases cAMP through the cGMP-cAMP cross talk. NPR2-mediated cGMP production causes a negative inotropic and a positive lusitropic response in failing hearts (238). Inhibition of PDE3 enhances the CNP-mediated lusitropic response in normal heart muscle and the CNP-mediated negative inotropic and positive lusitropic responses in HF models. CNP also enhances β_1_AR- and β_2_AR-mediated inotropic and β_1_AR-mediated lusitropic responses in nonfailing and failing hearts through the cGMP-cAMP cross talk where cGMP increases cAMP signal by inhibiting PDE3 (231). Furthermore, CNP also enhances the serotonin type 4 (5-HT4)-mediated inotropic response in failing rat heart ventricles via PDE3-mediated cGMP-cAMP cross talk (239). In the long term, this CNP-cGMP-PDE3 inhibitory pathway may be detrimental to failing hearts through mechanisms like those treated with PDE3 inhibitors or β-adrenergic stimulation (238).

Inhibition of PDE5A attenuates the catecholamine-induced contractile function through cGMP-PKG signaling. For instance, a PDE5 inhibitor, sildenafil, attenuates dobutamine and ISO-stimulated cardiac contractility (232, 240, 241). Interestingly, deletion of β_3_AR abolishes the sildenafil-mediated negative regulation of contractility (242), indicating that activation of the β_3_AR-NOS pathway is necessary for the sildenafil effect on increasing cGMP signal. The effects of PDE5 inhibitors on cardiac contractility are largely diminished in hypertrophic failing hearts, which is associated with reduced Z-band localization of PDE5A (241). Another report shows that PDE5 is coupled to β_2_AR in cardiomyocytes isolated from mice after high-fat diet feeding. Inhibition of PDE5 enhances the β_2_AR-induced cAMP and cGMP signals and PKG-dependent myocyte contractility (243). These findings implicate a shift coupling of PDE5 to different βAR subtypes to regulate highly localized cGMP pools and cardiac contractility in normal and pathological hearts.

PKA-dependent phosphorylation is also critical in modulating myofilament contraction in physiological and disease states (244–247). However, more information is needed to understand the PDE regulation of cAMP signaling and function on the myofilaments. PDE3 and, to a lesser extent, PDE4 and PDE2 regulate the baseline PKA activity at the myofilaments in rabbit myocytes, whereas inhibition of PDE3 and PDE4 enhances the βAR-induced PKA activity (152). In failing rabbit hearts, loss of caveolin 3 leads to reduced impacts of PDE3 and PDE2 on myofilament PKA activity, whereas no change is observed in the impact of PDE4 (152).

### 3.2. PDE in the Regulation of Heart Rate and Cardiac Arrhythmias

**FIGURE 6.**
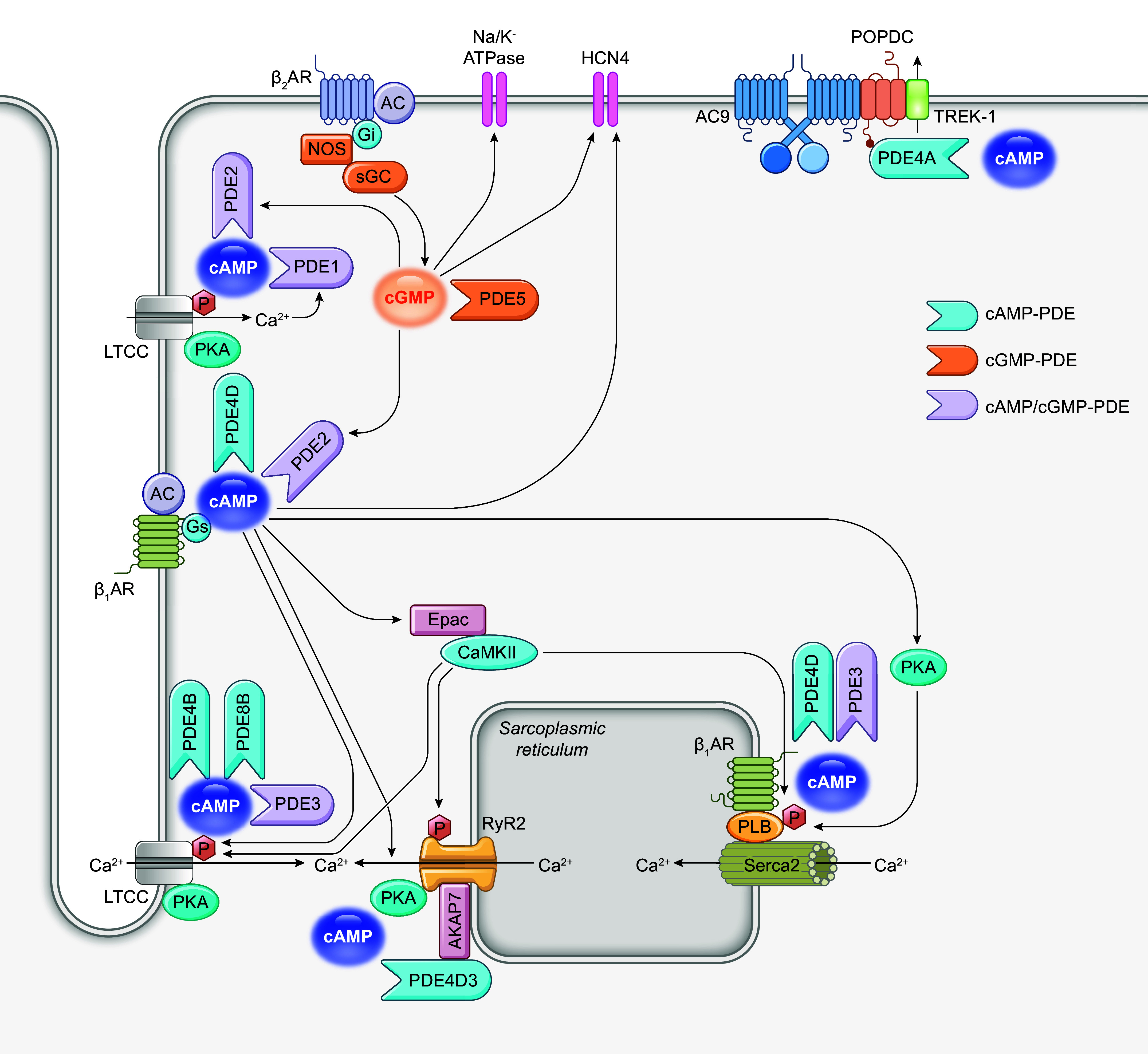
Schematic illustration of phosphodiesterases (PDEs) in the regulation of heart rate and arrhythmia. AC, adenylyl cyclase; AKAP, A-kinase anchoring protein; β_1_AR, β_1_ adrenergic receptor; β_2_AR, β_2_ adrenergic receptor; β-arr, β-arrestin; CaMKII, Ca^2+^/calmodulin-dependent kinase II; cAMP, cyclic adenosine monophosphate; cGMP, cyclic guanosine monophosphate; Epac, exchange protein activated by cAMP; Gi, inhibitor G protein; Gs, stimulatory G protein; HCN, hyperpolarization-activated cyclic nucleotide-gated channel; LTCC, L-type calcium channel; Na/K-ATPase, sodium-potassium-ATPase; NOS, nitric oxide synthase; P, phosphate; PKA, protein kinase A; PLB, phospholamban; POPDC, popeye domain containing protein; RyR2, ryanodine receptor 2; SERCA, sarco(endo)plasmic reticulum calcium ATPase; sGC, soluble guanylyl cyclase.

PDEs play a crucial role in regulating the rhythmicity of pacemakers across multiple dimensions (FIGURE 6). Sinoatrial node (SAN) cells initiate and time-keep heartbeat by a precise coupled system of an intracellular Ca^2+^ clock and a surface membrane voltage clock (248). SAN cells allow individual ion channel currents to ensemble to generate rhythmic action potentials. This ensemble is known as the membrane clock (M clock). The intracellular Ca^2+^ clock refers to RyR2-mediated Ca^2+^ release. In spontaneous firing SAN cells, the M clock and Ca^2+^ clock work in coordination fine-tuned by a multitude of interactions. PDE inhibition in SAN cells markedly augments Ca^2+^ cycling protein phosphorylation and accelerates the action potential (AP) firing rate by elevating the cAMP signal (249). Consequently, PDEs wield the potential to impact pacemaker activity through diverse mechanisms, encompassing the cAMP-PKA pathway and calcium signaling.

#### 3.2.1. PDE in the regulation of pacemaker activity.

The modulation of heart rate by PDE entails the precise regulation of cAMP and cGMP through a diverse array of effectors within SAN cells. As a predominant regulatory molecule, cAMP orchestrates alterations in the action potential of SAN cells through downstream effectors: hyperpolarization-activated cyclic nucleotide-gated channel 4 (HCN4) responsible for the “funny” current (*I*_f_), PKA, EPAC1, and popeye domain-containing (POPDC) proteins (230). Accordingly, manipulating PDEs could enhance or suppress pacemaking activities in SAN cells through these targets. For example, PKA activity underscores the PDE-dependent modulation of pacemaker flexibility. Stimulation of βARs increases cAMP-PKA signaling in the SAN cells to accelerate the spontaneous firing of SAN cells through two parallel mechanisms, which underlies the chronotropic effect under the fight-or-flight response. On one hand, cAMP directly binds to HCN4, increasing the activity of *I*_f_ and enhancing the M clock of SAN pacemaking. On the other hand, the cAMP-PKA signaling promotes PKA phosphorylation and activities of the key downstream proteins of ECC, including LTCC, RyR2, and PLB. These phosphorylation events increase cytosolic Ca^2+^ entry and enhance SR Ca^2+^ release through RyR2 and uptake via SERCA2a, promoting the Ca^2+^ clock to accelerate the action potential firing of SAN cells. The increased Ca^2+^ signals also activate CaMKII to phosphorylate LTCC, RyR2, and PLB to promote the Ca^2+^ clock (250).

PDE1 is an essential modulator of heart rate. PDE1 activity accounts for 39% of the total PDE activity in SAN cell lysates, compared to only 4% in left ventricular cardiomyocytes. PDE1 senses Ca^2+^-CaM signal to regulate the cAMP degradation in lipid raft domains and determine the intensity of Ca^2+^-AC-cAMP-PKA signaling that drives SAN pacemaker function (251). PDE2 reduces LTCC phosphorylation at PKA-specific sites and *I*_Ca,L_ in atrial cells by attenuating cAMP levels at the PM (213, 252, 253). PDE3 is the most predominantly expressed PDE in human atrial, including SAN, cells. Among PDE3 isoforms, PDE3A is more potent than PDE3B for cAMP degradation in regulating heart rate (64). PDE3 inhibition dramatically increases the basal spontaneous SAN cell beating rate, accompanied by a marked increase in PLB phosphorylation (213, 254). PDE3 inhibitors also enhance the prestimulated *I*_Ca,L_ and Ca^2+^ uptake and promote heart rate (255). Additionally, POPDC proteins, a group of newly characterized effectors of cAMP, play a crucial role in cardiac pacemaking. POPDC1 preferentially binds to the PDE4A subfamily via a specificity motif in the PDE4A UCR1 region. The PDE4 activity localized to POPDC1 modulates the cycle length of spontaneous Ca^2+^ transients firing in intact mouse SANs (256).

In SAN cells, cGMP regulates heart rate through various effectors, although the mechanisms in this context have been understudied. NO leads to an increase in *I*_f_ current and heart rate, partially due to NO-stimulated increases in cGMP (257). The stimulatory effects of cGMP on *I*_Ca_ in rabbit atrial cells are likely to be mediated via the PKG-dependent phosphorylation of LTCC or associated proteins. PKG is highly expressed in atrial cells, and PKG-dependent phosphorylation may be necessary for maintaining basal *I*_Ca_ and fully stimulating *I*_Ca_ by β-adrenergic activation (258). Additionally, cGMP can directly bind to HCN4 channels, which is key in determining membrane potential in pacemaker cells (259). Modulating HCN4 and *I*_f_ in SAN cells through phosphorylation or cyclic nucleotides can alter heart rate and cause bradycardia, tachycardia, and SAN dysfunction (259). In pacemaker cells, NO also induces cGMP-dependent activation of PDE2, which attenuates *I*_Ca,L_ and heart rate (201, 260). Accordingly, mice with cardiac-specific PDE2 overexpression exhibit lower heart rates and display blunted isoprenaline-induced increases in cAMP levels and heart rates (218). PDE2 also fine-tunes a specific cAMP pool generated downstream of β_2_ARs, which PDE5 indirectly controls. Inhibition of PDE5 promotes cGMP-dependent activation of PDE2, which has negative chronotropic effects (261). Epidemiological data indicate a reduction in cardiovascular events and mortality in PDE5 inhibitor users at high cardiovascular risk (262). The antiarrhythmia effects of PDE5 inhibition may involve cGMP, and the underlying mechanism requires further investigation.

#### 3.2.2. PDE in atrial fibrillation.

Atrial fibrillation, which is closely linked to PDE dysfunction, represents the most common arrhythmias. For instance, inhibition of PDE2 increases *I*_Ca,L_ in human atrial myocytes, and PDE2 overexpression attenuates SR Ca^2+^ release and arrhythmia susceptibility (218). PDE3 is the most abundant PDE in human atria. Chronic treatments with PDE3 inhibitors are an independent risk factor for clinically significant tachyarrhythmias, augmenting the mortality of treated patients (263, 264). Patients with permanent atrial fibrillation have been found to have decreased PDE4 activity (265). PDE4A, PDE4B, and PDE4D isoforms are present in human atrial myocytes, accounting for ∼15% of total PDE activity (265). PDE4B and PDE4D are tethered to LTCC in mouse hearts (170), whereas PDE4D3 is linked to RyR2 (169). PDE4B deletion has been linked with exacerbated βAR stimulation of *I*_Ca,L_, whereas PDE4D deletion leads to PKA-dependent hyperphosphorylation of RyR2 (169, 173). Consequently, PDE4 inhibition increases the LTCC density and the rate of spontaneous SR Ca^2+^ release in isolated human atrial myocytes and intact human atrial trabeculae exposed to βAR stimulation (265). PDE5 inhibitors have electrophysiological effects in atrial myocytes, which may contribute to a direct antiarrhythmic action during reperfusion, including reducing *I*_Ca,L_ and intracellular Na^+^, enhancing Na^+^-K^+^-ATPase activity (266), and suppressing β-adrenergic signaling (267). Additionally, PDE8A expression is increased in the human atrium in atrial fibrillation (268). Upregulation of the PDE8B2 isoform in chronic atrial fibrillation reduces *I*_Ca,L_ via direct interaction of PDE8B2 with the LTCC Cav1.2 α_1C_-subunit. Thus, the upregulated PDE8B2 governs a cAMP-PKA-dependent reduction of *I*_Ca,L_ in human atrial fibrillation, which might be a novel mechanism of the proarrhythmic reduction of *I*_Ca,L_ in chronic atrial fibrillation (269). Collectively, these findings highlight the PDEs’ significant impacts on atrial electrical activity and their potential roles in increasing the risk of arrhythmia. Arrhythmia-related sudden cardiac death accounts for up to 60% of deaths in HF patients (270). Strategies aiming at fine-tuning the activity of individual PDEs may offer effective therapies for cardiac arrhythmia in HF patients.

#### 3.2.3. PDE in ventricular arrhythmias.

PDEs are also involved in ventricular arrhythmias. Genetic ablation of PDE4B or PDE4D enhances the susceptibility to stress-induced ventricular tachycardia (169, 170). PDE2 activation protects against ventricular arrhythmias by preventing Epac- and CaMKII-mediated increases in *I*_Na_ and *I*_Ca,L_ and Ca^2+^ leakage from the SR (218, 271). In ischemia-reperfusion of isolated rat hearts, pretreatment with sildenafil protects against ventricular fibrillation and reduces infarct size while improving left ventricle recovery, probably through a cGMP signaling pathway (272). Long QT syndrome type 2 (LQT2) arrhythmogenesis is mainly ascribable to the facilitation of Ca^2+^ waves, reflecting SR instability. An antiarrhythmic effect of PDE5 inhibition has been reported in long QT syndrome by inhibiting the SERCA2a-mediated Ca^2+^ uptake and reducing SR Ca^2+^ content (262). Therefore, whereas the elevation of cAMP is linked to increases in ventricular arrhythmicity, the elevation of cGMP has a protective effect.

### 3.3. PDE in Cardiac Hypertrophy, Apoptosis, and Heart Failure

HF is a severe and complex pathology that remains a major cause of global morbidity and mortality. In response to stressors such as hypertension or neurohumoral activation, the heart undergoes pathological changes, including increased myocyte size and protein synthesis, cell growth, and myocyte apoptosis. These changes lead to cardiac hypertrophy and ventricular remodeling, resulting in decompensation and HF. Current HF treatments primarily aim at preventing ventricular remodeling and improving overall cardiac function.

**FIGURE 7.**
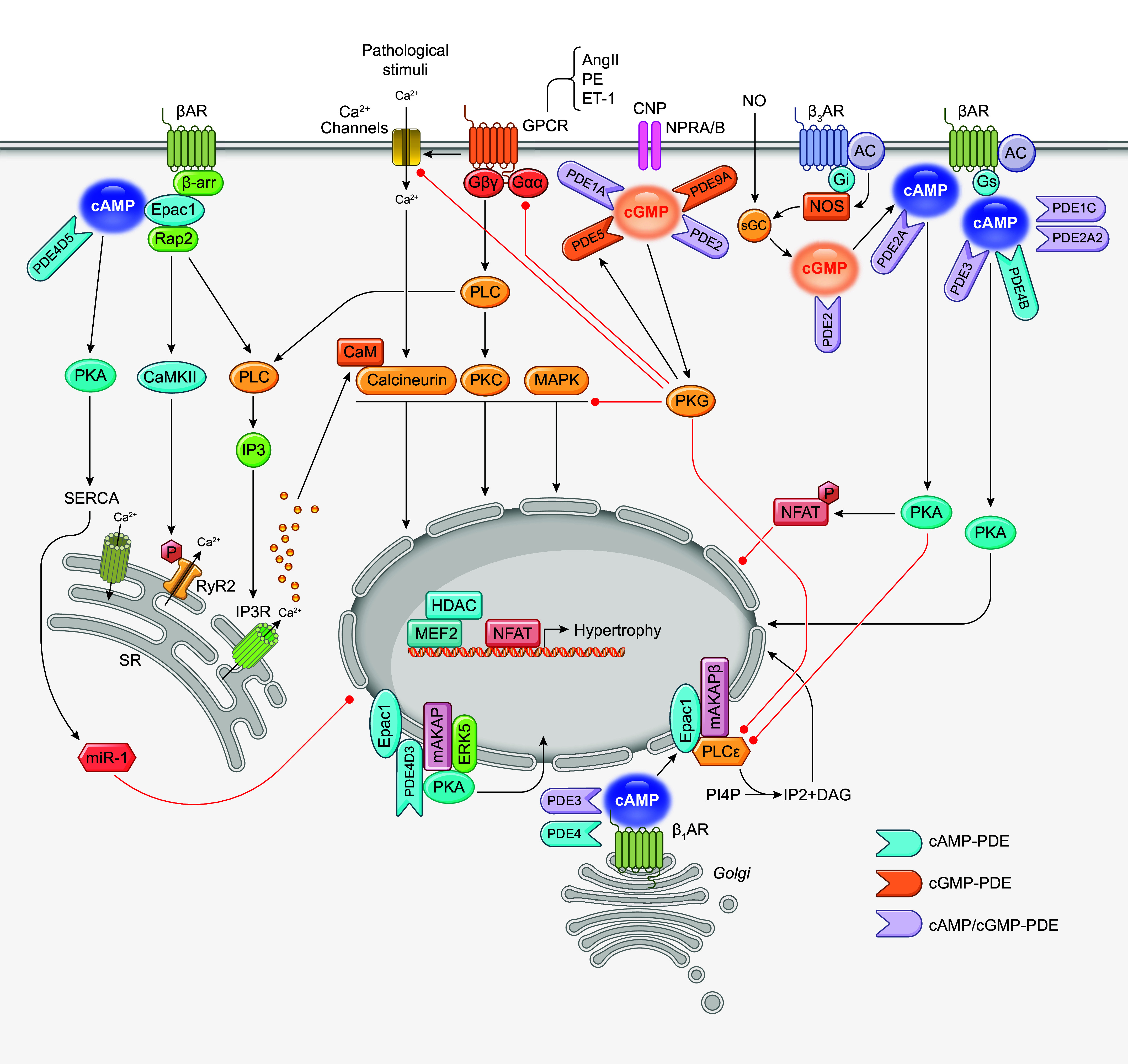
Schematic illustration of phosphodiesterases (PDEs) in the regulation of cyclic adenosine monophosphate (cAMP) and cyclic guanosine monophosphate (cGMP) in cardiac hypertrophy. The cartoon highlights the subcellular regulation of cAMP and cGMP induced by different neurohormonal stimuli in driving cardiac hypertrophy. AC, adenylyl cyclase; AngII, angiotensin II; AKAP, A-kinase anchoring protein; βAR, β adrenergic receptor; β-arr, β-arrestin; CaMKII, Ca^2+^/calmodulin-dependent kinase II; CNP, C-type natriuretic peptide; DAG, diacylglycerol; Epac, exchange protein activated by cAMP; ET-1, endothelin-1, Gαq, Gαq protein; Gβγ, Gβ and Gγ protein; Gi, inhibitor G protein; GPCR, G protein-coupled receptor; Gs, stimulatory G protein; HDAC, histone deacetylase; IP2, inositol-4,5-bisphosphate; IP3, inositol 1,4,5-trisphosphate; IP3R, inositol 1,4,5-trisphosphate receptor; MAPK, mitogen-activated protein kinase; MEF2, myocyte enhancing factor 2; miR-1, microRNA-1; NFAT, Nuclear factor of activated T cells; NPR2, natriuretic peptide receptor 2; NOS, nitric oxide synthase; PE, phenylephrine; PI4P, phosphatidylinositol-4-phosphate; PKA, protein kinase A, PKC, protein kinase C; PLC, phospholipase C; Rap1, ribosome activated protein 1; RyR2, ryanodine receptor 2; SERCA, sarco(endo)plasmic reticulum calcium ATPase; sGC, soluble guanylyl cyclase.

cAMP and cGMP play a key role in cardiac function in normal and pathological conditions (FIGURE 7). Chronic HF is also characterized by overexcitation of the sympathetic nervous system and the release of catecholamines (262). The subsequent activation of cardiac βARs induces cardiac hypertrophy and apoptosis via the cAMP and PKA-mediated elevation of *I*_Ca_ and CaMKII (263). Chronic β-adrenergic stimulation also activates Epac. Whereas PKA is proapoptotic, Epac protects myocytes via activation of MAPK (273). These distinct pathways may be underlying the observations in which selectively enhancing the type VI AC-induced cAMP-PKA signaling benefits failing hearts (274). Moreover, β-adrenergic stimulation promotes an Epac-phospholipase Cε (PLCε)-dependent phosphatidylinositol 4-phosphate (PI4P) hydrolysis, acting as a critical process for cardiac hypertrophy (275).

Despite elevated sympathetic activity, HF is often associated with reduced intracellular content of cAMP and PKA substrate phosphorylation, contributing to impaired cardiac contractility. Clinically, PDE3 inhibitors are developed to attenuate the degradation of cAMP, leading to increased intracellular calcium and improved hemodynamics and exercise capacity in patients with advanced HF (276). However, despite the short-term functional gains, therapy aimed at increasing cAMP levels increases mortality in the long term (277). This raises the question of how we can develop a strategy to trigger only the inotrope response in HF. One can speculate that cAMP signaling controls various and opposing cellular functions via selective activation of different targets residing at subcellular locations. Distinct subcellular cAMP pools and different cAMP levels may have opposing effects on myocyte contractility, growth, and death (155, 278). Another consideration is the interplay between cAMP and cGMP signals. Notably, cGMP can affect cAMP levels in cardiomyocytes by activating or inhibiting cAMP-hydrolyzing PDEs (277). Compared with the cAMP signaling pathway, cGMP mediates the cardiac effects of NO, which has negative inotropic effects. The cGMP-PKG signaling also negatively regulates cardiac hypertrophy (279). Moreover, the modulatory effects of cGMP on cAMP-hydrolyzing PDEs are titrated by subcellular concentrations of cGMP (201). The balance of cAMP and cGMP signals may have a “yin-yang” effect, two opposing forces in harmony, in cardiovascular homeostasis (280). The intricate cross talk between cAMP and cGMP can have divergent downstream actions for a broad range of functional outcomes (FIGURE 7). Accordingly, the functional diversity of individual PDEs could be achieved through the localization, the link to discrete pools of cyclic nucleotides, and the association with downstream signaling molecules. Under pathological environments, the regulation and function of individual PDEs are disrupted, contributing to the progression of cardiac hypertrophy and HF (TABLE 2). In sects. 3.3.1–3.3.3, we discuss how PDEs are involved in the development of hypertrophic HF.

**Table 2.** The roles of individual PDE isoforms in cardiac hypertrophy

PDE Family	Isoform	Variants	Expression	Activity	Pathology	Model in vivo	Model in vitro	Genetic Intervention in vivo	Genetic Intervention in vitro	Inhibitors Used	Positive ↑Negative ↓	Potential Mechanism	References
PDE1						Ang II infusion mice				vinpocetine	↓	↓PDE1	(281)
	PDE1A		↑			ISO infusion mice	Ang II and ISO-NRCM		PDE1A shRNA	IC86340	↓	↑cGMP-PKG	(23)
	PDE1C		↑			TAC		PDE1C null mice			↓	↑cAMP-PKA	(26)
PDE2			↑	↑	Human HF, TAC								(42)
						AAC	Ang II-NRCM			BAY 60-7550	↓	↑NO-GC-cGMP signaling	(43)
						TAC	NE-NRVM			BAY 60-7550	↓	↑PKA-NFAT phosphorylation	(278)
							ET-1-NRVM			BAY60-7550	↓	↓ cAMP-PKA-dependent inhibition of PLCε; ↓cGMP-PKG-dependent inhibition of PLCε	(275)
	PDE2A					Male rats per se	ARVMs per se	PDE2A overexpression rats			↑	↓PKA-mediated NFAT phosphorylation	(278)
		**PDE2A2**					**NE/PE-ARVM**		**PDE2A2 adenovirus**		**↓**	**↓β-Adrenergic responses**	**(42)**
PDE3					Human HF								(282, 283)
				↓	TAC								(284)
			↑	↑	Salt-induced HF rat								(285)
				↑	TAC					Milrinone	↓	↓p38 MAPK, ↓calcineurin-NFAT	(286)
							**NRCMs per se**			**Cilostamide**	**↑**	**↑ PLCε at the Golgi through Epac →↑PI4P hydrolysis**	**(275)**
							**NRCMs per se**			**Cilostamide**	**↑**	**↑ PKA not anchored to AKAPs**	**(278)**
	PDE3A		↓	↓	Human DCM and IHD, TAC								(58)
	PDE3A		↓		TAC								(284)
	PDE3A					TAC		PDE3A-KO			↓		(286)
		**PDE3A2**					**NRCMs per se**		**Inactive mutants of PDE3A2**		**↑**	**↑cAMP at sites where prohypertrophic effectors**	**(278)**
	PDE3B					TAC		PDE3B-KO					(286)
PDE4			↑	↑	Salt-induced HF rat								(285)
				↑	Human HF with diabetes, HFD mice					Roflumilast	↓	↑cAMP-CREB-Sirt1- SERCA2a- miR-1	(83)
							**NRCMs per se**			**Rolipram**	**↑**	**↑Epac-PLCε at the Golgi →↑PI4P hydrolysis**	**(275)**
							**NRCMs per se**			**Rolipram**	**↑**	**↑PKA not anchored to AKAPs**	**(278)**
							**ISO-NRCMs**		**PDE4 activator UCR1C**		**↓**	**↓Nuclear PKA -CREB**	**(287)**
	PDE4A		↓		TAC								(284)
	PDE4B		↓		TAC								(284)
			**↓**		**Human HF**	**ISO infusion, TAC**		**PDE4B-TG mice, AAV9-PDE4B**			**↓**	**Blunts β-adrenergic response**	**(288)**
	PDE4D				TAC								(284)
			↑		Human HF with diabetes, HFD mice			AAV9-PDE4D shRNA		Roflumilast	↓	↑cAMP-CREB-Sirt1-SERCA2a- miR-1	(83)
		PDE4D3					LIF-NRCM			Rolipram	↓	↓mAKAP-PDE4D3-Epac1-PKAcomplex anchored ERK5 activity	(155)
							**NRVMs per se**		**Inactive mutants of PDE4D3**		**↑**		**(278)**
		PDE4D5	↑		Human HF with diabetes, HFD mice		NRVM		PDE4D5 adenovirus		↑	↓cAMP-CREB-Sirt1-SERCA2a- miR-1	(83)
							**ISO-NRCM**		**competitive peptide 58-Pept**		**↑**	**↓β-arrestin–PDE4D5 interaction→ ↑ Epac1 recruit to β2AR**	**(163)**
PDE5	PDE5A		↑		HF (DCM, ICM)	MI		PDE5-TG			↑	↓cGMP	(92)
						TAC		PDE5-TG			↑	↑Natriuretic peptide-derived cGMP hydrolysis	(289)
			↑		CHF, TAC	TAC				Sildenafil	↓	↓Oxidative stress	(290)
						TAC				Sildenafil	↓	↑cGMP-↓oxidized PKG1α	(291)
						TAC				Sildenafil	↓	Improve T-tubule remodeling	(292)
							PE-NRCM		PDE5A-shRNA	Sildenafil	↓	↑PKG	(91)
						TAC	PE-NRCM			Sildenafil	↓	↓Calcineurin/NFAT, ↓PI3K/Akt, ↓ERK1/2	(293)
						SHR				Sildenafil	↓	↓Na^+^/H^+^ exchanger activity	(294)
						TAC	PE-NRCM			CRD-733	↓	↑cGMP	(295)
PDE9	PDE9A		↑		Human (DCM, HFpEF), aortic stenosis, TAC	TAC	PE, ET-1-NRCM	PDE9A-KO	PDE9A-siRNA	PF-9613	↓	↑natriuretic peptide-cGMP	(123)
							ET-1-NRCM			PF04449613	↓	↓cGMP-PKG-dependent inhibition of PLCε	(275)
						ISO rat	PE, ISO-NRCM			C33(S)	↓	↑cGMP	(296)

Boldface indicates that the phosphodiesterase (PDE) plays a negative role in hypertrophy. AAC, abdominal aortic constriction; AAV9, adeno-associated viral vector serotype 9; AKAP, A-kinase anchoring protein; Ang II, angiotensin II; ARVM, adult rat ventricular myocyte; cAMP, cyclic adenosine monophosphate, cGMP, cyclic guanosine monophosphate; CHF, congestive heart failure; CREB, cAMP response element binding protein; DCM, dilated cardiomyopathy; Epac, exchange protein activated by cAMP; ERK, extracellular signal-regulated kinase; ET-1, endothelin-1; GC, guanylyl cyclase; HF, heart failure; HFD, high-fat diet; HFpEF, heart failure with preserved ejection fraction; ICM, ischemic cardiomyopathy; IHD, ischemic heart disease; ISO, isoproterenol; KO, knockout; LIF, leukemia inhibitory factor; miR-1, microRNA-1; NE, norepinephrine; NFAT, Nuclear factor of activated T cells; NO, nitric oxide; NRCM, neonatal rat cardiomyocyte; NRVM, neonatal rat ventricular cardiomyocyte; PE, phenylephrine; PI4P, phosphatidylinositol 4-phosphate; PKA, protein kinase A, PKC, protein kinase C; PKG, protein kinase G; PLC, phospholipase C; SERCA2a, sarco(endo)plasmic reticulum calcium ATPase; SHR, spontaneously hypertensive rat; shRNA, short hairpin RNA; siRNA, short interfering RNA; Sirt1, Sirtuin 1; TAC, transverse aortic constriction; TG, transgenic mice.

#### 3.3.1. cAMP-selective PDEs.

PDE4 is known to have multiple isoforms with distinct subcellular localization in cardiomyocytes, resulting in the tight control of cAMP-dependent events within nanodomains. This diversification has led to divergent reports on the expression and function of PDE4 in cardiac hypertrophy and HF. PDE4D isoforms are associated with the cardiac βAR signaling and prevent catecholamine-induced cardiac hypertrophy. PDE4 activation by overexpression of the carboxy-terminal portion of the UCR1 domain attenuates hypertrophy in response to chronic βAR stimulation by specifically inhibiting nuclear PKA activity in cardiomyocytes (287). The PDE4D5 recruitment to β_2_AR by β-arrestin prevents activation of EPAC1 and CaMKII and the prohypertrophic effects of the receptor stimulation. Inhibition of PDE4D also leads to activation of CaMKII (88), promoting maladaptive gene expression in cardiomyocytes (297). Dissociation of the PDE4D5-β-arrestin2 complex allows the recruitment of Epac1 to β_2_AR and induces a switch from β_2_AR nonhypertrophic signaling to a β_1_AR-like prohypertrophic signaling cascade (163). PDE4 inhibitors also promote cardiac hypertrophy by enhancing a local Golgi β_1_AR-cAMP-dependent activation of the Epac-PLCε pathway (275). Meanwhile, PDE4D3 is part of a nuclear envelope mAKAPβ complex that contains PKA, Epac, and ERK5 and regulates cardiomyocyte hypertrophy through PKA and ERK5 (46, 155, 156). This local pool of PDE4D3 controls the β_1_AR-induced cAMP signaling in the nucleus for PKA regulation of hypertrophic and apoptotic gene expression (176, 298). Overexpressing catalytically inactive mutant PDE4D3 results in cardiomyocyte hypertrophy (278). These findings suggest that cardiac PDE4D protects against catecholamine-induced cardiac hypertrophy.

However, the control of cAMP-regulated hypertrophy by PDE4 is more intricate than previously appreciated. For example, in the same PDE4D3-mAKAPβ complex, inhibition of PDE4 with rolipram also blocks ERK5 activation-enhanced cardiomyocyte cell size (155). Moreover, the small heat shock protein 20 (HSP20) sequesters PDE4D isoforms (299). PDE4D regulates a specific cAMP pool that controls the PKA phosphorylation of HSP20, through which the phosphorylated HSP20 protects against hypertrophic response triggered by chronic β-agonist administration (299). Cardiac PDE4D expression is increased in a mouse model of type 1 diabetes (300). PDE4 activity is increased in an animal model of salt-induced HF and contributes to the development and exacerbation of HF (285). Recent studies have observed increased cardiac PDE4D5 expression in high-fat diet-fed mice and human diabetes-associated HF (83, 301). PDE4D inhibition by rAAV9 injection or the pharmacological inhibitor roflumilast shows protection against cardiac hypertrophy and dysfunction through promoting cAMP, cAMP response element binding (CREB), and NAD-dependent deacetylase sirtuin 1 signaling for SERCA2a expression (83). The increased SERCA2a expression, in turn, downregulates microRNA 1 (miR-1) and inhibits miR-1-targeted hypertrophy-associated genes (83). These findings suggest that cardiac PDE4D upregulation can be deleterious.

Moreover, studies show that the expression of cardiac PDE4D isoforms is either increased or decreased based on disease models (83, 284, 301, 302). The expression and activity of PDE4A and PDE4B are reportedly decreased in failing and hypertrophic hearts (169, 284, 288). A recent study has uncovered that moderate overexpression of PDE4B blunts β-adrenergic response and maladaptive remodeling in HF induced by chronic isoprenaline infusion or TAC, whereas a higher-level overexpression of PDE4B leads to maladaptive remodeling (288). The results also support the concept that the effects of cAMP on hypertrophy differ depending on the cAMP location and concentration (155). Overall, these studies report the bidirectional roles of PDE4 inhibitors in the myocardium, indicating that the effects of PDE4 isoforms may depend on their expression level and subcellular localization, as well as different etiologies and the progression stages of HF.

#### 3.3.2. cGMP-selective PDEs.

Numerous PDE isozymes involved in the cGMP-PKG signaling pathways are implicated in the regulation of cardiac hypertrophy (7, 9, 303), in which inhibiting cGMP-hydrolyzing PDEs can benefit individuals with HF. Two stimulatory pathways, NOS and NP, promote cGMP signals (304). In chronic pressure-induced hypertrophy, the NO-sGC-dependent cGMP declines, whereas the NP-derived cGMP rises (305). PDE5A localizes to cardiomyocyte Z bands in the normal heart and preferentially regulates cGMP through adrenergic stimulation of NOS3 (148). PDE5 expression is generally low in the heart with low activity in myocytes (88) but is elevated in ventricular hypertrophy in mice, rats, and humans (89, 284, 285, 288, 301–303). Myocardial oxidative stress increases PDE5 expression in the failing heart, whereas reducing oxidative stress by M40401 treatment attenuates PDE5 expression (303). Moreover, PDE5 is translocated from sarcomeres to a dispersed distribution in TAC hearts (305), which shifts the cGMP hydrolytic activity from the NOS3-sGC pathway to the NP signaling pathway. Studies have supported the protective actions of PDE5 inhibitors on cardiac hypertrophy by promoting cGMP-PKG signaling (285, 302, 303, 306). Suppressing myocyte PKG activity exacerbates pressure-overload remodeling and abrogates the antihypertrophy effects of the PDE5 inhibitor sildenafil (305, 307), highlighting PKG as a critical downstream effector in preventing pathological remodeling. PDE5 inhibition also reduces pathological hypertrophy by activating the regulator of G protein signaling and inhibiting the transient receptor potential canonical channel (282, 283, 306). The antihypertrophy actions of PDE5 inhibitors are also associated with the inhibition of multiple hypertrophy-signaling pathways, including the calcineurin-nuclear factor of activated T cells (NFAT), PI3K-Akt, p38, and ERK1/2 MAPK signaling pathways (285, 286), myocardial Na^+^/H^+^ exchanger (308), and PKG-1α oxidation (309). Additionally, sildenafil attenuates TAC-induced cardiac hypertrophy and ameliorates T-tubule remodeling, suggesting another potential mechanism underlying the therapeutic benefits of PDE5 inhibitors in cardiac hypertrophy (310).

PDE9A is expressed in hearts from mice and humans and is upregulated in myocardial hypertrophy and HF (120, 311). In contrast to PDE5 involvement in the NO-stimulated cGMP, PDE9A preferentially regulates the NP-induced cGMP (120). Because of different subcellular distributions, PDE5 and PDE9 target different intracellular cGMP pools in myocytes. In the TAC model of HF, PDE9A deficiency ameliorates cardiac hypertrophy and ventricular function (120). Selective inhibitors such as PF-9613, CRD-733, and C33(S) augment the antihypertrophic and antifibrotic NP-cGMP pathways (312–314) and protect against pathological cardiac hypertrophy responses to sustained neurohormone stimulation and pressure overload (120, 315, 316). Unlike PDE5A, PDE9A inhibition can reverse preestablished cardiac hypertrophy independent of NOS activity (120). Thus, PDE9A inhibitors may have greater effectiveness than PDE5A inhibitors for treating cardiac hypertrophy when NO production is low. Moreover, inhibition of neprilysin augments the levels of NPs when used together with PDE9 inhibitors and may have additional beneficial hemodynamic and renal effects in HF compared with either PDE9 or neprilysin inhibition alone (317).

#### 3.3.3. Dual-Substrate PDEs.

Although PDE1A plays a crucial role in cardiac hypertrophy by regulating cGMP degradation, evidence suggests that PDE1C is also involved in regulating cardiomyocyte hypertrophy by regulating cAMP degradation (23, 26). PDE1A upregulation has been observed in the hearts of various pathological hypertrophy animal models and isolated cardiomyocytes treated with neurohumoral stimuli such as angiotensin II (Ang II) and ISO (23). PDE1 inhibitors or PDE1A short hairpin RNA can rescue the reduced cGMP levels in cardiomyocytes and reverse myocyte hypertrophy, suggesting that the antihypertrophic effects of PDE1A inhibition are mediated through cGMP-PKG signaling (306). Conversely, PDE1C is highly expressed in cardiomyocytes and upregulated in mouse and human failing hearts (26). Deletion of PDE1C significantly alleviates cardiac hypertrophy and dysfunction in mice induced by TAC. The antihypertrophic effects of PDE1C deficiency or inhibition largely depend on cAMP-PKA and PI3K-AKT signaling but are not coupled to cGMP signaling (26). Studies have identified a protein complex linking PDE1C to transient receptor potential canonical channel type 3 (Trpc3) and type A2 adenosine receptors (A2Rs), in which PDE1C inhibition enhances the A2R-cAMP-induced antiapoptotic protection (167). Further investigation into the differential mechanisms of PDE1A and PDE1C in cardiac hypertrophy and HF will aid in developing PDE1 isozyme-selective inhibitors to achieve specific pharmacological effects.

PDE2 is less abundant in cardiomyocytes than in fibroblasts and ECs under normal conditions (44). PDE2 expression and cAMP-hydrolyzing activity significantly increase in left ventricular myocardium from patients with terminal HF and in various experimental animal models. The upregulated PDE2 desensitizes cardiomyocytes against acute βAR stimulation (42, 43). PDE2A is also upregulated in inducible pluripotent stem cell-derived cardiomyocytes from patients with familial hypertrophic cardiomyopathy (304). However, in left ventricle (LV) tissues from aortic stenosis patients with myocardial hypertrophy and preserved cardiac function, PDE2 expression remains normal (42). Whereas β-adrenergic stimulation activates the cAMP-Epac-PLCε pathway for cardiac hypertrophy (275), PDE2 limits cAMP-PKA-dependent inhibition of the PLCε hypertrophic pathway. PDE2 also controls a separate PKG-dependent pathway that indirectly inhibits the PLCε hypertrophic pathway (275). However, overexpressing PDE2A yields inconsistent observations on cardiac hypertrophy. One study shows that overexpression of PDE2A is sufficient to induce cardiomyocyte hypertrophy in vitro and in vivo (278). In another report, PDE2A overexpression blunts norepinephrine-induced cellular hypertrophy with a marked decrease in cAMP levels (42). These conflicting results could be due to differential regulation of cAMP and cGMP and the cross talk between cGMP and cAMP that depends on the cGMP concentrations and various stressors. cGMP, at a low concentration, inhibits PDE2A, thereby increasing a local pool of cAMP. A high cGMP concentration activates PDE2 by binding to the regulatory domain of PDE2, allowing a cGMP-mediated decrease in cAMP signaling (305). Nevertheless, PDE2 inhibitors have shown antihypertrophic effects in the heart. In the pressure-load-induced right or left ventricular hypertrophy models, PDE2 inhibition promotes NO-GC-cGMP signaling and PKG-mediated antihypertrophic effects and improves cardiac function (43, 278, 307). Another study shows that the PDE2 inhibitor significantly reduces TAC-induced cardiac hypertrophy by generating a local pool of cAMP that enhances a subset of PKA type II-mediated phosphorylation of NFAT and prevents the NFAT-mediated hypertrophic gene expression (278). Overall, PDE2A plays a pathophysiological role in heart diseases; inhibition of PDE2 in cardiomyocytes might be beneficial to counteract pathological remodeling induced by pressure overload (43, 278, 307). Further studies are needed to determine whether PDE2 inhibition or activation provides therapeutic effects in HF of various etiologies.

Studies have reported inconsistent PDE3 expression and activity in cardiac hypertrophy and HF (7). Early studies show no change in PDE3 activity in human failing hearts (282, 283). However, others report increased activity and expression of PDE3 in the TAC mouse model of HF, streptozotocin-induced diabetic cardiomyopathy, and salt-induced hypertension, hypertrophy, and HF (98, 285, 286, 300). Additional studies have shown that PDE3 activity and PDE3A expression are decreased in mouse and rat hypertrophic and failing hearts induced by pressure overload (58, 284), in pacing-induced chronic HF dogs (58, 284, 302, 308), and in human HF (302). Moreover, Ang II and ISO induce a sustained reduction of PDE3A expression in cultured rat neonatal cardiomyocytes (58). These discrepancies may be due to differences in species and underlying causes of HF. Nevertheless, PDE3 inhibitors have been shown to induce cardiac hypertrophy by increasing a cAMP pool that promotes the Epac-PLCε-PKC-dependent hypertrophic growth (275). The cAMP generated via inhibition of PDE3 has prohypertrophic effects via activation of a subset of PKA that is not anchored to AKAPs (278). Moreover, PDE3A2 isoform operates in a nuclear nanodomain that involves mothers against decapentaplegic homolog 4 (SMAD4) and histone deacetylase 1 (HDAC-1). Inhibition of PDE3 results in increased HDAC-1 phosphorylation, which reduces its deacetylase activity and increases gene transcription and cardiomyocyte hypertrophic growth (309). PDE3 inhibition also worsens chamber stiffness and increases collagen type I deposition induced by chronic βAR stimulation in rats (310). These mechanisms might contribute to the detrimental effects of long-term PDE3 inhibition, suggesting that increasing PDE3A activity could be a compensatory mechanism for cardiac hypertrophy during the development of HF. PDE3A but not PDE3B ablation protects against TAC-induced adverse ventricular remodeling via reducing p38 MAPK and calcineurin-NFAT activation (286). Moreover, PDE2, PDE3, and PDE4 are localized at distinct subcellular sites and uniquely modulate the cAMP response in intact neonatal rat ventricular myocytes (45, 311). βAR activation and other pathological stimuli can generate spatially distinct pools of cAMP with opposing effects on myocyte size (278).

HF has been associated with changes in expression and localization of PDEs. Yet it remains unclear how one PDE compensates for changes in the activity of another PDE during signaling modulation. Future work will focus on better defining the cAMP-cGMP signaling network and alternations. Additional efforts may be sought to simulate the complex network changes during HF with computation approaches, which may lead to new insights into the pathophysiology of HF and the development of new HF therapies.

### 3.4. PDE in Ischemic Cardiomyopathy

Compared with hypertrophic cardiomyopathy induced by either hormones or pressure overload, MI presents a fundamentally different etiology in the myocardium. The role of PDEs appears to vary, even opposing, in the pathogenesis of ischemic heart diseases compared with hypertrophic HF. Here, we review the function of PDE isozymes in cell death and ischemic cardiomyopathy (TABLE 3).

**Table 3.** The roles of individual PDE isoforms in cardiac ischemia injury

PDE Family	Isoform	Variants	Model in vivo	Model in vitro	Genetic Intervention in vivo	Genetic Intervention in vitro	Inhibitors Used	Positive ↑Negative ↓	Signaling Pathway	Reference
PDE2			Ex vivo perfused mouse heart-reperfusion injury				BAY 60-7550	↓	↓Arrhythmic events	(271)
	PDE2A			Ionomycin-NRCM		PDE2A-siRNA	BAY 60-7550	↓	↑Drp1 phosphorylation ↑mitochondria dynamics	(48)
		**PDE2A3**	**LAD ligation mice-MI**		**PDE2A3-TG**			**↓**	**↑Ca^2+^ homeostasis ↓contractile dysfunction ↓cardiac arrhythmias**	**(218)**
PDE3										
				**NRCM per se**			**Milrinone, cilostamide**	**↑**	**↑ICER**	**(58)**
			**Ischemia-reperfusion dog**				**Milrinone, olprinone**	**↑**	**↑cAMP-PKA-p38**	**(318)**
			Ischemia-reperfusion rat				Olprinone	↓	↑PI3K-Akt **↓** mPTP	(319)
			Ex vivo perfused rabbit heart-reperfusion injury				Cilostazol	↓	↑mitoK(Ca) channels	(320)
			Ex vivo perfused rabbit heart-reperfusion injury				Milrinone	No effect		(320)
	**PDE3A**			**NRCM per se**			**Adenovirus- antisense-PDE3A**	**↑**	**↑ICER**	**(58)**
		**PDE3A1**		**Ang II/ISO-NRCM**			**Adenovirus- PDE3A1**	**↓**	**↑ICER**	**(58)**
			**Ischemia-reperfusion mice**			**Cardiac-specific overexpression of PDE3A1**		**↓**	**↓ICER**	**(321)**
	PDE3B		Ischemia-reperfusion mice			PDE3B^−/−^ mice		↓	↑Ca^2+^-activated K^+^ channels, ↓ROS	(59)
PDE4										
	PDE4B		Ischemia-reperfusion mice			PDE4B^−/−^ mice		↓	↑Cardiac microcirculation ↓inflammation	(312)
	PDE4D									
		**PDE4D3**	**LAD ligation mice-MI**			**PDE4D^−/−^ mice**		**↑**	**↑RyR2-phosphorylation ↑Ca^2+^ leakage**	**(169)**
PDE5			Ischemia-reperfusion rabbit				Sildenafil	↓	↑A(1) adenosine receptor activation	(313)
			Ex vivo perfused mouse heart-reperfusion injury				Sildenafil	↓	↑Mitochondrial K(ATP) channels	(314)

Boldface indicates that the phosphodiesterase (PDE) plays a negative role in ischemia injury. cAMP, cyclic adenosine monophosphate, Drp1, dynamin-related protein; ICER, Inducible cAMP early repressor; ISO, isoproterenol; LAD, ligation of left anterior coronary artery; MI, myocardial infarction; mPTP, mitochondrial permeability transition pore; NRCM, neonatal rat cardiomyocyte; p38, p38 mitogen-activated protein kinase; PI3K, phosphoinositide 3-kinase; PKA, protein kinase A; RyR2, ryanodine receptor 2; TG, transgenic mouse.

#### 3.4.1. cAMP-specific PDEs.

PDE4D3 has been reported to localize within the RyR2 macromolecular complex (169). Deletion of PDE4D leads to spontaneous, age-related cardiomyopathy and exacerbates MI by promoting hyperphosphorylated RyR2 and Ca^2+^ leakage (169). PDE4 binds to mAKAP to reduce nuclear PKA activity and expression of the proapoptotic factor inducible cAMP early repressor (ICER) (176). Moreover, through the negative feedback between PDE4D and CaMKII (88), inhibition or deletion of PDE4D increases CaMKII activity and ICER expression, thereby promoting myocyte apoptosis. These observations are in contrast to the role of PDE4D in cardiac hypertrophy induced by adrenergic stimulation (176, 298) and high-fat diet feeding (83, 301). Meanwhile, the expression of PDE4B but no other PDE4 subtypes increases in mouse hearts with myocardial infarction-reperfusion (MI/R) injury (312). PDE4B is detected primarily in endothelial and myeloid cells of mouse and human hearts. Deleting PDE4B alleviates infarct size and improves cardiac function after MI/R by suppressing inflammation, enhancing cardiac microcirculation, and protecting against MI/R injury (312). These observations contrast with the findings in mice with moderate overexpression of PDE4B in cardiomyocytes, in which PDE4B blunts β-adrenergic response and maladaptive remodeling in HF induced by chronic isoprenaline infusion or TAC (288). Again, these discrepancies indicate an etiology- and cell context-dependent role of PDE4 isoforms in the pathogenesis of heart diseases.

#### 3.4.2. cGMP-specific PDEs.

Cardiac-specific overexpression of PDE5 does not affect myocardial structure or function at the baseline but significantly exacerbates cardiomyocyte hypertrophy and reduces cardiac function after MI (89). When administered before ischemia and after ischemia, PDE5 inhibitors reduce infarct size and apoptosis, and postreperfusion increases contractile function and survival (313–315). Studies demonstrate that sildenafil has cardioprotective effects against MI and ischemia-reperfusion injury through multiple mechanisms, including increasing NO-cGMP signaling and PKG activation and opening mitochondrial K(ATP) channels and Ca^2+^-activated potassium channels (313, 314). Clinically, the use of PDE5 inhibitors in type 2 diabetes mellitus (T2DM) is associated with a lower risk of overall morbidity and mortality in those with a history of acute MI (316). Further evidence is required to elucidate the mechanism of PDE5 inhibitor-associated cardioprotection in myocardial ischemia.

#### 3.4.3. Dual-substrate PDEs.

In contrast to the pressure-stress hypertrophy model of HF, PDE2 activation in cardiomyocytes might benefit ischemic HF by improving Ca^2+^ homeostasis, limiting contractile dysfunction, and preventing cardiac arrhythmias (218, 271). Studies suggest that cardiomyocyte-specific overexpression of PDE2A3 has no significant cardiac hypertrophy but improves contraction force and protects against arrhythmia after MI (218, 271). In mice with myocyte-targeted PDE2A3 overexpression, the resting heart rate is reduced, the catecholamine-stimulated arrhythmia is abated, and the cardiac function after MI is improved over littermate control mice (42, 271). In contrast, reports show that inhibition of PDE2A2 affects mitochondria dynamics and protects from apoptotic cell death by enhancing the cAMP-dependent phosphorylation of dynamin-related protein 1 (48). These studies reveal the complex roles of PDE2A isoforms in heart diseases, including the cyclic nucleotide substrate selectivity, the source and location of the substrate, and the etiologies of cardiac diseases (317). Thus, the role of PDE2 depends very much on the ambient conditions in vivo, which may alter the location and balance between cGMP and cAMP and determine whether blocking or activating PDE2 is beneficial.

A decrease of PDE3A with consequent ICER induction is a critical event in Ang II- and ISO-induced cardiomyocyte apoptosis and may contribute to the development of HF (58). Reduction in PDE3A has been shown to upregulate ICER, a negative regulator of B cell lymphoma 2 (Bcl-2)-associated agonist of cell death and a potent proapoptotic factor in cardiomyocytes (322). A decreased PDE3 activity may be related to the loss of cardiomyocytes and subsequent replacement with fibrous tissue in end-stage HF (323). In contrast, mice with cardiac-specific overexpression of PDE3A1 are protected against myocardial ischemia-reperfusion, with a reduced infarct size associated with decreased expression of ICER, increased expression of Bcl-2, and resistance to cardiomyocyte apoptosis (321, 324). However, studies have also reported beneficial roles of PDE3 inhibition in myocardial ischemia-reperfusion, like those reported in cardiomyopathy induced by pressure overload (286). PDE3 inhibition decreases infarct size in canine hearts after myocardial ischemia-reperfusion via a mechanism dependent on the cAMP-PKA-p38 MAPK pathway (318). PDE3 inhibitors, like olprinone and cilostazol, also protect against ischemia-reperfusion injury by inhibiting mitochondrial permeability transition pores and direct activation of mitochondrial potassium channels (319, 320). Moreover, in a model of acute ischemia-reperfusion, PDE3B^−/−^ mice but not PDE3A^−/−^ mice had reduced infarct size (59). The cardioprotective effects of PDE3B deficiency depend on cAMP-PKA signaling, which potentiates the opening of Ca^2+^-activated K^+^ channels and decreases reactive oxygen species (ROS) formation by mitochondrial fractions (59). These reported opposite effects of PDE3 in the myocardium may be multifactorial. For instance, the expression level and activity of PDE3 could be isoform dependent: PDE3A and PDE3B may play opposite roles during ischemia-reperfusion, which may be linked to their differential localization and the control of discrete cAMP pools in cardiomyocytes (59). The function of overexpressing PDE3 may be two phased: moderate PDE3 overexpression is cardioprotective, whereas excessive PDE3 is detrimental to the heart (321). Although cAMP is essential for cardiomyocyte survival, chronic cAMP elevation can lead to proapoptotic and antiapoptotic pathways during cardiac remodeling and dysfunction (273). Similarly, PDE3 inhibitors have been used for acute HF management, but chronic usage has been associated with increased mortality in human patients.

### 3.5. PDE in the Regulation of Mitochondria and Cardiac Metabolism

Mitochondria are the source of cellular energy production. Mitochondrial dysfunction is associated with the development of numerous cardiac diseases, such as ischemia-reperfusion injury, cardiac hypertrophy, and HF, due to the uncontrolled production of reactive oxygen species (ROS). cAMP is rate limiting for matrix Ca^2+^ entry via the Epac1-dependent mitochondrial Ca^2+^ uniporter and, as a result, abrogates mitochondrial permeability transition (325). The latter effects protect against cardiomyocyte cell death and apoptosis (325). Studies have highlighted that PDE2 accounts for the largest mitochondrial cAMP-degrading activity compared with PDE3 and PDE4 in adult rat ventricular myocytes (326). PDE2 is also associated with critical signaling pathways modulating cardiomyocytes’ energetic capacities and apoptosis. In a study on rat cardiomyoblasts, 17β-estradiol reduced mitochondrial cAMP levels via cGMP-mediated stimulation of PDE2, decreasing cytochrome oxidase activity and mitochondrial membrane potential (327). Upon PDE2 inhibition with BAY60-7550, elevated mitochondrial cAMP levels are implicated in increased oxygen consumption, mitochondrial membrane potential, and ATP production (325, 328). Moreover, PDE2 inhibition limits sepsis-induced myocardial dysfunction and improves mitochondrial respiration in septic cardiac fibers (329). Furthermore, PDE2A2 is the only isoenzyme reported to be localized within subsarcolemmal mitochondria associated with the mitochondrial inner membrane and regulates mitochondria dynamics in myocytes (47). PDE2A2 inhibition affects mitochondrial elongation and increases the transmembrane potential and, thereby, resistance to proapoptotic stimuli in neonatal rat ventricular myocytes (48). These changes are mediated by cAMP-PKA-dependent phosphorylation of dynamin-related protein 1, which diminishes mitochondria fission, protecting the cardiomyocytes against apoptotic cell death (48). Mice with cardiomyocyte-specific PDE2 overexpression exhibit faster mitochondrial membrane potential loss and mitochondrial swelling (47). Additionally, PDE4 has been implicated in mitochondria regulation: a potent PDE4 inhibitor activates AMP-activated protein kinase and Sirt1 to induce mitochondrial biogenesis in myotubes (330).

### 3.6. PDE in Cardiac Fibroblasts

Myocardial fibrosis is a common pathological change associated with cardiac diseases and is characterized by an imbalance of extracellular matrix (ECM) deposition, leading to cardiac stiffness and severe HF. Cardiac fibroblasts, the major cell type in the heart, are the primary source of ECM deposition in cardiac fibrosis. In response to various pathological stresses such as MI and pressure overload, cardiac fibroblasts are stimulated to proliferate and differentiate into myofibroblasts, producing ECM deposition and fibrosis (331). Cyclic nucleotide signaling has antifibrotic properties by negatively regulating pathways associated with myofibroblast transformation, proliferation, and expression of ECM molecules in injured or stressed hearts (332, 333). Evidence has suggested that PDE expression and activity are involved in various cellular processes in myocardial fibrosis in MI and hypertrophic cardiomyopathy (25, 44). Among the PDEs, PDE1, 2, 3, 4, 5, and 10 families have been shown to modulate profibrotic function in cardiac fibroblasts (25, 43, 129). Inhibition of these PDEs attenuates cardiac fibrosis in various heart disease models.

#### 3.6.1. cAMP-specific PDEs.

The expression of PDE4D has recently been observed in cardiac fibroblasts (83). PDE4D5 is upregulated by insulin in cardiac fibroblasts in vitro and in mouse hearts after high-fat diet feeding (83). PDE4D5 overexpression enhances transforming growth factor β1 (TGF-β1) signaling, reducing miR-1 expression and increasing collagen deposition in cardiac fibroblasts and mouse hearts after high-fat diet feeding (83). Both PKA and Epac, the primary cAMP effectors, inhibit the TGF-β1-induced synthesis of collagen and DNA by fibroblasts (334). However, PKA and Epac have opposing effects on fibroblast migration and morphology. PDE4 inhibition promotes fibroblast migration at lower concentrations but decreases migration at higher concentrations, which is probably correlated to those elicited by activation of Epac1 and PKA, respectively (334). These studies imply that PDE4 inhibition might have dose differences in its effect on cardiac fibroblast proliferation and migration.

#### 3.6.2. cGMP-specific PDEs.

PDE5 has been identified in mouse cardiac myofibroblasts, and PDE5 activity contributes to approximately half of the total cGMP-hydrolyzing activity of myofibroblasts (97). Inhibition of PDE5 with sildenafil significantly reduces ISO-, Ang II-, and TAC-induced fibrosis in mouse hearts (100, 310, 335). Inhibition of PDE5 attenuates cardiac fibrosis by reducing CREB binding protein 1 recruitment to Smad transcriptional complexes by activating CREB in cardiac fibroblasts (100). Another study reveals that PDE5A inhibition with adenoviral short hairpin RNA partially reduces fibrosis in infarcted hearts through activation of the Akt signaling pathway and reduction of inflammatory cytokines (336). The mechanisms underlying the protective effects of PDE5 inhibition on cardiac fibrosis merit further investigation.

#### 3.6.3. Dual-substrate PDEs.

The Ca^2+^-CaM-stimulated PDE1A isoform is the main cAMP-hydrolyzing enzyme expressed in cardiac myofibroblasts from mouse, rat, and human fibrotic hearts. PDE1A is highly induced in cardiac fibroblasts activated by profibrotic stimuli such as TGF-β and Ang II and in vivo within fibrotic regions of rodent and human failing hearts (25). Inhibiting PDE1A with a PDE1-selective inhibitor or short hairpin RNA reduces myofibroblast activation in vitro and attenuates interstitial fibrosis in mouse hearts (25). Mechanistic studies reveal that PDE1A controls unique spatial and temporal cAMP and cGMP dynamics, predominantly in perinuclear and nuclear regions of cardiac fibroblasts, which may be implicated in the differential regulation of stress-responsive genes (25). Both cAMP-EPAC and cGMP-PKG are responsible for PDE1A-mediated nuclear fibrotic gene activation (25). Targeting PDE1 with vinpocetine inhibits pathological cardiac fibrosis in mice in response to chronic Ang II infusion (281). Conversely, PDE1C expression is upregulated in mouse and failing human hearts, and it is highly expressed in cardiomyocytes but not in fibroblasts. Deletion of PDE1C significantly attenuates cardiac remodeling and dysfunction induced by TAC, including myocardial hypertrophy and cardiac fibrosis (26). Studies have found that conditioned medium taken from PDE1C-deficient cardiomyocytes can partially attenuate TGF-β-stimulated cardiac fibroblast activation, suggesting that PDE1C in myocytes likely regulates secreted factors that are important for fibroblast activation (26). PDE1 inhibition may serve as an effective strategy to alleviate fibrosis and cardiac dysfunction.

PDE2, a dual-substrate PDE that hydrolyzes both cAMP and cGMP, is abundantly expressed in neonatal rat cardiac fibroblasts (44). Overexpression of PDE2 in cardiac fibroblasts enhances basal cAMP degradation, induces fibroblast conversion to myofibroblast even in the absence of exogenous profibrotic stimuli, and drives the stiffness of fibroblast-derived connective tissues (44). PDE2 suppresses the increase in cAMP in response to ISO in cardiac fibroblasts and promotes myofibroblast formation and fibrosis (44, 337). Meanwhile, cardiac fibroblasts can produce large quantities of NO in response to interleukin-1β (IL-1β) stimulation and the consequent induction of inducible NOS2, which stimulates the NO-sGC-cGMP cascade to activate PDE2 and depress cAMP accumulation (337). A selective pharmacological inhibitor of PDE2, BAY 60-7550, reverses the development of cardiac fibrosis induced by pressure overload (43). Despite persistently depressed cAMP levels, ANP- and sodium nitroprusside-mediated cGMP synthesis completely prevents PDE2 overexpression-induced fibroblast conversion (44). Furthermore, parallel studies in animals deficient in either NO-sensitive sGC-1α (sGC-1α^−/−^) or natriuretic peptide-responsive GC-A (GC-A^−/−^) show that the beneficial effects of PDE2 inhibition are maintained in GC-A^−/−^ mice but absent in sGC-1α^−/−^ mice. These data indicate that PDE2 inhibition promotes NO-sGC-cGMP signaling to protect cardiac structure and function (43).

The PDE3 inhibitor milrinone attenuates TAC-induced hypertrophy and interstitial fibrosis, macrophage infiltration, and activation of p38 MAP kinase in mice (286). Whole body genetic deletion of PDE3A, but not PDE3B, provides similar protection against TAC-induced adverse ventricular remodeling (286). Milrinone does not provide additional protection to PDE3A^−/−^ mice, suggesting that PDE3A contributes to cardiac fibrosis induced by pressure overload (286). Moreover, milrinone suppresses Ang II-induced transactivation of fibroblasts to myofibroblasts, consistent with reduced ventricular fibrosis in TAC mice with PDE3 blockade and PDE3A ablation. Cilostazol, a selective PDE3 inhibitor with antiplatelet, antimitogenic, and vasodilating properties, also attenuates Ang II-induced cardiac fibrosis in mice by activating the cAMP-PKA pathway (338).

PDE10A, another dual cAMP and cGMP esterase, is markedly upregulated in failing hearts (129). PDE10A deficiency or inhibition of PDE10A with selective inhibitor TP-10 attenuates TAC or Ang II infusion-induced cardiac fibrosis (129). TP-10 treatment elevates cAMP and cGMP levels and reduces TGF-β-stimulated cardiac fibroblast proliferation, migration, and ECM synthesis (129).

Collectively, pharmacological inhibition of PDEs such as PDE1, PDE2, PDE3A, PDE4D, PDE5, and PDE10A is currently being considered as a potential therapeutic approach for the treatment of heart pathologies associated with cardiac fibrosis.

### 3.7. PDE in Immune Cells and Inflammation

Growing evidence implies that inflammation contributes to cardiovascular diseases, including atherosclerosis, myocardial infarction, and HF. Patients with decompensated HF show marked systemic inflammation and increased production of oxygen free radicals. Studies have shown that the acute inflammatory response induced by the innate immune system is required for tissue repair during injury and is cardioprotective (339). However, sustained activation of inflammatory signaling contributes to the process of LV remodeling, including cardiomyocyte hypertrophy, activation of collagenolytic matrix metalloproteinases, myocardial fibrosis, and progressive myocyte apoptosis (340). Various types of immune cells are implicated in myocardial inflammation, including neutrophils, macrophages, eosinophils, mast cells, natural killer cells, T cells, and B cells. Immune cells coordinate the responses of cardiomyocytes and noncardiomyocytes during maladaptive remodeling (339). Immune cells modulate not only cardiomyocyte function directly but also injury responses involving scar formation and interstitial fibrosis.

Targeting PDEs with small-molecule inhibitors is a promising therapeutic for chronic inflammatory disorders. Despite extensive knowledge of PDE expression and regulation in immune cells such as macrophages and T cells (341), research into the role of PDEs in cardiac inflammatory responses is limited.

PDE4B is expressed in macrophages (312). PDE4B inhibition or deletion suppresses inflammation, enhances cardiac microcirculation, and protects against MI/R injury. Mechanistically, PDE4B facilitates neutrophil-EC interaction by promoting the release of proinflammatory cytokines and PKA-mediated cell adhesion molecule expression (312). Similarly, inhibition of PDE4 protects against doxorubicin-induced cardiomyopathy in rats by reducing oxidative stress-mediated inflammatory reactions (342, 343). Although the PDE7 inhibitor BRL50481 has only a modest inhibitory effect on proinflammatory cells, it acts additively with other cAMP-elevating drugs, such as PDE4 inhibitors, especially when the PDE7A1 isoform is upregulated (344). It is hypothesized that dual inhibitors of PDE4 and PDE7 may offer a therapeutic choice in HF with inflammation and exhibit a lower propensity to cause adverse side effects by PDE4 inhibitors.

PDE5 inhibitor rescues left ventricular dysfunction and cardiac remodeling in Ang II-induced HF accompanied by reducing inflammatory immune response (345). PDE5A knockout suppresses inflammation by downregulating adhesion molecules in cardiac rupture following MI (346). Treatment with the PDE5 inhibitor tadalafil ameliorates circulating inflammatory cytokines and chemokines in a diabetic animal model while improving fasting glucose levels and reducing infarct size after ischemia-reperfusion injury in the heart (347). Patients with both hyperglycemia and inflammation have poor outcomes. Sildenafil reduces C-X-C motif chemokine ligand 10 levels in human cardiomyocytes and T2DM subjects (348). Moreover, in a clinical trial of diabetic cardiomyopathy, tadalafil reduces low-grade chronic inflammation and improves cardiac function (349). These studies underscore the possibility that PDE5 inhibitors can be developed as a pharmacological tool to control inflammation in diabetic cardiomyopathy.

## 4. PDEs IN VASCULAR BIOLOGY AND DISEASES

### 4.1. Vascular Structure and Function

**FIGURE 8.**
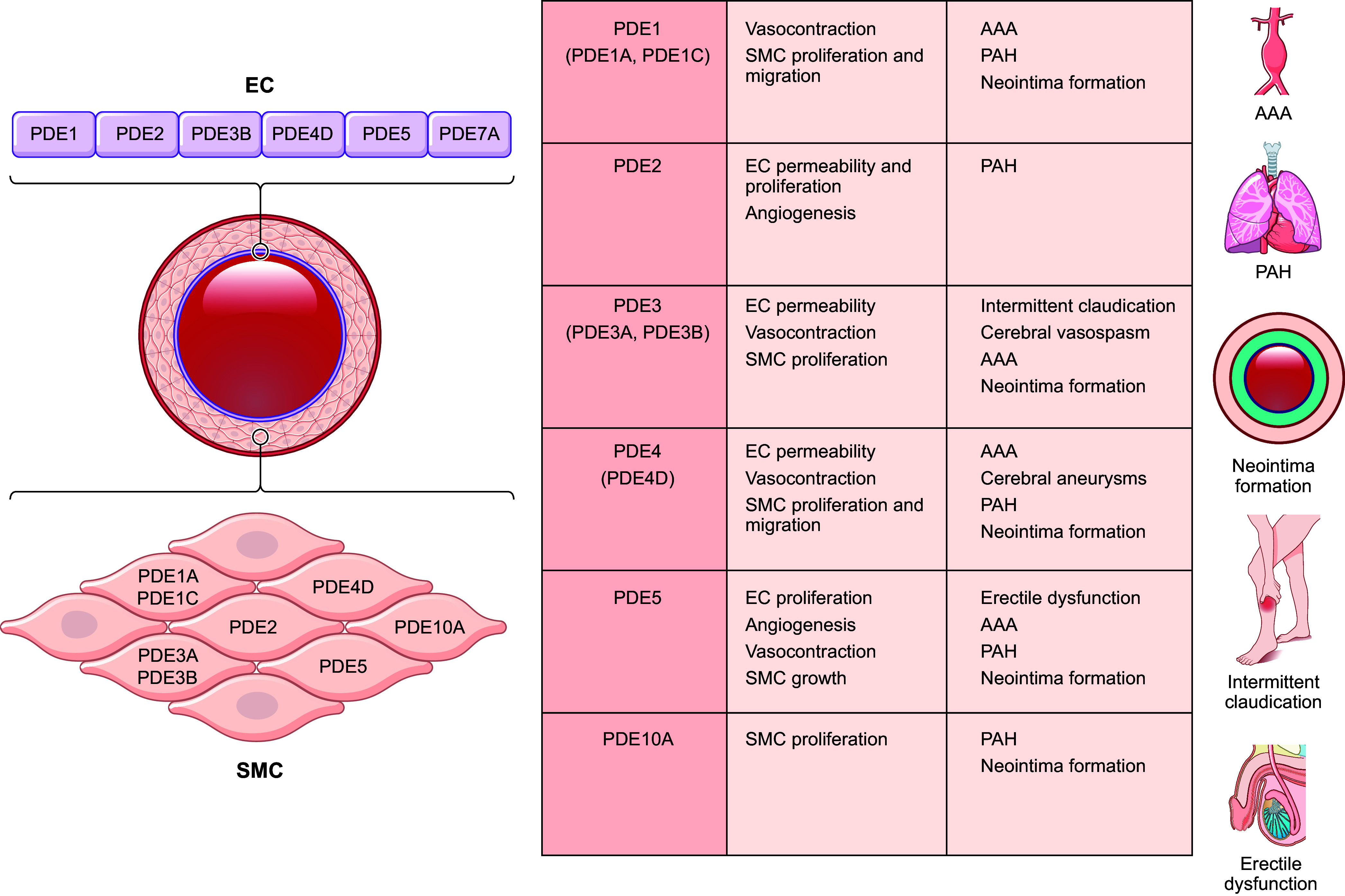
Schematic illustration of the expression and function of phosphodiesterases (PDEs) in the arterial vessel and their involvement in vascular diseases. AAA, abdominal aortic aneurysm; EC, endothelial cell; PAH, pulmonary arterial hypertension; SMC, smooth muscle cell.

Blood vessels (arteries, veins, and capillaries) are vital in maintaining blood circulation by delivering oxygen and nutrients to and removing wastes from the body. Blood vessels are categorized into different types in size, structure, and function. Malfunctions of different blood vessels are associated with distinct vascular diseases. Arteries, including elastic arteries, muscular arteries, and arterioles, carry oxygenated blood away from the heart to various organs and tissues. The size and wall thickness of the arteries decrease as their distance from the heart increases. The elastic arteries have the thickest walls and withstand the highest pressure of blood pumped out by the heart. Vessel wall degeneration of elastic arteries causes dissections and aneurysms (350). Atherosclerosis, characterized by plaque buildup in the vessel wall, primarily occurs in the middle- to large-sized arteries (351). Hypertension (high blood pressure) is often related to abnormalities in small arteries, particularly arterioles between arteries and capillaries (352). Veins usually have thinner walls and carry deoxygenated blood with low pressure from tissues to the heart. Capillaries, the smallest vessels in the body, mediate the gas and nutrient exchange between the blood and tissues. This review mainly focuses on vascular diseases associated with arteries (FIGURE 8).

Arteries have three layers containing different cell types and performing distinct functions. The innermost layer, also known as intima, is a single-cell layer with ECs that line the surface of blood vessels. The endothelial layer is crucial in sensing biological and mechanical signals in blood and regulating vascular reactivity and permeability. The middle layer, also known as media, mainly contains SMCs and matrixes such as elastin and collagen. SMCs are critical in vasoconstriction or vasodilation. The outer layer, also known as adventitia, is important in maintaining the integrity and elasticity of the arterial wall. Adventitia mainly comprises fibroblasts and connective tissues (such as collagen), and fibroblasts are responsible for producing connective tissue.

### 4.2. PDE in Vascular Reactivity and Hypertensive Diseases

**FIGURE 9.**
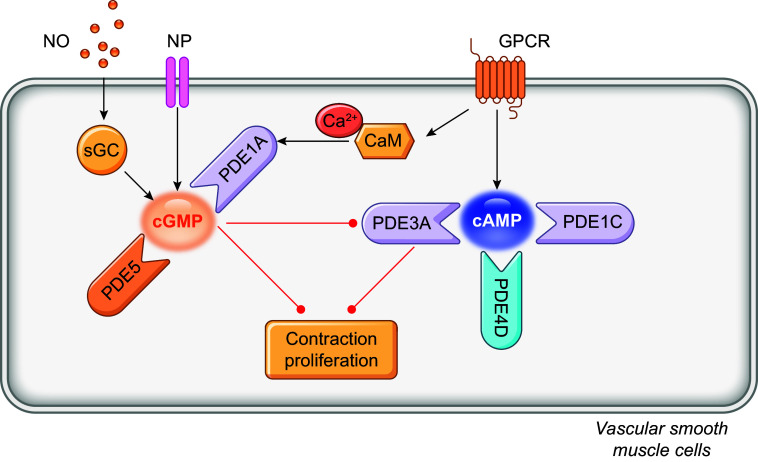
Schematic illustration of phosphodiesterases (PDEs) in smooth muscle cells. The cartoon highlights the regulation of cyclic adenosine monophosphate (cAMP) and cyclic guanosine monophosphate (cGMP) by PDEs in smooth muscle cells to control vessel constriction and proliferation. CaM, calmodulin; GPCR, G protein-coupled receptor; NO, nitric oxide; NP, natriuretic peptide; sGC, soluble guanylyl cyclase.

In response to various biological and environmental factors, blood vessels can change their diameter, which is essential for regulating blood flow to distribute nutrients and oxygen throughout the body and maintaining blood pressure. The cellular and molecular mechanisms underlying vascular reactivity regulation are complex, involving multiple pathways including cyclic nucleotide signaling. Both ECs and SMCs play crucial roles in regulating vascular reactivity. ECs regulate vascular tone by releasing various substances, including vasodilators, such as NO and prostacyclin (PGI_2_), and vasoconstrictors, such as endothelin (ET) and thromboxane. SMC contraction and relaxation directly control the vessel diameter, which can be regulated by *I*_Ca_ levels. Increased Ca^2+^ activates myosin light chain kinase (MLCK) that phosphorylates the myosin light chains (MLC) of the myosin molecules, which triggers myosin binding to actin filaments and SMC contraction (353). cAMP and cGMP are key mediators of SMC relaxation. PGI_2_ can induce cAMP elevation and activate PKA in SMCs, which leads to SMC relaxation by PKA-mediated phosphorylation and activation of MLC phosphatase (MLCP) that dephosphorylates MLC (354, 355). PKA can also increase the activity of the potassium channels to hyperpolarize SMCs, which indirectly reduces Ca^2+^ entry into SMCs (356, 357). However, a series of recent studies show that glucose can promote ATP-dependent activation of purinergic receptor 11 to induce a discrete pool of cAMP, which can promote PKA activation of LTCC to increase SMC constriction (358–360). Conversely, NO can induce cGMP elevation and activate PKG in SMCs, leading to SMC relaxation via different mechanisms (361), e.g., PKG activates MLCP, like PKA (362); PKG phosphorylates inositol 1,4,5-trisphosphate (IP_3_) receptors and decreases Ca^2+^ release from the sarcoplasmic reticulum (363); and PKG-mediated phosphorylation and activation of potassium channels hyperpolarizes SMCs, which indirectly reduces Ca^2+^ entry into SMCs (357, 364). Thus, through modulating cAMP and cGMP signaling, PDEs play critical roles in regulating SMC contractility. Altering PDE expression and activity may contribute to disorders related to vascular reactivity dysregulation, such as hypertension and erectile dysfunction. This section focuses on the roles of PDE1, 3, 4, and 5 in vascular smooth muscle reactivity (FIGURE 9).

#### 4.2.1. Dual-substrate PDEs.

In the vasculature, PDE1 is primarily expressed in SMCs but not ECs (365, 366) and regulates vascular reactivity and blood pressure. PDE1A is detected in medial SMCs in many species (18, 33, 367). Human genome-wide association studies (GWAS) show that PDE1A is associated with blood pressure dysregulation (368, 369). In a rat nitrate tolerance model induced by NO donor nitroglycerin infusion, PDE1A expression and activity increase in the aortas (370). Inhibiting PDE1 by vinpocetine partially restores the vasodilatory sensitivity of tolerant vessels to subsequent nitroglycerin exposure. Another PDE1 inhibitor, IC86340, also reduces basal blood pressure in mice (23). *Pde1a*-null mice had lower aortic blood pressure (371). PDE1B is only reported in SMCs from monkeys and baboons (367, 372), and the expression of PDE1B in contractile medial SMCs is negligible (372, 373). PDE1C expression is almost undetectable in normal medial SMCs (372, 373), which aligns with the fact that PDE1C deficiency in mice does not affect blood pressure (373). These observations suggest that the vasodilatory effect of PDE1 inhibition is primarily through inhibiting PDE1A in SMCs. PDE1A hydrolyzes cGMP with much higher affinity than it hydrolyzes cAMP (18, 28). PDE1 inhibition with vinpocetine increases cGMP levels, accompanied by dilating ex vivo rabbit and rat aortas (370, 374–377). Thus, the effect of PDE1A on vascular relaxation is likely mediated by cGMP-PKG signaling. Vasoconstrictors, such as Ang II, increase PDE1 activity via Ca^2+^ elevation (34). Ang II decreases cGMP levels (370, 378, 379), and PDE1 inhibition reverses the effect of Ang II on cGMP (370). These observations suggest that PDE1 is a critical mediator in the counterbalance between Ca^2+^ signaling (vasoconstriction) and cGMP signaling (vasorelaxation).

PDE3A is the major PDE expressed in contractile SMCs and regulates vascular contraction (380). PDE3 inhibitors effectively promote SMC relaxation in various vascular preparations (381), which appears to be independent of endothelium function (382). The drug milrinone, a PDE3 inhibitor, shows powerful vasodilatory effects by reducing total peripheral resistance, enhancing coronary blood flow, and reducing pulmonary vascular resistance in humans (381). Besides the cardiac inotropic effect, the vasodilatory effect significantly contributes to the efficacy of milrinone in the acute treatment of congestive HF. The drug cilostazol, another PDE3 inhibitor, has vasodilatory and antithrombotic properties and is an effective drug for treating chronic peripheral arterial occlusions. These pieces of clinical evidence indicate that PDE3 is important in regulating human vascular reactivity. Both PDE3A and 3B are detected in vascular SMCs (383, 384), with PDE3A predominant in regulating vascular reactivity. For example, PDE3A variants and mutations are linked to autosomal dominant hypertension and brachydactyly syndrome in patients with ischemic stroke caused by spontaneous intracranial artery dissection (385, 386). Rats carrying the human PDE3A mutant with a CRISPR/Cas9 approach recapitulate the vascular phenotypes of human patients (230). Mice with SMC-specific overexpression of mutant PDE3A have increased blood pressure (387). PDE3 inhibition relaxes vascular smooth muscle primarily by attenuating cAMP hydrolysis, thus enhancing the cAMP signaling pathway. PDE3 inhibitors have shown synergistic effects on vascular relaxation with AC activators such as forskolin (388, 389) and ISO (390, 391), which is likely attributable to PDE3A activation by PKA-mediated phosphorylation (392). In addition to posttranslational modifications, PDE3A activity or expression can be regulated by cGMP, which competitively inhibits cAMP hydrolysis by PDE3 (18, 28). Consequently, an increase in cGMP levels leads to a subsequent elevation in cAMP levels. In addition, a recent study reported that the genetic deletion of sGC-1 in vascular SMCs reduces PDE3A expression and activity (393), suggesting a different mechanism by which NO-GC-cGMP signaling negatively regulates PDE3A-modulated cAMP signaling in vascular SMCs.

#### 4.2.2. cAMP-specific PDE4.

Several PDE4 inhibitors are medications used for various medical conditions, including inflammatory diseases like chronic obstructive pulmonary disease, asthma, and psoriasis. These medications moderately affect blood pressure because of their vasodilatory properties (394), indicating the role of PDE4 in regulating vasoreactivity in humans. The effect of PDE4 inhibitors on vasodilation is also seen in rodents (395, 396). The vasorelaxant effect of PDE4 inhibition is largely contributed by PDE4D inhibition in SMCs because SMC-specific PDE4D knockout has an effect similar to PDE4 inhibition on mouse aortic contractility and blood pressure (79). Interestingly, PDE4 inhibitors relax rat aortic rings much better in the presence of a functional endothelium (382). The relaxation in the presence of endothelium is inhibited by the NOS inhibitor *N*^G^-monomethyl l-arginine (l-NMMA) and restored by the NOS substrate l-arginine, suggesting that NO is necessary for the relaxing effect of PDE4 inhibitors (382). The effect of NO on potentiating vasorelaxation by PDE4 inhibitors may be mediated by cGMP inhibition of PDE3, stimulation of cAMP-PKA signaling, and PKA phosphorylation and activation of PDE4 (397). The interaction of PDE3 and PDE4 is further supported by the fact that the combination of PDE4 and PDE3 inhibitors produces synergistic effects on SMCs (398). The effects of PDE3 and PDE4 on SMC contractility vary among different vessels. For example, the PDE4 inhibitor alone has relatively poor relaxation effects in large isolated arteries compared with the PDE3 inhibitor (381). However, PDE4 is much better at regulating vascular tone in cerebral vessels. In a canine model of acute cerebral vasospasm, PDE4 inhibitors such as denbufylline and rolipram reverse the basilar artery spasm produced by autologous blood without significantly altering mean arterial blood pressure (399). Consistently, the PDE4D gene variant is a risk factor for ischemic stroke (400). In contrast, siguazodan (a PDE3 inhibitor) produces only weak relaxation of the basilar artery (399). PDE3 inhibitors such as milrinone and amrinone are more potent relaxants of coronary arteries than of cerebral or renal arteries in dogs (401). The differences in PDE3 and PDE4 inhibition on vascular reactivity in different vessels might be due to the differential expression of PDE3 and PDE4 or the distinct endogenous stimulators of AC-cAMP or GC-cGMP signaling in different vascular beds.

#### 4.2.3. cGMP-specific PDE5.

PDE5 is present in almost all types of vascular SMCs. The vasorelaxant effects of PDE5 inhibitors vary depending on the vascular bed, possibly due to differences in local NO release and the relative contribution of PDE5 to the total amount of cGMP-hydrolyzing activity in the vascular beds. PDE5 is highly expressed in the lung and penile corpus cavernosum (402). Thus, PDE5 inhibitors have profound effects on pulmonary artery relaxation and are promising long-term therapy for treating pulmonary hypertension (PH) (403, 404). The PDE5 inhibitor sildenafil has been successfully used to treat erectile dysfunction by reinstating the impaired relaxation of SMCs within the corpus cavernosum of penile vasculature (405). It is known that the erectile response is mediated by corpus cavernosum vascular relaxation, which depends on NO release from ECs and cavernous nonadrenergic and noncholinergic (NANC) nerves in response to sexual stimulation. PDE5 inhibition facilitates a higher and sustained accumulation of cGMP in response to NO, thus enhancing the erectile action (406, 407). PDE5 has low intrinsic catalytic activity when the cGMP level is low, but PDE5 is highly activated by cGMP binding to one of the two amino-terminal GAF domains and PKG-mediated phosphorylation of the regulatory domain of PDE5 when cGMP level is elevated (291). Therefore, PDE5 inhibitors have clinically insignificant effects on systemic blood pressure and minimal effects on erection when used alone. Sexual stimulation (increasing NO-cGMP signaling) is necessary in treating erectile dysfunction with PDE5 inhibitors. Concomitant use of PDE5 inhibitors and nitroglycerin or other organic nitrates may lead to severe hypotension (408).

### 4.3. PDE in Pathological Vascular Remodeling and Diseases

Pathological vascular remodeling is a process of structural alterations in the vessel wall in response to abnormal mechanical or biological perturbations, which involves cell growth, death, migration, and extracellular matrix modulation (409–411). Pathological vascular remodeling contributes to a wide variety of circulatory system disorders, including coronary artery disease (CAD), peripheral artery disease (PVD), hypertension, aortic aneurysm (AA), and pulmonary arterial hypertension (PAH). An intact and healthy endothelium is pivotal in maintaining vascular integrity through several essential functions, including regulating vascular tone, managing vascular permeability, inhibiting platelet aggregation, preventing leukocyte adhesion and activation, and repressing vascular SMC dedifferentiation. The endothelium resides on the surface of the vessel wall and is most susceptible to a wide range of pathological stimuli and risk factors, including, but not limited to, mechanical stress, smoking, aging, hyperlipidemia, hyperhomocysteinemia, hyperglycemia, obesity, and diabetes (411, 412). Endothelium dysfunction or injury contributes to pathological vascular remodeling and vascular diseases. In a normal vessel wall, SMCs are the prominent cell type of the medial layer. These SMCs express large amounts of myofilament proteins and mainly serve to control contractility and thus are referred to as “contractile” SMCs. However, SMCs have high heterogeneity and plasticity. Upon stimulation by growth factors or inflammatory molecules, medial SMCs can undergo phenotypic modulation (dedifferentiation, e.g., from a quiescent contractile phenotype to an active myofibroblast-like phenotype, frequently referred to as a “synthetic phenotype”) (413–415). Synthetic SMCs are proproliferative and promigrative, contributing to lesion tissue growth. Synthetic SMCs are prone to senescence or apoptosis, leading to ROS production, stress-induced medial degeneration, and lesion rupture. Synthetic SMCs produce proinflammatory mediators, providing an inflammatory microenvironment for leukocyte accumulation and activation. Thus, SMC phenotype switch and aberrant activation play critical roles in pathological vascular remodeling (413–416). This section mainly focuses on the roles of PDE1, 3, 4, and 5 in vascular SMCs that contribute to pathological vascular remodeling (TABLE 4).

**Table 4.** The roles of individual PDE isoforms in vascular muscle cell proliferation

PDE Family	Isoform	Model in vivo	Model in vitro	Genetic Intervention in vivo	Genetic Intervention in vitro	Inhibitors	Inhibitory Effect (↓)	Signaling Pathway	References
PDE1									
	PDE1A		Rat aortic VSMCs		PDE1A shRNA	IC86340	↓	↑PDE1A nuclear translocation→↓cGMP→↑cyclin D1,↓p27Kip1,↓p53 ↓apoptosis	(29)
			Rat aortic VSMCs			IC86340	↓	↑nuclear PP2A-GSK3β-β-catenin →↓ β-catenin stability →↓TCF-dependent gene transcription	(417)
	PDE1C	Left common carotid artery ligation	Mouse SMCs	PDE1C^−/−^ mice	PDE1C^−/−^ SMCs	IC86340	↓	↑cAMP-PKA→↑LRP1 phosphorylation →↑PDGFRβ degradation	(373)
		Human saphenous vein explants ex vivo	Rat aortic VSMCs			IC86340	↓	↓cAMP→↓ lysosome-mediated collagen I protein degradation	(414)
PDE3			FCS/PDGF-pig aortic SMC			SK&F 94836	↓	↑cAMP →↓DNA synthesis	(418)
			FCS/PDGF/IGF-1-rat aortic VSMCs			Cilostazol	↓	↑cAMP →↓DNA synthesis	(419)
		Common carotid artery balloon injury rat	FCS/growth factors-rat carotid VSMC			Cilostamide	↓	↑cAMP →↓DNA synthesis	(420)
PDE4			FCS/PDGF-pig aortic SMC			Rolipram	↓	Combine with PDE3 inhibitor →↑cAMP →↓DNA synthesis	(418)
PDE5			Ang II-rat aortic VSMC			Sildenafil, JNJ-10258859	↓	↑cGMP	(421)
			PDGF-bovine coronary artery SMC			Sildenafil	↓	↑cGMP→↓PDE3→↑cAMP →↓DNA synthesis	(422)
			Human pulmonary artery SMC			Sildenafil	↓	↑cGMP →↓DNA synthesis	(423)
PDE10	PDE10A	Left femoral artery wire injury	PDGF-rat aortic VSMCs	PDE10A-KO	PDE10A shRNAs	MP-10 (Mardepodect, PF-2545920)	↓	↓CNP/NPR2/cGMP/PKG1α	(424)

cAMP, cyclic adenosine monophosphate; cGMP, cyclic guanosine monophosphate; CNP, C-natriuretic peptide; FCS, fetal calf serum; GSK3β, glycogen synthase kinase 3β; IGF-I, insulin-like growth factor-I; KO, knockout; LRP1, low-density lipoprotein receptor-related protein-1; NPR2, natriuretic peptide receptor 2; PDE, phosphodiesterase; PDGF, platelet-derived growth factor; PDGFRβ, PDGF receptor β; PKA, protein kinase A; PKGIα, cGMP-dependent protein kinase Iα; shRNA, short hairpin RNA; SMC, smooth muscle cell; TCF, T-cell factor; VSMC, vascular smooth muscle cell.

#### 4.3.1. cAMP-specific PDE4.

PDE4 inhibition reduces neointima formation and inhibits VCAM-1 expression and histone methylation in an Epac-dependent manner (425). The specific PDE4 isozyme contributing to neointima formation remains unknown. In human and mouse abdominal aortic aneurysm (AAA), PDE4B is upregulated primarily in inflammatory cells (426). The pan-PDE4 inhibitor rolipram decreases the incidence, aortic diameter, and rupture of AAA in mice, accompanied by reduced immune cell infiltration (426). Another pan-PDE4 inhibitor, roflumilast, also elicits a protective effect against mouse AAA development (427). PDE4D expression is upregulated in SMCs of AAA tissues (427). SMC-specific PDE4D deficiency decreases AAA in mice (427). PDE4D deficiency or inhibition attenuates SMC apoptosis in a PKA-dependent manner (427). These findings suggest that multiple PDE4 isozymes in different cell types contribute to AAA development. Meanwhile, a PDE4 inhibitor, ibudilast, inhibits cerebral aneurysms by downregulating inflammation-related molecules in the vascular wall (428). PDE4B activation exacerbates PH induced by intermittent hypoxia by enhancing mitochondrial injury and reducing cAMP-PKA-pCREB-peroxisome proliferator-activated receptor-gamma coactivator 1α (PGC-1α) signaling (429). Interestingly, the combination of PDE4 inhibitors with other cAMP-elevating agents has synergistic effects on SMC proliferation (418) and migration (398). For example, an augmented effect on attenuating SMC migration is reported when inhibiting PDE3 and PDE4 together. Inhibiting PDE3 and PDE4 activities also shows significantly improved pulmonary artery relaxation in hypoxia-induced PH (395, 430, 431). Thus, combined PDE3 and PDE4 inhibition should also be tested experimentally in other animal disease models, such as vascular hyperplasia and aortic aneurysms.

#### 4.3.2. cGMP-specific PDE5.

PDE5 is a critical negative regulator of NO-cGMP signaling. Thus, PDE5 is important for various vascular disorders associated with endothelial dysfunctions. There is evidence that PDE5 inhibition potentiates NO-mediated signaling and improves EC function (432). PDE5 inhibitors can restore EC-dependent NO bioavailability and improve the EC function in various vascular disease models, including apolipoprotein knockout (ApoE^−/−^) mice with atherosclerosis (433–435). Whereas PDE5 expression is high in contractile SMCs and reduced in synthetic SMCs, its expression can be upregulated by various pathological stimuli in synthetic SMCs and regulates the synthetic SMC functions. For example, Ang II upregulates PDE5 expression in growing rat aortic SMCs, and PDE5 inhibition reverses the Ang II-mediated inhibition of cGMP signaling and SMC growth (421). ROS generated by nicotinamide adenine dinucleotide phosphate (NADPH) oxidase upregulated PDE5 in human vascular SMCs (436). Sildenafil shows synergistic antiproliferative effects in the presence of NO donors and the GC activator BAY41-2272 in human pulmonary artery cells (423). In bovine coronary artery SMCs, sildenafil acts synergistically with NO donors to reduce platelet-derived growth factor (PDGF)-induced DNA synthesis and cell growth (422). Additionally, decreased NO bioavailability also affects platelet aggregation and vascular wall inflammation (437), which may contribute to vascular diseases and are potentially regulated by PDE5. In animal models, vardenafil attenuated rat carotid artery neointimal hyperplasia induced by endarterectomy that denuded the endothelium (438). Tadalafil inhibited rabbit carotid artery neointimal formation (439). Notably, controversial results are also reported. For example, sildenafil has no protective effects on vascular remodeling in a mouse carotid artery ligation model (440). The discrepancy may be related to the differences in species and injury models with or without EC denudation. Although most studies indicate that increased PDE5 is detrimental to normal vascular function, PDE5A level is lower in the aorta from Marfan, tricuspid, and bicuspid thoracic aneurysms, compared with healthy aorta (441, 442). Many clinical cases have reported aortic dissection after use of PDE5 inhibitor sildenafil or tadalafil (443–445). In a mouse AAA model induced by periaortic elastase treatment, PDE5A expression is significantly reduced in medial SMCs (442). Sildenafil treatment aggravated elastase-induced mouse AAA. These case reports and experimental data strongly suggest a relationship between PDE5 reduction and aortic degenerative diseases. There are many more studies focused on PDE5 in the lung vasculature because of its vasodilating and antiproliferative abilities. In human pulmonary artery SMCs (PASMCs), PDE5 accounts for 80% of cGMP hydrolysis. The inhibition of PDE5 with sildenafil elevates cGMP and attenuates pulmonary artery SMC growth (423, 446). Chronic hypoxia exposure increases PDE5 activity in the first branch and intrapulmonary arteries for cGMP hydrolysis in a rat model of PH (431). PDE5 inhibitors elicit extensive vasorelaxant effects on pulmonary vasculature (404). PDE5 inhibitors are thus a promising long-term therapy for treating PAH (403, 404). In clinical studies of humans with severe primary pulmonary hypertension, oral administration of sildenafil shows a drastic decrease in pulmonary arterial pressure and reduces pulmonary vascular resistance (403, 404). Thus, PDE5 inhibitors, such as sildenafil and tadalafil, are drugs used for treating human pulmonary arterial hypertension (447, 448).

#### 4.3.3. Dual-substrate PDEs.

PDE1A has been found in both contractile and synthetic SMCs (29). PDE1A is localized in the cytoplasm of contractile SMCs, which is primarily responsible for the regulation of SMC contractility (29). However, PDE1A is localized in the nucleus of synthetic SMCs in culture or neointimal lesions. The nuclear PDE1A is critical for synthetic SMC growth and survival (29). The differences between cytoplasmic and nuclear PDE1A and how PDE1A is translocated from the cytoplasm to the nucleus remain unknown. PDE1A promotes SMC proliferation in culture by increasing the protein stability of nuclear β-catenin, a transcription modulator of cell proliferation (417). These findings are consistent with the GWAS showing a significant association of PDE1A single-nucleotide polymorphisms with carotid intima-media thickness (368). However, experimental evidence that directly demonstrates the role of PDE1A in vascular remodeling and diseases in animal models in vivo is still lacking. Unlike PDE1A, PDE1C is barely detected in contractile SMCs. PDE1C expression is drastically elevated in growing SMCs in vitro. PDE1C is highly expressed in SMCs of neointimal and aneurysmal lesions of diseased vessels from mice and humans (372, 373, 449). PDE1C deficiency significantly attenuates SMC growth and migration in vitro (450), hallmarks of vascular hyperplasia remodeling. Indeed, injury-induced neointima formation is significantly reduced in global PDE1C-knockout mice (373). A potential mechanism is that PDE1C interacts with low-density lipoprotein receptor-related protein-1 (LRP1) and platelet-derived growth factor receptor beta (PDGFRβ), thus regulating PDGFRβ endocytosis and lysosome-dependent degradation in a cAMP-PKA-dependent manner. PDE1C inhibition also facilitates lysosome-dependent type I collagen protein degradation and thus decreases collagen contents in SMCs (414). In addition, PDE1C deficiency significantly reduces the formation and development of abdominal AA (AAA) in mice induced by Ang II infusion or periaortic elastase treatment (451). PDE1C inhibition attenuates SMC senescence via cAMP binding and activating Sirtuin 1, which plays an important role in SMC degeneration and AAA development (451). Pan-PDE1 inhibitor IC86340, which targets all PDE1 isoforms, attenuates neointimal hyperplasia and AAA in mice (373). Since PDE1 inhibitors such as ITI-214 have been proven to be safe in clinical trials, PDE1 inhibition may represent a novel therapeutic strategy for combating vascular diseases associated with pathological vascular remodeling. Changes in PDE1 expression have also been reported in PAH. For example, the cGMP-hydrolyzing PDE1 activity increases in the pulmonary artery in chronic hypoxia-induced PH rats (431). PDE1A and PDE1C expression levels are significantly upregulated in a rat pulmonary vascular remodeling model induced by sinoaortic denervation (452) and increased in human pulmonary artery SMCs from patients with idiopathic PH or secondary PH (453). PDE1C contributes to decreased cAMP and increased proliferation of pulmonary artery SMCs in patients with PH (453, 454). Methylxanthine derivatives have shown protection in different preclinical models of PH (454), although methylxanthine derivatives are not selective for PDE1. Moreover, the PDE1 inhibitor vinpocetine, either alone or in combination with other therapies such as prostacyclin analogs and NO, improves hemodynamics in the pulmonary circuit and reduces SMC proliferation in various animal PH models (455–457). More studies with specific PDE1 inhibitors or genetic deficiency mice are needed to further demonstrate the role of PDE1 isozymes in PAH.

PDE3 activity and expression significantly increase in the aortas of atherosclerosis-prone insulin-resistant rats (458). PDE3 inhibitors have been shown to block DNA synthesis in cultured pig aortic SMCs (418) and the growth of cultured SMCs in vitro (418–420). PDE3 inhibitors significantly attenuate neointima formation in a mouse model of photochemically induced vascular injury (420, 459). The PDE3 inhibitor cilostazol is an effective drug for treating symptoms of intermittent claudication caused by peripheral arterial diseases, a manifestation of systemic atherosclerosis (460). In addition, long-term oral administration of a different PDE3 inhibitor, cilostamide, suppresses arterial intimal hyperplasia by 83% in a rat balloon injury model (420). Endothelium-derived NO is critical in attenuating SMC proliferation and migration. The inhibitory effect of NO in vascular SMC mitogenesis is partly mediated by cGMP-dependent inhibition of PDE3 activity, increased cAMP level, and activation of PKA (422). PDE3 inhibition can also potentiate the effects of NO-cGMP on inflammatory molecule expression in vascular SMCs (422, 461). In addition, the PDE3 inhibitor cilostazol attenuates Ang II-induced AAA in mice and elastase-induced AAA in rats (462, 463). These findings support the important role of PDE3 in vascular occlusive diseases such as atherosclerosis and restenosis and in vascular degenerative diseases such as AAA, which suggests that PDE3 inhibition may be useful for the prevention and treatment of these diseases. A vasospasm occurs when a brain blood vessel narrows, blocking blood flow. It can occur in the 2 wk following a subarachnoid hemorrhage or brain aneurysm. PDE3 inhibitors, such as milrinone and cilostazol, effectively prevent chronic cerebral vasospasm in a canine model of chronic cerebral vasospasm, independent of systemic hemodynamic changes (464, 465). Although the increase of PDE3 activity and mRNA expression has been reported in pulmonary artery tissues from PH animals (395, 431), the functional role of PDE3 in PAH has not been well evaluated experimentally.

#### 4.3.4. Other PDEs.

There also is scattered evidence for other PDEs in pathological vascular remodeling and diseases. For example, PDE2 mRNA and protein expression are reduced in pulmonary artery SMCs from PAH patients and rats with hypoxia-induced PH (307). PDE2 inhibition with BAY60-7550 enhances cAMP-induced dilation induced by prostaglandin I_2_, supporting a potential role of PDE2 in the development of PAH. Additionally, PDE10A expression is induced in cultured SMCs and in neointimal SMC-like cells (466). PDE10A deficiency or inhibition by TP-10 (a selective PDE10 inhibitor) significantly attenuated cultured SMC proliferation (466). Consistent with this, neointima formation in the femoral artery induced by wire injury is suppressed in PDE10A-knockout mice or in mice treated with TP-10 (466). These data support the critical role of PDE10A in pathological remodeling. PDE10A may also participate in PAH. A recent study highlights a central role of PDE10A in progressive pulmonary vascular remodeling, and inhibition of PDE10A induces an accumulation of intracellular cAMP, activates cAMP response element binding protein, and attenuates PASMC proliferation and pulmonary vascular remodeling (467).

### 4.4. PDE in Endothelial Barrier Function

**FIGURE 10.**
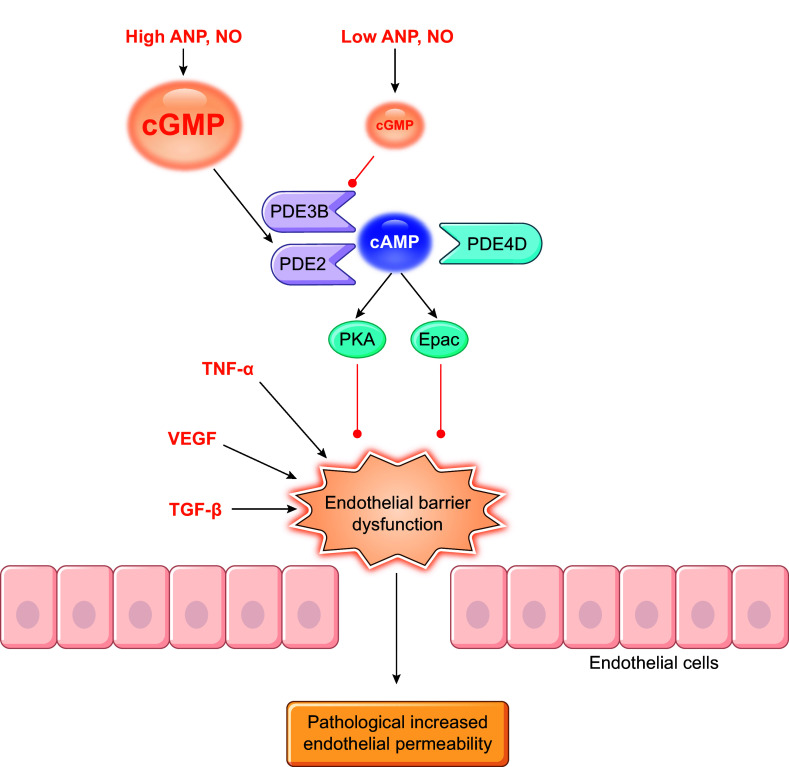
Schematic illustration of phosphodiesterases (PDEs) in endothelial cells. The cartoon highlights the roles of cyclic adenosine monophosphate (cAMP), cyclic guanosine monophosphate (cGMP), and various PDEs (PDE2, 3, and 4) in regulating endothelial cell permeability. ANP, atrial natriuretic peptide; Epac, exchange protein activated by cAMP; NO, nitric oxide; PKA, protein kinase A, TGF-β, transforming growth factor β; TNF-α, tumor necrosis factor α; VEGF, vascular endothelial growth factor.

Endothelial barrier function, such as permeability, plays a crucial role in maintaining the integrity of blood vessels and regulating the movement of substances between the bloodstream and surrounding tissues. When endothelial permeability is dysregulated, it can lead to various pathological conditions, including acute respiratory distress syndrome, sepsis, trauma, cerebral edema, etc. (468, 469). Both cAMP and cGMP signaling are important in the regulation of EC permeability. Various PDEs are expressed in ECs, although their expression may differ depending on the origin of ECs (16, 470, 471). The abnormal expression of PDEs can lead to endothelial permeability dysfunction (16). Targeting three main PDEs, PDE3, PDE4, and PDE5, in ECs can tighten the EC barrier and decrease permeability under pathological conditions (472–475). This section focuses on the roles of cAMP, cGMP, and various PDEs (PDE2, 3, and 4) in regulating EC permeability (FIGURE 10).

cAMP can regulate endothelial permeability in a PKA-dependent manner. For example, PKA activation promotes cell-cell junctions and decreases the permeability of ECs by phosphorylating vasodilator-stimulated phosphoprotein (476), negatively regulating MLC phosphorylation (477), or modulating Dll4-activated Notch signaling (478). In addition to PKA, Epac also plays an important role in regulating endothelial permeability. For example, cAMP decreases EC permeability by enhancing vascular endothelial cadherin-mediated cell adhesion, through an Epac-Rap1-dependent but PKA-independent mechanism (479). Activating the Epac-Rap1 pathway prevents VEGF-, tumor necrosis factor (TNF)-, or TGF-β-induced increase of permeability (480, 481). The two isoforms of Epac (Epac1 and 2) appear to have different roles in ECs, since genetic depletion of Epac1 but not Epac2 causes an increase in microvascular permeability in mice (482). Stimulating PGI_2_ and PGE_2_ with selective agonists improves EC permeability via both PKA- and Epac-dependent pathways (483). The differences between PKA and Epac in EC permeability regulation might be due to the differences in EC sources, such as artery ECs versus microvascular ECs. cGMP is also involved in the modulation of the barrier function of ECs. For example, activating cGMP-PKG signaling by stimulating pGC with ANP, sGC with sodium nitroprusside or a membrane-permeant cGMP analog (8-bromo-cGMP) improves EC permeability in cultured human umbilical vein endothelial cells (HUVECs) (484, 485). In addition, NO-cGMP signaling attenuates hypoxia-induced EC leakiness in vitro (486), and NPR1-cGMP-PKG signaling suppresses histamine-induced EC leakage in vivo (487). However, there are also reports of increased EC permeability by NO and ANP (488–490). Thus, the roles of cGMP signaling in regulating EC barriers could differ, even opposing, which may depend on the origins of ECs and local microenvironmental conditions in different disease statuses. The effects of cGMP on EC permeability can also be mediated through cAMP signaling. For example, cGMP reduces EC permeability through cGMP-dependently inhibiting PDE3 activity and promoting cAMP elevation (485). Interestingly, ANP or NO donors at low and high doses had opposite effects on EC permeability, which appears to be mediated by PDE3 or PDE2 differently. Low doses of ANP or NO potentiated the inhibitory effects of cAMP on thrombin-induced increase of EC permeability through cGMP-mediated inhibition of PDE3 and increase of cAMP (305). However, high doses of ANP or NO oppositely regulate EC permeability through cGMP-mediated activation of PDE2 and decrease of cAMP (305). Hence, the roles of cAMP and cGMP signaling in EC permeability regulation are more complicated by PDE2- or PDE3-mediated cross talk.

#### 4.4.1. cAMP-specific PDE4.

In addition to modulating inflammatory response, PDE4 actively regulates endothelial barrier function. PDE4D tethers Epac1 in vascular endothelial cadherin (VE-cadherin)-based complexes, thus regulating both the activity and subcellular localization of Epac1, thereby affecting EC permeability (491). Knockdown of PDE4D increases EC permeability, which is similar to the effect of Epac1 knockdown (491). PDE4 is centrally involved in the alteration of the permeability of the endothelial barrier caused by inflammation (492), thus suggesting a potential clinical use for PDE4 inhibitors during sepsis. In lung microvascular endothelium, the splice variant PDE4D4 is anchored to spectrin, a cytoskeletal protein located on the inner side of the PM. This PDE4D4 is responsible for cAMP at the membrane domains to activate barrier-enhancing effectors and prevents cAMP from accessing the cytosolic domains, where the phosphorylation of microtubule proteins by PKA induces cell gap formation (493). Importantly, PDE4 plays a critical role in regulating the permeability of the blood-brain barrier (BBB) (494, 495). PDE4 inhibitors such as BBB022 and rolipram reduce cerebrovascular endothelial permeability in the spinal cords of mice with experimental autoimmune encephalomyelitis, preventing the entry of inflammatory cells and factors and reducing tissue edema (496). The PDE4 inhibitor rolipram also maintains the expression of tight junction proteins, such as occludin and claudin-5, avoiding ischemia-induced BBB disruption in mice with stroke (472). However, rolipram can also stabilize ANP-induced disturbance of EC permeability in vivo (473). The discrepancies of PDE4 inhibition on EC permeability may be due to multiple reasons, including the inconsistencies between in vitro and in vivo models, the potential enzyme activity-independent function of PDE4, or the differences in targeting all PDE4 isoforms with pan-PDE4 inhibitors.

#### 4.4.2. Dual-substrate PDE.

PDE2 expression is relatively high in ECs (41, 432, 497). TNF-α further increases PDE2 expression in HUVECs under septic conditions (498). PDE2 inhibition reduces TNF-α- and thrombin-mediated alterations in endothelial F-actins, redistribution of VE-cadherin, and EC permeability (498). PDE2 also participates in regulating pulmonary endothelial permeability (495). Selective inhibition of PDE2 attenuates the endothelial permeability induced by the pneumococcal exotoxin pneumolysin in isolated and perfused mouse lungs and human endothelial cell monolayers (474). Collectively, these studies demonstrate the prominent role of PDE2 in modulating vascular dynamics.

Inhibition of PDE3 activity with cilostazol stabilizes the barrier integrity in primary rat brain capillary endothelial cells and the human brain hCMEC/D3 endothelial cell line (499). Cilostazol protects the BBB from damage induced by oxygen-glucose deprivation (OGD) and reoxygenation by improving the tightness of tight junctions, which PKA mediates. PDE3B is mainly expressed in these cells, presenting a target to protect against BBB pathological permeability. PDE3B tethers Epac1 and p84-regulated PI3Kγ in a signaling complex in human arterial ECs (475). A small peptide displacing Epac1 from PDE3B increases cAMP binding to Epac1 and augments PI3Kγ signaling, which modulates the endothelial barrier (475). Thus, PDE3 inhibitors may prevent the disruption of BBB.

## 5. DRUGS THAT TARGET PDEs IN CARDIOVASCULAR DISEASES

**FIGURE 11.**
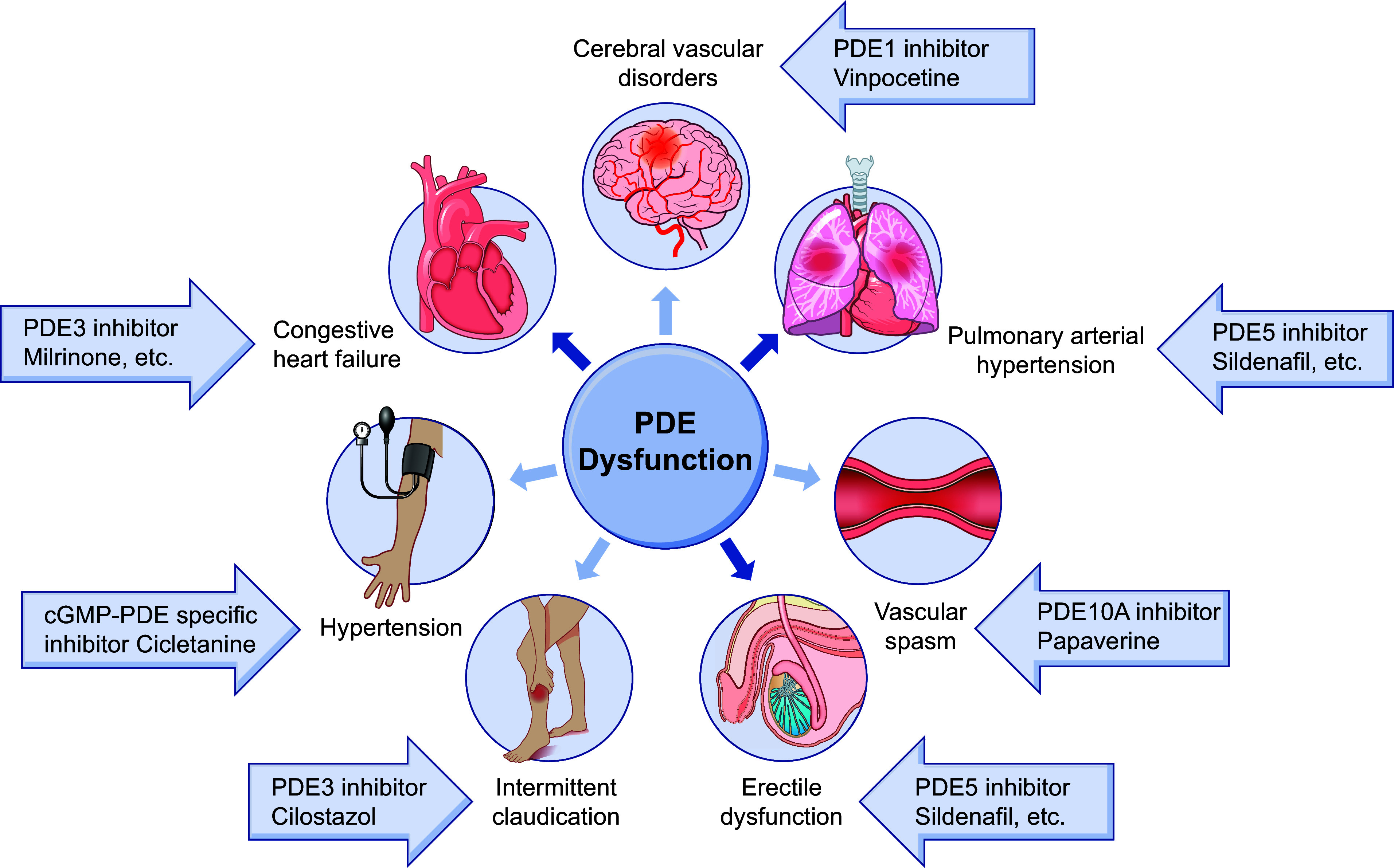
Clinical phosphodiesterase (PDE) inhibitors in cardiovascular diseases.

PDE isoforms control cAMP and cGMP, two critical intracellular second messengers regulating a variety of fundamental cellular processes in the cardiovascular system. Abnormal PDE expression and regulation have been associated with cardiovascular diseases, including cardiac hypertrophy, HF, MI, arrhythmias, hypertension, and atherosclerosis (500). Thus, targeting individual PDEs under pathological conditions is of clinical importance. For example, milrinone was one of the first PDE3 inhibitors used clinically for the therapy of acute HF. Clinical use is limited because of its detrimental side effects, such as arrhythmia in chronic HF. However, the success of sildenafil (Viagra), the first oral PDE5 inhibitor initially developed to treat angina and then approved by the Food and Drug Administration (FDA) for the treatment of male erectile dysfunction, combined with an increasing understanding of PDE biology, has raised new hopes that manipulating PDE activity with greater specificity can yield therapeutic benefits in many diseases (FIGURE 11) (501).

The contribution of each PDE family varies depending on subcellular compartments. Selectively targeting PDE isoforms within a specific location provides opportunities for greater efficacy and higher specificity in precision therapeutic strategies. Many high-affinity PDE family-selective inhibitors have shown lack of effectiveness and intolerable side effects, resulting in failed clinical trials (7). Despite this, several PDE4 inhibitors have been approved for clinical treatment such as for chronic obstructive pulmonary disorder and psoriasis (500, 502). In the future, determining the isoform-selective distribution, phosphorylation, translocation, and interactome of PDEs under pathological conditions is essential for developing isoform-selective inhibition and therapeutics. The selective PDE inhibitors used clinically or being investigated in clinical trials to treat various cardiovascular diseases are summarized in TABLE 5 and TABLE 6.

**Table 5.** Marketed PDE inhibitors in cardiovascular diseases

Drug Name	Trade Name	PDE Selectivity	Indications
Vinpocetine	Cavinton	PDE1	Cerebral vascular disorders
Cilostazol	Pletal	PDE3A, PDE4A	Intermittent claudication
Milrinone	Primacor	PDE3A, PDE4A	Congestive HF
Amrinone	Inocor	PDE3A, PDE4	Congestive HF
Enoximone	Perfan	PDE3A	Congestive HF
Olprinone	Coretec	PDE3	HF
Pimobendan	Acardi	PDE3	HF
Sildenafil	Viagra	PDE5	Erectile dysfunctionPulmonary arterial hypertension
Tadalafil	Cialis	PDE5	Erectile dysfunctionPulmonary arterial hypertension
Udenafil	Zydena	PDE5	Pulmonary arterial hypertension
Vardenafil	Levitra	PDE5	Erectile dysfunction
Avanafil	Stendra	PDE5	Erectile dysfunction
Lodenafil	Helleva	PDE5	Erectile dysfunction
Mirodenafil	Mvix	PDE5	Erectile dysfunction
Pentoxifylline	Trental	PDE4, PDE5, adenosine 2 receptors	Intermittent claudication
Cicletanine	Tenstaten	cGMP-PDE specific	Hypertension
Papaverine	Pavabid	PDE10A	Vascular spasm

FDA, food and drug administration; HF, heart failure; PDE, phosphodiesterase.

**Table 6.** Current clinical trials of PDE inhibitors in cardiovascular diseases

	Drug Name	Developer	PDE Selectivity	Indications	Highest CT Phase
Ongoing clinical development	ITI-214	Intra-Cellular Therapies	PDE1	HF (NCT03387215)	Phase 2
Discontinued or unknown clinical development	Parogrelil	Nissan Chemical Industries	PDE3, PDE5	Intermittent claudication	Phase 2
	Tetomilast	Otsuka Pharmaceutical	PDE4	HF, reperfusion injury	Phase 2
	SLx-2101	Surface Logix/Kadmon Pharmaceuticals	PDE5	Hypertension (NCT00562614) (NCT00562549)	Phase 2
	Zaprinast	Aventis	PDE5, PDE6	Ischemic heart disease	N/A

CT, clinical trial; HF, heart failure, N/A, not available; PDE, phosphodiesterase.

### 5.1. PDE Inhibitors in HFrEF

The clinical syndrome of HF includes fatigue, followed by dyspnea and exercise intolerance. HF can result from disorders of the myocardium, heart valves, or great vessels, with most patients experiencing symptoms due to impaired LV myocardial function filling or ejection of blood (503). HF may be associated with a broad spectrum of LV functional abnormalities, which can be classified as patients with normal LV size and preserved EF (EF ≥ 50; HFpEF) and patients with severe dilation and markedly reduced EF (EF ≤ 40%; HFrEF) (503). Despite sharing similar clinical phenotypes, HFpEF and HFrEF are thought to represent distinct pathophysiological entities and should be studied and treated separately (503).

In patients with HFrEF, abnormalities of systolic and diastolic dysfunction coexist. MI due to coronary artery disease is a major cause of HFrEF, and many other risk factors, such as hypertension and diabetes, may also lead to LV enlargement and HFrEF (503). HFrEF is hallmarked by cAMP and cGMP signaling defects coupled with cardiac dysfunction. PDEs degrade cAMP, restrict the sympathetic stimulation-triggered inotropic effects, and contribute to the development of systolic HF. Inhibitors of PDEs are used as positive inotropes to sensitize the heart to catecholamines (504). PDE3 and PDE4 account for most cAMP hydrolytic activities in hearts. Initial interests focus on the potential of non-isoform-selective inhibitors of PDE3 to enhance cardiac contractility and lusitropy. Although acute cAMP stimulation enhances myocardial contraction and cardiac output (505), chronic elevation of cAMP-PKA leads to cardiac remodeling and dysfunction (273, 506). Moreover, PDE3 inhibition increases the incidence of arrhythmias in HFrEF patients with excess calcium and energy demands and increases sudden death and overall mortality (58, 507–509). As a result, the short-term benefit of PDE3 inhibitors in patients with HFrEF is outweighed by an increase in sudden cardiac death when these drugs are administered chronically (264, 509). The acute benefits and chronic adverse actions of PDE3 inhibitors in HF patients may also result from the phosphorylation of different substrates of PKA in separate intracellular compartments. Meanwhile, a polymorphism in the human PDE3A promoter is reported to regulate PDE3A gene transcription by cAMP-PKA signaling and might explain the variable tolerance to milrinone in patients with HFrEF (510). To offset the unwanted side effects associated with PDE3 inhibitors, a multicenter international trial has been carried out with a combined therapy with enoximone, a PDE3 inhibitor, and beta-blockers. This trial reports no adverse impact on survival or other clinical outcomes and no significant benefit (511). A new extended-release form of milrinone (CRD-102) has been developed for advanced HF, with some early favorable clinical results (512) similar to those reported with enoximone (513). Whether this formulation alters the safety-efficacy profile of PDE3 inhibitors in chronic HFrEF therapy remains to be determined.

Apart from improving contractility in HF patients, PDE3 inhibitors are potent cardiotonic drugs that cause arterial vasodilation and afterload reduction and inhibit platelet aggregation (514). Since milrinone is predominantly excreted renally, the drug is infrequently used in patients with acute renal failure or end-stage renal diseases because of concerns about symptomatic hypotension (515). Milrinone can also be used in end-stage HF for patients resistant to optimal therapy. In addition, short-term oral PDE3 inhibition is used in severe HF patients as a bridge before they receive heart transplantation (513).

Impaired NO-cGMP signaling is known to contribute to LV diastolic abnormalities and remodeling in chronic HFrEF (516). PDE5 expression is increased in the left ventricle of patients with advanced human HF (92, 290), and PDE5 contributes to adverse ventricular remodeling after MI in mice (92). PDE5 inhibition is thus an intriguing pharmacological strategy to enhance NO-cGMP signaling in vivo. In stable HF patients with reduced left ventricle ejection fraction (EF), the PDE5 inhibitor sildenafil significantly improved LV diastolic function and cardiac geometry (516). Other small trials of PDE5 inhibition with sildenafil or udenafil have shown improvements in hemodynamics, exercise capacity, and quality of life without significant adverse events in HFrEF (517–519). However, larger phase III trials are needed to validate these findings.

Sildenafil therapy could effectively improve pulmonary hemodynamics and cardiopulmonary exercise testing measurements in patients with PH due to left heart disease (PH-LHD) with HFrEF, regardless of acute or chronic treatment (520). Additional sildenafil therapy shows symptomatic and functional improvements in PH-LHD with HFrEF, with sustained increases in EF of both the right and left ventricles (516, 519, 520). PDE5 inhibitors appear to have a dual action underlying efficacy in HFrEF: an acute effect predominantly driven by pulmonary vasodilation and a chronic effect resulting from LV remodeling independent of afterload (267). A phase IV clinical trial showed that sildenafil improves diabetic cardiomyopathy (NCT00692237) (521). The early features of diabetic cardiomyopathy are LV concentric hypertrophy associated with altered myocardial contraction dynamics. At this stage, sildenafil improves cardiac kinetics and circulating markers through an antiremodeling effect. This effect is independent of vascular, endothelial, or metabolic factors and is exerted through a direct intramyocardial action (521). Moreover, the sex difference in response to the PDE5 inhibitor tadalafil is being currently trialed in left ventricular hypertrophy associated with diabetic cardiomyopathy (NCT01803828).

Inhibition of PDE5 activity proves protective in ischemia-reperfusion injuries (501). In fact, sildenafil was initially trialed as an antianginal cardiac agent since it increased blood flow through the coronary arteries. The discovery of this “side effect” led investigators to focus on safety rather than benefit in patients with cardiovascular diseases (267). In a population of men with type 2 diabetes, the use of a PDE5 inhibitor is associated with a lower risk of overall mortality and mortality in those with a history of acute MI (316). Similar findings show that treatment with a PDE5 inhibitor might be related to a reduced risk of long-term adverse outcomes after MI (522). However, the SIDAMI trial (NCT01046838) shows that sildenafil does not decrease filling pressure at rest or during exercise in post-MI patients with diastolic dysfunction but there are effects on secondary end points, which require further comparative clinical trial studies (523).

Selective inhibition of PDE1 by ITI-214 confers acute inotropic, lusitropic, and arterial vasodilatory effects in mammals (233). A phase I/II randomized single rising dose study of ITI-214 in patients with systolic HF evaluated safety and tolerability (NCT03387215) (524). ITI-214 is generally well tolerated and acutely reduces arterial vascular resistance while augmenting cardiac inotropy, cardiac output, and heart rate. The effects of ITI-214 are dependent on cAMP modulation in adenosine signaling without calcium increase, potentially having less proarrhythmic risk than PDE3 inhibition in failing hearts (167, 233). PDE9A is upregulated in failing human hearts. PDE9 specifically regulates NPR-cGMP signaling and is independent of NO (123). Augmentation of cGMP and NPR signaling has emerged as a therapeutic strategy in HF (525). An inhibitor of PDE9, PF-04447943, seems well tolerated in humans and is currently being investigated in clinical trials for Alzheimer’s disease (NCT00930059).

### 5.2. PDE Inhibitors in HFpEF

The American College of Cardiology Foundation-American Heart Association guidelines define HFpEF as clinical signs and symptoms of HF, preserved ejection fraction, and no other apparent cause for symptoms (526). The incidence of HFpEF is increasing, accounting for almost 50% of HF cases and frequently in older women. However, the diagnosis of HFpEF remains a challenge because it is primarily based on exclusion criteria. In contrast to efficacious neurohumoral inhibition for HFrEF, therapies for HFpEF are lacking. Large trials testing neurohumoral inhibition in HFpEF failed to reach a positive primary outcome (527, 528). Recent views attribute this failure to distinct systemic and myocardial signaling in HFpEF and diverse phenotypes within the HFpEF patient population (529).

Patients with HFpEF often include older women with hypertension, T2DM, obesity, and vasculopathy, causing a systemic proinflammatory state and coronary microvascular inflammation (530). It subsequently affects LV diastolic dysfunction through macrophage infiltration, resulting in interstitial fibrosis (531), and alters paracrine communication between ECs and surrounding cardiomyocytes (530). These alterations favor hypertrophy and increase myocyte stiffness by depriving cardiomyocytes of NO and cGMP (532). Thus, organic NO donors could be useful therapeutic tools in HFpEF because they could restore myocardial NO content and concomitantly correct the high arterial load. Unfortunately, in the NEAT-HFpEF trial, the organic nitrate isosorbide mononitrate does not improve but, instead, tends to reduce chronic activity levels measured by accelerometry, with no improvement in quality of life or submaximal exercise capacity in HFpEF patients (533). This result conflicts with the NO hypothesis in HFpEF, but it should be kept in mind that organic nitrates may produce greater than expected hypotensive effects in people with HFpEF or potentially impair cardiac output because of excessive preload reduction (534). Similarly, therapy with PDE3 inhibitors is also being explored in patients with HF with a preserved ejection fraction, which is related to the capacity of PDE3 inhibition to increase cardiac output reserve at lower ventricular filling pressures during exercise (535).

Ventricular hypertrophy with interstitial fibrosis and diastolic chamber stiffening are common pathological changes in HFpEF patients. PKG stimulation has potent antifibrotic and antihypertrophic effects in experimental cardiac disease models. Multiple therapeutic approaches that stimulate PKG are currently in clinical trials or under active investigation. PDE5 primarily impacts NO-sGC-derived cGMP. Unfortunately, the largest randomized RELAX trial with PDE5 inhibition in patients with the greatest comorbidity and broadest inclusion criteria failed to show real advantages on clinical outcomes (536). In another major trial, PDE5 inhibitors had beneficial vascular effects via improved endothelium-dependent vasodilation (537), but these benefits are offset by reductions in contractility (538). Nonetheless, a positive effect of sildenafil has been reported in terms of right ventricular function and remodeling and pulmonary pressure regulation in HFpEF patients with documented PH (539). Thus, future trials should take into consideration the correct patient population based on their clinical indications to fully realize the benefits of PDE5 inhibitors for HFpEF. Meanwhile, given that depressed NO signaling may be the potential reason for the ineffective effects of PDE5 inhibitor to increase myocardial cGMP in HFpEF, inhibition of PDE9 may restore NP efficacy with beneficial effects (123), making PDE9A an attractive alternative for the treatment of HFpEF. LCZ696, a combination of the Ang II receptor inhibitor valsartan and the neprilysin inhibitor sacubitril, augmented active natriuretic peptides, resulting in an increase in cGMP in patients with HFpEF in the PARAMOUNT trial (540) and the PARAGON-HF trial (NCT01920711), offering opportunities for combined treatment with PDE9A inhibitors for HFpEF (123).

### 5.3. PDE Inhibitors in Pulmonary Hypertension and Vascular Diseases

PDE5 is abundantly expressed in lung tissue and is therefore an ideal target for treating disorders in pulmonary circulation, including PH. PDE5 inhibition promotes cGMP accumulation, enhancing NO-mediated vasodilation, and has antiproliferative effects on pulmonary artery SMCs (446). A series of trials suggest that sildenafil is beneficial in treating PH (541). The improvements in exercise capacity were largely sustained after 3 yr of treatment in the SUPER-2 study (542). Sildenafil was approved by the FDA and the European Medicines Agency (EMEA) in 2005 to treat patients with class II and III PH long term. Tadalafil has also been commercialized for treating PH to improve exercise ability and slow worsening changes in patients’ physical condition (267). In the FUEL trial, treatment with udenafil (87.5 mg twice daily) was not associated with an improvement in oxygen consumption at peak exercise but with improvements in multiple measures of exercise performance at the ventilatory anaerobic threshold (NCT02741115) (543). Meanwhile, the expressions of PDE1, 2, and 3 are increased in pulmonary artery SMCs. The inhibitors for these PDEs are effective in preclinical models of pulmonary hypertension. These PDE families may yield further therapeutic options for treating PH.

Stroke is the third leading cause of death and a major cause of disability worldwide. Endothelial dysfunction is a hallmark of small-vessel and large-artery strokes. Modulating endothelial cAMP and cGMP signaling is a potential therapeutic strategy in stroke. PDE inhibitors may restore cyclic nucleotide signals and cerebral endothelial function. The nonselective PDE1 inhibitor vinpocetine has been approved in European countries for treating dementia and stroke for >30 years. Vinpocetine is also available in the United States as a dietary supplement for improving memory and recovery from stroke through its ability to promote vasodilation by raising cGMP levels and decreasing *I*_Ca_ (544). PDE5A is localized to caveolae and modulates NOS3 activity, which can facilitate the effect of spinal cord stimulation on vasodilation and potentially prevent and treat vasospasm after subarachnoid hemorrhage (545, 546).

Preclinical studies have shown antiplatelet, vasodilator, and antiproliferative actions of the PDE3 inhibitor cilostazol (547, 548). Cilostazol has been shown to protect patients from recurrent cerebral infarction in a multicenter, randomized, placebo-controlled, double-blind clinical trial (549). Additional reports describe the pleiotropic actions of cilostazol, thereby providing a variety of clinical uses, including prevention of recurrent stroke (547, 549), cerebral vasospasm (550, 551), coronary restenosis (552), and peripheral occlusive disease. Moreover, cilostazol received FDA approval in 1999 for intermittent claudication, but off-label uses include secondary prevention of cerebrovascular accidents, percutaneous coronary intervention, and coronary stent stenosis (553). Although it offers functional improvement, cilostazol is associated with severe side effects.

Intimal hyperplasia and luminal stenosis are the key characteristics of several vascular disorders, such as atherosclerosis and postangioplasty restenosis. Several clinical trials have been designed to evaluate the benefits of the PDE3 inhibitor cilostazol. In comparison to standard antiplatelet therapy with aspirin and clopidogrel, cilostazol has been effective in attenuating postangioplasty restenosis, especially in patients at high risk of restenosis 6 mo after stent implantation [CREST trial (554)] and in patients with diabetes mellitus implanted with a drug-eluting stent [DECLARE-DIABETES trial (555)]. Compared with aspirin, cilostazol also reduces the progression of carotid atherosclerosis in patients with T2DM [DAPC trial (556)].

Overall, PDE inhibitors have been valuable tools for improving the functional status of patients with cardiovascular diseases (TABLE 5). Some of the most widely marketed PDE inhibitors fall under the diverse category of cardiovascular drugs, primarily including cardiotonics for congestive HF, vasodilatory agents for hypertension, pulmonary arterial hypertension, intermittent claudication, and cerebrovascular disorders, and antiaggregates for thrombosis-related events (557). Several initially promising therapeutic agents for cardiovascular disease have proved unsuccessful. Recently, the PDE1 inhibitor ITI-214 has completed phase I trials for HF. Additionally, a series of PDE inhibitors are under ongoing investigation in clinical or preclinical studies and predominantly aim at central nervous system (CNS) disorders, solid malignancies, and inflammatory and immune-mediated disorders (TABLE 6) (557).

## 6. NEW DIRECTIONS AND CHALLENGES IN TARGETING PDEs IN CARDIOVASCULAR DISEASES

The success of PDE5 inhibitors in treating erectile dysfunction has spurred continuous interest in investigating the effects of PDE inhibitors on cardiovascular diseases. Despite encouraging preliminary observations, several PDE inhibitors have failed in clinical trials for potential cardioprotective effects. Several factors may explain these failures.

The relationship between individual PDE isoform expression and diseases is multifaceted and complex, with different stages of cardiac diseases that may require different PDE modulations. Although PDE modulation occurs before or concurrently with the onset of disease in most preclinical studies, patients in clinical studies have established disease status (7). Recent gene profiling shows that the mRNA levels of most PDEs are downregulated in the advanced stage of human HFrEF, whereas they are either little changed or even increased in human HFpEF (558). It is also evident that many individual PDE isoforms have cell- and tissue-specific expression and are selectively localized or recruited to distinct subcellular compartments to regulate local cAMP and cGMP concentrations and specific cell functions. Lack of comprehensive insight into an individual PDE isoform’s distribution, regulation, and function prevents selectively targeting a particular isoform. Current PDE inhibitors generally target a family or subfamily of PDEs without specificity of discriminating multiple isoforms, which can be ineffective for the isoform expressed in specific cellular compartments, and are associated with unwanted side effects like arrhythmias. The overlapping structures of PDE isoforms within a family pose a challenge in designing and targeting one specific isoform with traditional small-molecule inhibitors. Hence, gene manipulation that seeks a selective and efficient approach to abrogate or enhance the activity of single PDE isoforms in a cell type-specific manner may provide a way to combat cardiovascular diseases (229).

We can also develop small molecules or peptides for disrupting specific protein-protein interactions between individual PDE splice variants and their partner, which could modulate distinct PDE pools with potentially fewer adverse effects than the global inhibition of an entire PDE family (501). An alternative approach to combating side effects attributed to systemic PDE inhibitor distribution is to develop targeted delivery systems that can preferentially transport PDE inhibitors to precise tissues or cell types. This approach can specifically disrupt the anchoring of single isoforms or splicing variants in targeted cells and tissues to reduce complications associated with systemic distribution (500). Furthermore, PDE inhibitors can act by allosteric modulation. Structural analysis of PDEs would facilitate the discovery of allosteric PDE modulators and could help correct the detrimental effects of cardiovascular diseases. Future development will aim to improve clinical outcomes with isoform-specific inhibitors and activators and with molecules modulating isoform-specific protein-protein interactions in specific signalosomes (5, 10).

Sex-dependent differences in PDE expression and function are emerging as another critical area to understand the enzymes in pathology and the implications in therapy. The mRNA expression levels of PDE1A and PDE3B are sex dependent, and the levels of PDE1A in males are higher than those in females, whereas the levels of PDE3B in males are lower than those in females (27). A recent study reveals differential regional expression of PDE4D in the apex and base of female hearts and overall higher expression of PDE4D in female hearts relative to male hearts (73). The differential PDE4D expression is linked to cAMP dynamics and action potential under adrenergic stimulation, which may be significant in sex-dependent differences in cardiac arrhythmia (73). Ovariectomy promotes mRNA of PDE4B in rat hearts (74), which may also contribute to the sex-dependent difference in ECC (75). Another report shows that the antihypertrophy effect of PDE5 inhibitor is estrogen dependent in females, though the underlying mechanism is unclear (559). A recent clinical trial indicates that PDE5 inhibitors are more effective in male patients with diabetic cardiomyopathy than in females (349). Parallel comparisons between sexes in aging animals may yield insight into the roles of PDE isoforms in the development of cardiovascular diseases and better strategies to treat patients with PDE inhibitors.

The vascular and lymphatic systems comprise structurally distinct vessels that function to circulate blood and lymph. Recently, emerging attention has been paid to the lymphatic system in health and disease (560), including cardiovascular development and disease (561). A few findings show that PDEs may be involved in lymphatic development. For example, PDE5 is expressed in lymphatic malformation tissues (562), and PDE5 inhibitors are used for severe lymphatic malformations in clinical trials (563). The role of cyclic nucleotides and PDEs in regulating the functions of lymphatic vasculature remains largely unknown, which represents a future direction of research.

The complexity of cardiovascular diseases may require combination therapies, such as multiple PDE inhibitors or simultaneously activating AC and GC and blocking PDEs, to fine-tune cyclic nucleotide levels in specific subcellular compartments, providing more effective therapies with favorable risk-benefit profiles (7, 9). In addition, the discovery of the PDE5 inhibitor sildenafil in erectile dysfunction suggests that repurposing the existing entities is an effective strategy to accelerate the development of PDE inhibitors in cardiovascular diseases. A better understanding of pharmacology and a retrospective analysis of clinical effects during trials or marketed usage for its original indication is a promising approach for finding new indications. It will also facilitate the development of novel PDE inhibitors for treating other diseases while avoiding potential cardiovascular toxicity. The challenge in addressing these issues will determine whether PDE modulators can be successfully translated to the clinical arena for treating patients with cardiovascular diseases (289, 292–296).

## GRANTS

This work was supported by National Institute of Health Grants R01GM129376, R01HL162825, and R01HL147263 and Department of Veterans Affairs Grants IK6BX005753 and I01BX005100 to Y.K.X. and by National Natural Science Foundation of China Grants 81773730 and 82273926 to Q.F.

## DISCLOSURES

No conflicts of interest, financial or otherwise, are declared by the authors.

## AUTHOR CONTRIBUTIONS

Q.F. and Y.W. prepared figures; Q.F., Y.W., C.Y., and Y.K.X. drafted manuscript; Q.F., Y.W., and Y.K.X. edited and revised manuscript; Q.F., Y.W., C.Y., and Y.K.X. approved final version of manuscript.
